# A review of clinical trial designs used to detect a disease-modifying effect of drug therapy in Alzheimer’s disease and Parkinson’s disease

**DOI:** 10.1186/s12883-016-0606-3

**Published:** 2016-06-16

**Authors:** David J. M. McGhee, Craig W. Ritchie, John P. Zajicek, Carl E. Counsell

**Affiliations:** Division of Applied Health Sciences, University of Aberdeen, Polwarth Building, Foresterhill, Aberdeen, AB25 2ZD UK; Centre for Dementia Prevention, University of Edinburgh, Edinburgh, EH8 9YL UK; School of Medicine, University of St Andrews, North Haugh, St Andrews, KY16 9TF UK

**Keywords:** Alzheimer’s disease, Parkinson’s disease, Disease progression, Biomarkers, Disease progression, Clinical trials, Neuroprotective agents

## Abstract

**Background:**

Disease-modification clinical trials in neurodegenerative disorders have struggled to separate symptomatic effects of putative agents from disease-modification. In response, a variety of clinical trial designs have been developed. A systematic review was undertaken to examine which trial designs have been used in Alzheimer’s disease (AD) and Parkinson’s disease (PD) to detect disease-modifying, as opposed to symptomatic, drug effects. In addition we aimed to identify novel clinical trial designs used in the past or planned for use in the future. We aimed to critique whether the methods used would have identified true disease-modification.

**Methods:**

MEDLINE, Embase and CENTRAL (1980–2015) were searched to identify papers meriting review in full. ClinicalTrials.gov was searched to identify unpublished or planned randomised controlled trials (RCTs). We included RCTs in PD or AD which aimed to demonstrate the disease-modifying properties of drug therapy and differentiate that benefit from any symptomatic effect.

**Results:**

128 RCTs were finally included: 84 in AD (59 published, 25 unpublished); 44 in PD (36 published, 8 unpublished). A variety of clinical trial designs were applied including long-term follow-up, wash-in and wash-out analyses, randomised delayed-start, the use of time-to-event outcome measures and surrogate disease progression biomarkers. Deficiencies in each of these design strategies, the quantity of missing data in included RCTs and the methods used to deal with missing data, meant that none of the included studies convincingly demonstrated disease-modification. No truly novel clinical trial designs were identified.

**Conclusion:**

We currently believe that the best clinical trial design available to demonstrate disease-modification is a long-term follow-up study, in which an examination is made for sustained divergence in outcome measures between treatment arms over the study period.

**Electronic supplementary material:**

The online version of this article (doi:10.1186/s12883-016-0606-3) contains supplementary material, which is available to authorized users.

## Background

Despite extensive research in neurodegenerative diseases, drugs which halt, or even slow, neurodegeneration and, therefore, clinical progression remains elusive. Whilst this failure probably in part reflects our incomplete understanding of the complex pathophysiology of neurodegenerative disorders, it also relates to difficulties in designing clinical trials to develop new disease-modifying agents.

Clinical trials in neurodegenerative disorders, including the two commonest disorders Parkinson’s disease (PD) and Alzheimer’s disease (AD), have struggled to find ways to separate out symptomatic effects of potential agents (e.g. due to increased striatal dopamine in PD, or increased acetylcholine in AD) from disease-modifying effects. Clinical assessments (e.g. in PD using motor, disability or quality of life scales) are affected/confounded by symptomatic effects of therapy and are unable to differentiate this effect from disease-modification, at least in the short-term. Researchers have, therefore, used surrogate biomarkers for disease progression (on the basis that they may be ‘immune’ to symptomatic drug effects) as the outcome measure in disease-modification trials. However, as our previous systematic reviews of biomarkers for disease progression in AD [1] and PD [2] found, there is currently insufficient evidence to recommend the use of any biomarker for disease progression as an outcome measure in disease-modification trials. Various clinical trial designs have also been developed to try to adjust for the symptomatic effects of putative disease-modifying agents and, therefore, allow clinical rating scales to be used as endpoints. These include measuring outcomes following a wash-out period [3, 4], delayed-start trial design [5], and long-term follow-up studies of placebo-treated and active-agent treated subjects.

Whilst the results of disease-modification clinical trials in PD, but not to our knowledge in AD, have been reviewed systematically [6], no previous attempt has been made to systematically review the trial designs used in such randomised controlled trials (RCTs) to separate out disease-modifying effects of putative agents from symptomatic effects. Such a review could demonstrate which (if any) of the well described advanced clinical trial designs is most effective, and highlight novel trial designs which have been successfully applied in the past or are planned for use in the near future.

We, therefore, undertook a systematic review to determine which trial designs have been used in AD and PD to detect disease-modifying, as opposed to symptomatic, drug effects. In addition we aimed to identify any novel clinical trial designs used in the past or planned for use in the future. We aimed to critique whether the methods used would have identified true disease-modification.

## Methods

As this study was a systematic review ethical approval was not required. Literature searches were conducted in the databases MEDLINE (1980 to September 2015), Embase (1980 to September 2015) and the Cochrane Central Register of Controlled Trials (CENTRAL) (searched September 2015) using the OVID search interface. Text words and index headings for AD and PD were combined with similar terms for neuroprotection and disease-modification. To help identify RCTs in MEDLINE and Embase the search outcome was then combined with the Cochrane Highly Sensitive Search Strategy for identifying RCTs in MEDLINE [7] and with search terms used by the UK Cochrane Centre for searching Embase for RCTs [8], respectively. Furthermore, in an attempt to identify completed RCTs which remained unpublished at the time of this review and those which were planned or in progress, we also searched the ClinicalTrials.gov database (searched September 2015) using their website (http://www.ClinicalTrials.gov/). Details of the electronic search strategy are given in Additional file 1.

Reference lists of relevant review articles and finally included articles were also checked to identify any studies which the electronic search strategy may have missed. We tried to identify and source publications related to relevant trials registered with ClinicalTrials.gov. We used Google to identify websites and press releases related to unpublished trials.

### Study selection

The two-stage selection procedure was performed by a single reviewer. In the first stage article titles and abstracts retrieved by the electronic search were reviewed to determine which articles merited review in full. Full length articles, where available, were then reviewed before data were extracted from relevant papers. In both stages the inclusion and exclusion criteria detailed below were applied.

#### Study design

We included RCTs in AD or PD which tried to demonstrate the disease-modifying properties of any form of drug therapy and differentiate that benefit from any symptomatic effect. Unfortunately, many studies whose aim was clearly to demonstrate disease-modification (as revealed by the methodology, discussion and conclusions) failed to state this explicitly. Therefore, to ensure we did not miss studies with potentially important clinical trial designs we included all studies where the main thrust of the article appeared to be the demonstration of disease-modification. In doing so we were aware that we risked being overly inclusive, but as we aimed to critique the clinical trial designs rather than meta-analyse the study results we did not feel this was a major problem.

Trial participants must have been followed-up for a period of at least 6 months during which they were exposed to the active-agent or placebo. We felt it highly unlikely that disease-modification could be demonstrated in a period of less than 6 months, although even this time period may be too short.

As one of the aims of this review was to identify novel clinical trial designs planned for use in the future, we included both published and unpublished RCTs. Unpublished RCTs were included to reduce the potential publication bias associated with null findings. For the purposes of this review, where sufficient information regarding a trial was available from a conference abstract or a pharmaceutical company press release then that trial was considered to be published. For each RCT with several publications we identified the most informative paper which corresponded to the main pre-specified study analysis. We, therefore, did not include extension phases to RCTs unless these were clearly pre-specified. There was no language restriction for this review.

#### Population

RCTs were only included where all participants had a diagnosis of probable AD (made using formal research criteria) or PD (made using formal research criteria or on clinical grounds). Studies which included participants with prodromal AD or mild cognitive impairment (MCI) were only included if conversion from MCI to AD was confirmed in all participants by clinical follow-up. We made no restriction on the grounds of participants’ age, disease duration, disease severity, or drug treatment.

#### Intervention

We restricted our systematic review to RCTs of non-invasive drug therapy. Trials testing intraventricular or intrathecal treatments, foetal or embryonic stem cells, striatal delivery of growth factors, or deep brain stimulation were excluded.

#### Comparison/Outcomes/Setting

We made no restriction based on the type of comparison group(s) or outcome measure(s) used in RCTs, or the trial setting.

### Data extraction

Study methods and results were extracted by a single reviewer, and to check for accuracy this was performed twice. Data were extracted using an extraction sheet (see Additional file 2) relating to the following: (1) year of publication and study location; (2) study population, including number of participants, their age, baseline measures of disease severity and baseline treatment status; (3) details of the putative agent under investigation, including its suspected mechanism of action; (4) study outcomes, including details of the primary outcome measures and any biomarkers or time-to-event outcomes used as secondary outcome measures; (5) trial design, including methods used to differentiate disease-modifying from symptomatic effects of the putative agent, methods used to deal with deaths and drop-outs in statistical analyses, and the pre-defined trial length; (6) study results, including the percentage of participants who completed the pre-defined follow-up period, reached the trial endpoint, and were included in the primary analyses; and (7) study conclusions, including whether the study investigators felt disease-modification had been demonstrated and if their conclusions were backed up by the trial methodology and results.

In this review the term ‘long-term follow-up’ was applied to studies that: (1) formally examined for sustained divergence in outcome measures between groups over time (i.e. through slope analyses); (2) did not formally measure sustained divergence but published figures (e.g. Kaplan Meier plots) from which the presence or absence of sustained divergence could be inferred; or (3) used no alternative design strategy to try to demonstrate disease-modification.

### Methodological quality

We did not formally assess study quality in terms of methods to reduce bias (e.g. methods of generating and concealing the randomisation sequence, blinding, source of funding) in this review as our primary aim was to examine what clinical trial designs have been used or are planned in RCTs to demonstrate disease-modification. However, we did examine losses to follow-up and how this was dealt with as this would clearly impact on interpretation of whether a given treatment had disease-modifying properties.

### Data analysis and synthesis

Given the descriptive nature of this systematic review extracted data was narratively synthesised. The number of participants randomised into RCTs in AD and PD was compared using the Mann–Whitney test.

## Results

**Fig. 1:**
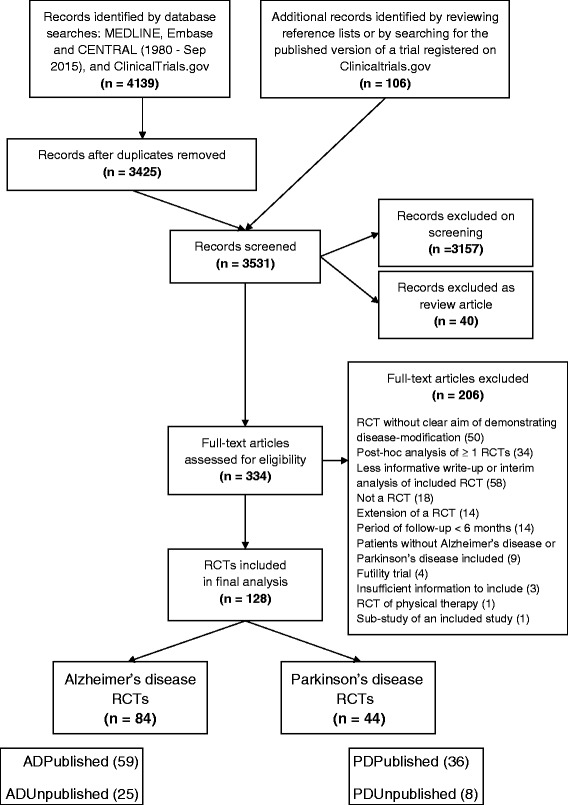
Flow diagram outlining the selection procedure to identify the 128 finally included randomised-controlled trials (RCTs)

Data were extracted from a total of 128 RCTs: 84 involving participants with AD and 44 participants with PD (Fig. 1). At the time of undertaking this review, 70 and 82 % respectively of included RCTs in AD and PD were completed and published. The reasons for exclusion of full-text articles are also detailed in Fig. 1, the most common being that the article was a less informative write-up or interim analysis of an already included RCT. Four futility studies were excluded [9–12] because, whilst these may have a preliminary role in the development of disease-modifying agents, they do not aim to differentiate disease-modifying effects of putative agents from symptomatic effects. Rather they aim to determine whether or not putative agents have any efficacy and, therefore, merit further investigation.

Additional files 3 and 4 detail the compounds investigated for disease-modifying potential in the included published RCTs in AD and PD, respectively. These tables also outline the characteristics of participants at the time of entry into each of the studies. Similarly, the details of RCTs in AD and PD which were completed but remained unpublished, were ongoing, or were yet to commence, are given in Additional files 5 and 6, respectively.

Pharmaceutical agents reputed to have anti-amyloid or anti-inflammatory properties were most commonly examined in AD RCTs, whereas in PD antioxidants were most frequently examined. The majority of studies in both disorders were conducted in centres in North America and Europe. The median pre-specified length of follow-up in the RCTs in AD was 12 (interquartile range (IQR) 6 to 18) months, and in PD 16 (IQR 12 to 24) months. However, these figures do not include trials in which the length of planned follow-up was defined as being until all participants reached a specific end-point (e.g. until all participants with PD required levodopa).

### Characteristics of participants

The total number of participants randomised in the completed RCTs in AD was 24,173 and in PD 10,652. Although the median number randomised in the AD RCTs was higher (median 313 (IQR 91 to 581)) than in the PD RCTs (median 111 (IQR 54 to 393)), this difference was not statistically significant (*P* = 0.06).

The mean age of participants in the AD RCTs was 73.4 (standard deviation (SD) 2.9) years. The vast majority of participants with AD were on symptomatic treatment at the time of study entry. Indeed, several studies stipulated that subjects had to be on treatment or commence treatment with a pre-specified cognitive enhancer to qualify for study entry. This was justified as a way of reducing the confounding effects of symptomatic treatments patients may otherwise have required during the course of the study. Disease severity scores indicated that participants in the AD RCTs had mild to moderate disease (median Mini-Mental State Examination [13] (MMSE) 20 (IQR 19 to 21), median Alzheimer’s Disease Assessment Scale – cognitive subscale [14] (ADAS-cog) 24 (IQR 22 to 26), median Washington University Clinical Dementia Rating Sum-of-Boxes score [15] (CDR-SB) 5.7 (IQR 5.3 to 5.9)).

Participants in the PD RCTs were fairly young (mean age 61.9 (SD 1.6) years). Unlike the AD RCTs, the majority of participants in the PD RCTs were not on symptomatic treatment at baseline. Many studies stipulated that participants had to be drug naïve to qualify for study entry. This stipulation was made to avoid the confounding effects of symptomatic treatment or because the time until symptomatic treatment was required was an outcome measure for the study. In one study [16], only fluctuating patients on symptomatic treatment were included and assessments were made in a practically defined off phase after 12–24 h drug withdrawal. As might be expected for disease-modification RCTs, those randomised into the PD RCTs had mild to moderate disease (median Hoehn and Yahr staging scale [17] (H&Y) 1.7 (IQR 1.5 to 1.9), median total score derived from the Unified Parkinson’s Disease Rating Scale [18] (UPDRS) 25 (IQR 21 to 29), median motor component of the Unified Parkinson’s Disease Rating Scale (UPDRS (III)) 18 (IQR 16 to 21)).

### Clinical trial designs used to demonstrate disease-modification

A summary of the methods used in an attempt to demonstrate the disease-modifying potential of pharmaceutical agents in the RCTs included in this review is presented in Table 1 and in more detail in Additional files 7 (AD) and 8 (PD).

**Table 1:** Trial designs used in included randomised controlled trials to try to demonstrate disease-modification

	Type of trials	Wash-in analysis		Wash-out analysis		Delayed-start trial design		Long-term follow-up		Biomarkers (primary or secondary outcome measures)								Time-to-event outcomes	
										Imaging		CSF		Blood		Other			
		n	%	n	%	n	%	n	%	n	%	n	%	n	%	n	%	n	%
AD	Published (*n* = 59)	3	5	11	19	7	12	45	76	27	46	17	29	6	10	2	3	3	5
	Planned, ongoing & unpublished (*n* = 25)	0	0	0	0	2	8	22	88	12	48	10	40	4	16	0	0	1	4
	All (*n* = 84)	3	4	11	13	9	11	67	80	39	46	27	32	10	12	2	3	4	5
PD	Published (*n* = 36)	11	31	17	47	7	19	25	69	12	33	0	0	1	3	0	0	18	50
	Planned, ongoing & unpublished (*n* = 8)	0	0	3	38	0	0	4	50	4	50	1	13	0	0	0	0	3	38
	All (*n* = 44)	11	25	20	45	7	16	29	66	16	36	1	2	1	2	0	0	21	48

(*CSF* Cerebrospinal fluid. ‘Other’ biomarkers include a urine biomarker and electroencephalography used in published AD trials)

In the majority of included AD RCTs more than one design strategy was used to try to demonstrate disease modification. Long-term follow-up was most frequently used (*n* = 45/59 (76 %) of included published AD studies). However, an attempt to undertake slope analyses to formally demonstrate sustained divergence was only made in one AD study designated as a long-term follow-up study in this review [19]. Trials examining the disease-modifying properties of anti-inflammatory agents (e.g. ibuprofen) most commonly did not employ an alternative design strategy. They appeared to rely on the assumption that such agents have no symptomatic effects and, therefore, any difference in primary and secondary clinical outcome measures between treatment groups after follow-up must be due to disease-modification.

Biomarkers of disease progression, most notably brain imaging (*n* = 39/84 (46 %) of included AD RCTs) and cerebrospinal fluid (CSF) biomarkers (*n* = 27/84, 32 %), were also frequently used as primary or secondary outcome measures to demonstrate disease-modification. In eleven AD studies (13 %) a wash-out analysis [3, 4] was employed to try to demonstrate disease-modification, and in three studies (4 %) a wash-in analysis [3] was used to try to show the absence of an early symptomatic effect. A randomised delayed-start design [5] was used in nine (11 %) included AD RCTs.

The most commonly used design strategy to demonstrate disease-modification in the RCTs in PD was a wash-out analysis (*n* = 20/44 (45 %) of included studies). There was, however, variability in how data relating to wash-out periods were analysed. Most frequently an examination was made for a statistically significant deterioration during the wash-out period in pre-specified clinical outcome measures in those treated with the active-agent. Additionally, in some cases, statistical techniques were used to determine whether any deterioration observed during the wash-out period in the active-agent treated group was greater than that observed in the placebo-treated group. Generally in these analyses the lack of significant deterioration in the active-agent treated group during the wash-out period, or deterioration not significantly greater than that observed in the placebo-treated group, was interpreted as meaning that any beneficial effect of the active-agent over placebo during the trial arose from disease-modification rather than a symptomatic effect. In other studies the primary efficacy analysis was conducted from baseline until the end of the wash-out period with the assumption that any observed beneficial effect of the active-agent over placebo must have been due to disease-modification. In one study of lazabemide [20], a blinded staggered withdrawal of lazabemide after either 52 or 54 weeks of treatment was undertaken. This was followed respectively by placebo for either 4 or 2 weeks. The authors stated that this staggered withdrawal was designed to allow ‘a more objective assessment of the effects of discontinuing experimental treatments’. However, the advantages of undertaking this more complicated wash-out analysis are not entirely clear.

Wash-in analyses aimed at demonstrating the absence of an early symptomatic effect of a putative agent and, thereby, providing support to assertions of a disease-modifying effect drawn from other design strategies, were employed in eleven (25 %) of the PD RCTs. In seven PD trials (16 %) a randomised delayed-start design was used, with the length of delay ranging from 6 to 9 months.

Imaging biomarkers for disease progression, namely PET and SPECT brain imaging, were used or planned for use to try to elicit a disease-modifying effect in 16 (36 %) PD RCTs. Only two trials (5 %) used or planned to use a biomarker modality other than brain imaging.

Twenty-five (69 %) of the 36 included published PD RCTs were classified as long-term follow-up studies in this review. Of these, however, only one undertook formal slope analyses to try to demonstrate sustained divergence in outcome measures between groups over time [21]. In a further 20 (56 %) published PD RCTs figures were provided which allowed the presence of sustained divergence in outcome measures between groups over time to be interpreted to some degree.

### Trial outcome measures

Additional files 9 and 10 detail the outcome measures and methods used to deal with missing data in the completed RCTs in AD and PD, respectively, in addition to providing more detailed information about the methods used to try to detect disease-modification. Similar information regarding the ongoing, planned or unpublished RCTs is given in Additional files 5 and 6.

As Table 2 displays, the majority of RCTs in AD and PD used a clinical rating scale to define their primary outcome. These trials, therefore, relied either on their design strategy or on the assumption that the putative agent had no symptomatic effects to demonstrate disease-modification.

**Table 2:** Types of primary outcome measures used in all included randomised controlled trials

Primary outcome measure	Frequency of use of primary outcome measure in randomised controlled trials in:			
	Alzheimer’s disease (*n* = 83)		Parkinson’s disease (*n* = 44)	
	n	%	n	%
Clinical outcome measure only	62	75	24	55
Clinical outcome measure and biomarker	6	7	4	9
Clinical outcome measure and time-to-event outcome	1	1	0	0
Biomarker only	12	15	5	11
Time-to-event outcome only	2	2	11	25

There was insufficient information available about one unpublished trial’s [42] outcome measures to allow its classification

Only three RCTs in AD employed a time-to-event outcome as their primary outcome measure [22–24], although another did use such a measure as a secondary outcome [25]. The time-to-event outcome measures used in AD trials related either to a specific level of clinical worsening, hospitalisation, institutionalisation or death.

The use of time-to-event outcome measures in PD RCTs was commoner, with 11 trials using such a measure as a primary outcome and ten as a secondary outcome. Most frequently the chosen time-to-event outcome was the time until levodopa or dopaminergic treatment was required on the basis of an individual patient’s level of disability. This outcome was, therefore, used as a surrogate measure of disability. In RCTs which used this outcome measure a wash-out analysis was often undertaken after reaching the study termination point (i.e. need to commence levodopa) to try to demonstrate the absence of a symptomatic effect on secondary clinical outcome measures and, therefore, prove that any beneficial effect on the time-to-event primary outcome measure was due to disease-modification.

### Methods used to deal with missing data

Additional files 11 and 12 detail the percentage of participants enrolled at baseline in each completed RCT in AD and PD who (if applicable) completed the pre-defined follow-up period, reached the trial endpoint, and were included in analyses of the primary outcome measure. In addition, the authors’ interpretation of the study results in terms of whether or not disease-modification was demonstrated is given in these additional files.

In the published RCTs in AD and PD the median percentage of participants who completed the double-blind treatment periods were 74 % (IQR 64 to 78) and 73 % (50 to 84), respectively. Of the ten published PD RCTs in which the primary outcome was defined by a time-to-event outcome a median of 64 % (40 to 83) of participants reached that outcome before the study terminated. In one of the three trials in AD where the primary outcome was defined by a time-to-event outcome (the time until clinically significant agitation or psychosis) only 17 % of participants enrolled at baseline reached that endpoint before the study ended [23].

Despite the above figures, the use of various methods to deal with missing primary outcome data, as summarised in Table 3, meant that 88 % (SD 14 %) of participants enrolled at baseline in the AD RCTs and 85 % (SD 17 %) in the PD RCTs were included in analyses of primary outcome data in these trials. Methods used included; complete-case analysis, in which only data from those who completed a trial and have complete data for a given variable are included; last observation carried forward (LOCF), in which missing data points for participants not completing a trial are replaced by the last observed value for that variable; and data imputation where missing data points are replaced by substituted values. Mixed models were more commonly used in RCTs in AD than in PD, probably reflecting the more frequent use of survival analysis in PD trials, which often employed time-to-event primary outcome measures. It is worth noting that only 36 (61 %) of the published RCTs in AD and 19 (53 %) in PD stated that they applied an intention-to-treat (ITT) approach to their statistical analyses.

**Table 3:** Methods used to deal with missing primary outcome data in included published randomised controlled trials

Method used to deal with missing data	Frequency of use in RCTs in:			
	Alzheimer’s disease (*n* = 47)		Parkinson’s disease (*n* = 31)	
	n	%	n	%
Complete-case analysis	14	30	11	35
Survival analysis	3	6	10	32
Last observation carried forward	16	34	6	19
Mixed model	18	38	4	13
Data imputation	7	15	4	13

Twelve AD trials did not report any information regarding how missing data was dealt with, four used two methods and two used three methods and one used four methods. Five Parkinson’s disease trials did not report any information regarding how missing data was dealt with and four used two methods

### Trial results

In 21 (36 %) published RCTs in AD and 17 (47 %) in PD the authors concluded that their analyses provided evidence of possible or probable disease-modifying properties (at least in some subgroups) of the agent under investigation. However, for reasons which will be discussed further below, we feel that none of these trials definitely proved that a putative agent had disease-modifying properties as opposed to simply symptomatic benefits.

## Discussion

This systematic review demonstrated that RCTs in AD and PD have used a variety of design strategies (i.e. wash-in and wash-out analyses, delayed-start designs, long-term follow-up with examination for sustained divergence in outcome measures, including time-to-event outcomes, between groups over time) either alone, in combination with one another or in conjunction with a biomarker, to try to demonstrate the disease-modifying properties of therapeutic agents over any symptomatic effect.

In the RCTs included in this review we found little evidence of novel clinical trial designs having been used in the past or planned for use in the future. Perhaps of most interest was the pragmatic approach recently used in a non-placebo controlled parallel group study of exenatide, an injectable drug approved for the treatment of diabetes, in PD [16]. By repurposing a drug to treat a novel disease development cost and time may be reduced. In this trial participants were assessed after overnight withdrawal of conventional PD medications (PD medications were allowed to reduce attrition) using blinded video assessment after a 2-month wash-out period off the study drug. The study demonstrated that those receiving exenatide improved over 14 months while those not receiving it declined. Whilst this finding could represent a prolonged symptomatic effect or, less likely, a protracted placebo effect, it does signal some possible efficacy in a cost-effective manner which may be worth pursuing in a more expensive double-blind, placebo-controlled trial.

On the basis of their trial results many authors concluded that the drug they had investigated had possible or probable disease-modifying properties. However, given the flaws in the study designs used, and problems with how missing data was handled, we feel that no study demonstrated true disease-modification. We will, therefore, discuss the merits of each design strategy in turn and outline our concerns as to how missing data was dealt with.

### Wash-in analysis

A wash-in analysis [3] compares the change between groups in clinical outcome measures over the first few weeks or months of a study. Greater improvement in active-agent than placebo-treated subjects is interpreted as indicating an early symptomatic effect, the assumption being (probably rightly) that a true disease-modifying effect would not be seen so soon: disease progression over such a short period of time would probably be minimal.

In general, wash-in analyses were used to try to demonstrate that a symptomatic effect was not associated with a given putative agent, rather than prove disease-modification in isolation. Wash-in analyses were generally combined with other design strategies (e.g. wash-out analyses) to provide additional support to claims that a given agent lacked a symptomatic effect. The conclusion then often drawn was that any difference in the primary outcome measure between groups at the end of the study must be due to disease-modification.

### Wash-out analysis

In a wash-out analysis [3, 4] treatment is withdrawn from both the active-agent and placebo-treated groups at the end of the study. The active-agent is assumed to have disease-modifying properties if those treated with it show slower disease progression throughout the double-blind treatment period than those treated with placebo, and less severe deterioration when treatment is withdrawn. However, there are a number of problems with this study design and the way in which it was employed in some of the included RCTs.

Firstly, it can be difficult to withdraw medications from patients as their symptoms may rapidly relapse and lead to their withdrawal from the trial. In wash-out analyses it is not clear how to deal with missing data from patients who withdraw during wash-out. If the data of those who withdraw is censored then analyses risk being biased in favour of those who did not experience a significant clinical deterioration following treatment withdrawal. A symptomatic effect could then be missed.

Secondly, the duration of the wash-out period required to clear all symptomatic effects of a given agent will generally not be known. Moreover, as exemplified by the differing responses to levodopa withdrawal in patients with PD (i.e. some deteriorate immediately whilst others only deteriorate after several weeks), the required period may vary considerably been individuals, making selection of a long enough wash-out period very challenging. Ultimately, carry-over of symptomatic effects may occur if the selected time is too short, and a disease-modifying effect may erroneously be reported. It is notable that on the whole the wash-out periods used in RCTs in PD were shorter (median 6 (IQR 4 to 8) weeks) than in those in AD (median 8 (IQR 6 to 12) weeks). This probably reflects the fact that, due to disabling motor symptoms, patients with PD are less able to stay off symptomatic treatment for prolonged periods of time than those with AD, rather than any considerations related to the pharmacokinetics of individual agents.

The problems of choosing too short a wash-out period were illustrated in the DATATOP trial [26]. The wash-out period of 4 to 8 weeks was inadequate because selegiline irreversibly blocks monoamine oxidase B (MAO-B) and, therefore, its actions can only be overcome by formation of new enzyme in the brain, which has a 40 day half-life [27].

Thirdly, from examining the RCTs included in this systematic review it was readily apparent that there is no consensus on how to analyse the wash-out period. In the majority of RCTs the primary study analysis was conducted from baseline until the start of the wash-out period, before a separate wash-out analysis was then conducted. In some studies this separate analysis only entailed examining for a statistically significant deterioration over the wash-out period in the active-agent treated group, while in others a comparison was made between the deterioration in both treatment groups during the wash-out period. The approach in other RCTs was simply to include the wash-out period in the primary outcome analysis: a difference in the change in primary outcome measures from the start of the study until the end of the wash-out period was examined for. These different methods of analysis make comparing the results of different studies extremely difficult.

Finally, in several included RCTs (e.g. in the PD RCT by Tetrud and Langstone [28]) a wash-out analysis using secondary outcome measures was inappropriately combined with a primary outcome analysis of a time-to-event outcome. This may mean that in some patients the full symptomatic effect of the active-agent may not have developed by the time the trial endpoint is reached. Furthermore, the symptomatic effect of a given agent may vary throughout the course of the disease which makes interpretation of the results of such wash-out analyses difficult. Combining a wash-out period with a primary outcome related to the need for dopaminergic treatment also risks some participants having to withdraw prior to the wash-out period due to the urgent need to commence symptomatic treatment. For example, in one study [28] only 70 % (38/54) of randomised subjects completed the wash-out period and 11 % (6/54) withdrew from the study immediately prior to the wash-out period or shortly thereafter.

### Randomised delayed-start trial design

In studies with a delayed-start design [5] one group of participants are randomised to receive the active-agent from the beginning, while the rest are randomised to receive placebo initially and then the active-agent at a later time point (delayed-start). In theory, if the putative agent has a purely symptomatic effect then when the second group receive the drug the progression curves for both groups should meet. However, if the compound has a purely disease-modifying effect then the progression curve of the delayed-start group will never catch up with those treated from the beginning as they will have benefited from longer exposure to the drug’s disease-modifying properties. In addition to comparing outcomes between groups at the end of the study, a sufficient number of follow-up assessments are required to allow meaningful slope analyses. However, there are several problems with the delayed-start design which make drawing conclusions about an agent’s disease-modifying properties on the results of such a trial questionable [4, 5, 29].

Firstly, a positive result in favour of the early-treatment group in a delayed-start trial may simply be due to earlier symptomatic treatment preventing deleterious compensatory mechanisms rather than specific disease modification [30].

Secondly, uncertainty exists as to how long the optimal delay for keeping patients on placebo during a delayed-started trial is. If it is too long then an unacceptably high drop-out rate may be encountered. This may lead to an imbalance in the treatment arms, selecting patients with slowly progressive disease who are able to cope for longer without any treatment. On the other hand, the treatment delay must be long enough to allow differential disease progression between the treatment arms to occur and for a given agent to reach its full symptomatic effect.

Thirdly, whilst delayed-start trials are labelled as double-blind this is perhaps only true for the first phase of the study, where patients are randomised to either placebo or active-agent [31]. Where the predetermined duration of the first phase of the trial is publically available (e.g. on ClinicalTrials.gov or in patient information leaflets), the second phase of the study may in essence become an open-label study. Furthermore, a clinical response to the active-agent in the second half of the study in those initially treated with placebo may retrospectively unblind these subjects. As clinical scoring at the end of the study is crucial, unblinding may compromise the overall study findings. It is possible to preserve blinding by performing a second randomisation to the initial placebo group, so that a proportion of patients are maintained on placebo throughout the trial [32]. However, this would result in an even more complicated study design and require a larger sample size.

Fourthly, how the results of a delayed-start trial should be analysed and interpreted is not entirely clear. Several features of a delayed-start trial can be analysed, including the difference in outcome measures between groups at the end of study, the difference in the rate of change in outcome measures between groups during the first and second phases of the study, and the size of any symptomatic response observed in wash-in analyses at the start of each study phase. How many of these analyses have to be ‘positive’ to prove disease-modification has not been determined. Furthermore, uncertainty surrounds how to deal with missing data in each phase of the study and how to appropriately power a delayed-start trial [33].

All these issues are made even more complex by the possibility that disease-modifying agents may have additional symptomatic benefits, which can lead to inconclusive results. For example, false negative results may occur if the symptomatic effect of the putative agent overwhelms any disease-modifying effect. In the ADAGIO trial [21] the disease-modifying potential of the MAO-B inhibitor rasagiline in PD was examined. ADAGIO essentially compromised two separate delayed-start trials defined by two different doses of rasagiline (1 mg and 2 mg daily). However, only the group administered the smaller dose for the full study period had significantly better outcomes at the end of the study than its respective delayed-start group. It is possible that an overwhelming symptomatic effect of rasagiline in the 2 mg per day group could account for this difference. Finally, a key assumption of delayed-start designs - that the symptomatic response at the beginning of the first and second phases are equal – may well not be true as symptomatic responses may change over time in conjunction with disease progression.

### Long-term follow-up

Well-designed long-term follow-up trials, where disease-modification is inferred from sustained divergence in outcome measures between groups over time, may well be the best current trial design to show disease modification. However, they are time consuming and expensive although their cost can be minimised by choosing outcomes which can be collected from routine data (e.g. in many countries mortality can be collected through national death registries). Uncertainty remains as to how long follow-up study should be, although ultimately this will depend on finding a balance between being sufficiently long enough to allow disease-modification to be demonstrated without unacceptably high rates of attrition. Unfortunately, the potential of many long-term follow-up studies included in this review to demonstrate disease-modification was limited by the way their results were analysed and the outcome measures employed.

Several such studies made no attempt to formally examine for sustained divergence between treatment arms and others only did so figuratively (e.g. by producing Kaplan Meier plots). In only one AD RCT was an attempt made to undertake slope analyses to formally demonstrate sustained divergence in outcome measures between treatment groups over time [18]. Long-term follow-up trials of anti-inflammatory agents in AD (e.g. ibuprofen) generally only compared outcomes between treatment groups at the end of the study. This relied on the assumption that such agents have no symptomatic effect and, therefore, any difference between groups at the end of a RCT must be due to disease-modification. This is quite a major assumption to make if a therapeutic agent is to be labelled as having disease-modifying properties. Future long-term follow-up studies must not simply compare outcome measures between groups at the end of the study but also provide clear evidence of sustained divergence in those outcome measures throughout the trial. The latter requires clinical assessments to be conducted at several time points during the trial to enable a meaningful slope analysis. Nonetheless, even slope analyses have the potential to be misleading as they assume, perhaps erroneously, linear progression of clinical outcome measures, such as the UPDRS, as the disease progresses [34]. An understanding of the change in clinical outcome measures is, therefore, crucial.

Several long-term follow-up RCTs in PD used time-to-event outcomes as the primary measure, which allows censoring of missing data in survival analysis. The most commonly used time-to-event outcome in PD was the time until levodopa or dopaminergic treatment was required on the basis of individual patient’s level of disability. Although this outcome was used as a surrogate for disability it is rather subjective. The decision to commence dopaminergic therapy depends on both patient preference and on the clinical experience of the treating physician. Furthermore, the amount of symptomatic treatment taken varies greatly between individuals (e.g. due to body weight, metabolism and levodopa phobia). Time-to-event outcomes such as mortality or reaching a specific level of clinical worsening, as used in some AD RCTs, are less subjective. The main advantage of using time until dopaminergic treatment in long-term follow-up studies in PD is clearly the avoidance of the confounding effects of concomitant symptomatic treatment. Perhaps a better solution is to permit the commencement of symptomatic treatment during a study when, and if, it is required and adjust the results in accordance with the amount taken.

### Biomarkers

As our previous systematic reviews of biomarkers for disease progression in AD [1] and PD [2] demonstrated, there is currently insufficient evidence to support the use of a biomarker as an outcome measure in RCTs in either of these disorders. The use of biomarkers in several of the included RCTs in combination with clinical outcome measures and associated trial designs is, therefore, premature and potentially misleading. However, with appropriate planning, the data required for biomarker validation (e.g. change in the biomarker over time in comparison with clinical measures, feasibility of measuring the biomarker and its acceptability to patients) may be gained from RCTs by incorporating biomarkers. It did appear that this approach was being followed in several of the included RCTs. It is also important that biomarkers used as outcome measures change in a manner congruent to the proposed therapeutic mechanism of action of the agent under investigation [35].

### Missing data

Given that more than a quarter of participants in the included RCTs in AD and PD failed to complete the double-blind treatment periods in these trials, the results of these studies may be confounded by missing data. Missing data can result in several problems, including unbalancing the treatment arms over time and introducing bias, both of which may lead to misleading inferences being drawn. Moreover, missing data reduces the overall efficacy of a study [36]. In the included RCTs a variety of methods (complete-case analysis, LOCF, data imputation, survival analysis and mixed models) were used to deal with missing primary outcome data. Whilst a detailed discussion of these methods is beyond the scope of this article, we wish to make a few key points.

Any analyses conducted in a RCT should follow the ITT principle [37], where patients are analysed according to the groups they were initially randomised to. Every patient who begins a trial should be considered to be part of that trial regardless of whether or not they finish it. ITT is, therefore, a pragmatic approach to minimise bias when estimating the effects of treatment assignment in a RCT. However, a previous review of RCTs published in major medical journals found that less than half reported using the ITT approach and, of those that did, this approach was often inadequately described and inadequately applied [38]. In keeping with these findings, only 36 (61 %) of the published RCTs in AD and 19 (53 %) in PD stated that they applied an ITT approach to their statistical analyses. Whilst in some cases this may simply reflect an omission to specifically state this, in others, where a complete-case analysis was performed, the ITT principle was clearly broken. Complete-case analysis, where only data from those who complete a study is analysed, is an approach to dealing with missing data that should be avoided. In RCTs in neurodegenerative disorders, a complete-case analysis can limit the generalisability of the study results to a minority of patients with slowly progressive disease who are most likely to be able to complete a clinical trial.

LOCF analyses were frequently used in studies included in this review to deal with missing data. For example, in a trial by Shults *et al*., [39], the primary outcome was the change in the total UPDRS over 16 months. To avoid confounding, symptomatic treatment was prohibited and, therefore, those who required dopaminergic treatment were withdrawn from the trial. To avoid these individuals having missing data at the final visit a LOCF analysis was performed. Final comparisons were, therefore, made between treatment groups that contained a range of individuals with differing periods of follow-up. A LOCF analysis, such as this, risks the difference between the groups at the predefined trial endpoint being underestimated and, therefore, a significant treatment effect being missed. Furthermore, if more participants withdraw from one treatment group than the other then a LOCF analysis may erroneously lead to the conclusion that the former group performed better, as LOCF effectively assumes that withdrawal from a RCT halts disease progression.

A more sensible strategy would be to minimise missing data, for example by choosing a primary outcome measure which is simple and easy to collect. Furthermore, many drop-outs could be avoided by permitting the commencement of symptomatic therapy during a trial, and adjusting the results on the basis of the amount of symptomatic treatment taken. As already discussed, this approach would also have avoided many of the PD long-term follow-up studies having to use ‘time-to-symptomatic treatment’ as their primary outcome measure.

Unfortunately, approaches to limit missing data are unlikely to completely eliminate this problem and, therefore, measures to account for missing data are required. Mixed modelling can be used to deal with missing data in longitudinal datasets and allow the ITT approach to data analysis to be maintained. These models take into account patients who drop-out early when estimates for later time points are made, but ensure that those with missing data do not influence the results as greatly as those with complete data. However, mixed modelling relies on the assumption that missing data is ‘missing at random’ (MAR) (e.g. due to an administrative error), which is rarely the case in a RCT where data is generally ‘missing not at random’ (MNAR), often due to undocumented disease progression. Sophisticated data imputation methods, such as multiple imputation and regression mean imputation, also assume that missing data is MAR. The analysis of RCTs with MNAR data is an extremely complex subject and a detailed discussion of solutions to this problem is beyond the scope of this article. However, it is worth noting that where assumptions are made to account for missing data then sensitivity analyses must be conducted to explore the effect of departure from those assumptions on the main study analysis [40].

## Conclusion

Given the deficiencies we have described in each of the design strategies previously used in RCTs in AD and PD, and issues relating to the quantity of missing data in these trials and how this was dealt with, none of the studies included in this review have definitively demonstrated disease-modification. Currently, there does not appear to be a clear alternative to long-term follow-up trials which analyse for sustained divergence in outcome measures between treatment arms over the time. Such trials should aim to minimise missing data/drop-outs, for example, by: allowing symptomatic treatment and adjusting the results for the amount taken and choosing a primary outcome measure which is simple and easy to collect, for example death or dependency [41]. We also recommend that any assumptions made to account for missing data in the primary analysis should be tested in sensitivity analyses.

## Abbreviations

AD, Alzheimer’s disease; H&Y, Hoehn and Yahr Staging Scale; IQR, Interquartile range; ITT, Intention-to-treat; LOCF, Last observation carried forward; MAO-B Monoamine oxidase B; MAR, Missing at random; MCI, Mild cognitive impairment; MMSE, Mini-Mental State Examination; MNAR, Missing not at random; PD, Parkinson’s disease; PET, Positron emission tomography; RCT, Randomised controlled trial; SD, Standard deviation; SPECT, Single-photon emission computerised tomography; UPDRS (III), Motor component of the Unified Parkinson’s Disease Rating Scale; UPDRS, Unified Parkinson’s Disease Rating Scale

## Additional files

Additional file 1: Electronic search strategy. (DOCX 32 kb) Additional file 2: Data collection *pro forma*. (DOCX 204 kb) Additional file 3: Baseline characteristics of participants in completed AD RCTs. (DOCX 50 kb) Additional file 4: Baseline characteristics of participants in completed PD RCTs. (DOCX 42 kb) Additional file 5: Status and key design features of ongoing or unpublished AD RCTs. (DOCX 42 kb) Additional file 6: Status and key design features of ongoing or unpublished PD RCTs. (DOCX 33 kb) Additional file 7: Methods used to differentiate symptomatic from disease-modifying drug effects in all RCTs in AD. (DOCX 57 kb) Additional file 8: Methods used to differentiate symptomatic from disease-modifying drug effects in all RCTs in PD. (DOCX 43 kb) Additional file 9: Key design features and outcome measures in published AD RCTs. (DOCX 63 kb) Additional file 10: Key design features and outcome measures in published PD RCTs. (DOCX 50 kb) Additional file 11: Outcomes of published AD RCTs. (DOCX 62 kb) Additional file 12: Outcomes of published PD RCTs. (DOCX 52 kb)
