# Supplementary material for: A review of clinical trial designs used to detect a disease-modifying effect of drug therapy in Alzheimer’s disease and Parkinson’s disease
Source: BMC Neurol. 2016 Jun 16;16:92. doi: 10.1186/s12883-016-0606-3 (PMC4910262; doi:10.1186/s12883-016-0606-3)
Supplement: Additional file 1: — Electronic search strategy. (DOCX 32 kb) [file 12883_2016_606_MOESM1_ESM.docx]

**Additional file 1: Electronic search strategy**

## 1) MEDLINE Search

1. alzheimer*.ti. or exp *alzheimer disease/
2. Parkinson*.ti. or exp *Parkinson disease/
3. 1 or 2
4. Neuroprot*.tw.
5. Disease modif*.tw.
6. Exp Neuroprotective agents/
7. 4 or 5 or 6
8. 3 and 7
9. randomized controlled trial.pt.
10. controlled clinical trial.pt.
11. randomized.ab.
12. [placebo](http://www.cochrane.org/glossary/5#term337).ab.
13. drug therapy.fs.
14. randomly.ab.
15. trial.ab.
16. groups.ab.
17. 9 or 10 or 11 or 12 or 13 or 14 or 15 or 16
18. exp animals/ not humans/
19. 17 not 18
20. 8 and 19
21. Limit 20 to yr=“1980-Current”

[Includes the sensitivity-maximizing version of the Cochrane highly sensitive search strategy for identifying randomised trials in MEDLINE (2008 version) [1]]

**Total retrieved = 2544**

## 2) Embase search

1. alzheimer*.ti. or exp *alzheimer disease/
2. Parkinson*.ti. or exp *Parkinson disease/
3. 1 or 2
4. Neuroprot*.tw.
5. Disease modif*.tw.
6. Exp Neuroprotective agents/
7. 4 or 5 or 6
8. 3 and 7
9. random*.tw.
10. factorial*.tw.
11. (crossover* or cross-over*).tw.
12. placebo*.tw.
13. (doubl* adj blind*).tw.
14. (singl* adj blind*).tw.
15. assign*.tw.
16. allocat*.tw.
17. volunteer*.tw.
18. Crossover Procedure/
19. Double-blind Procedure/
20. Randomized Controlled Trial/
21. Single-blind Procedure/
22. 9 or 10 or 11 or 12 or 13 or 14 or 15 or 16 or 17 or 18 or 19 or 20 or 21
23. Exp animal/ not human/
24. 22 not 23
25. 8 and 24
26. limit 25 to yr=“1980-Current”

[Includes search terms used by the UK Cochrane Centre for searching EMBASE for randomised controlled trials [2]]

**Total retrieved = 427**

## 3) CENTRAL search

1. alzheimer*.ti. or exp *alzheimer disease/
2. Parkinson*.ti. or exp *Parkinson disease/
3. 1 or 2
4. Neuroprot*.tw.
5. Disease modif*.tw.
6. Exp Neuroprotective agents/
7. 4 or 5 or 6
8. 3 and 7

**Total retrieved = 249**

## 4) Clinicaltrials.gov search

### a) Parkinson’s disease

Search terms: Neuroprotection or Neuroprotective or disease-modifying

Conditions: Parkinson’s disease

**Total retrieved = 85**

### b) Alzheimer’s disease

Search terms: Neuroprotection or Neuroprotective or disease-modifying

Conditions: Alzheimer’s disease

**Total retrieved = 64**

**References**

1. Cochrane Highly Sensitive Search Strategy for identifying randomized trials in MEDLINE: sensitivity-maximizing version (2008) revision; OVID format. Cochrane Handbook for Systematic Reviews of Interventions. Edited by Higgins J, Green S. 2011. The Cochrane Collaboration. http://www.cochrane-handbook.org. Accessed 14 Oct 2015.
2. Lefebvre C, Eisinga A, McDonald S, Paul N. Enhancing access to reports of randomized trials published world-wide--the contribution of EMBASE records to the Cochrane Central Register of Controlled Trials (CENTRAL) in The Cochrane Library. Emerg Themes Epidemiol. 2008;5:13.
