# Supplementary figures and images for: A review of clinical trial designs used to detect a disease-modifying effect of drug therapy in Alzheimer’s disease and Parkinson’s disease
Source: BMC Neurol. 2016 Jun 16;16:92. doi: 10.1186/s12883-016-0606-3 (PMC4910262; doi:10.1186/s12883-016-0606-3)

**Additional file 2: Data collection *pro forma***

**
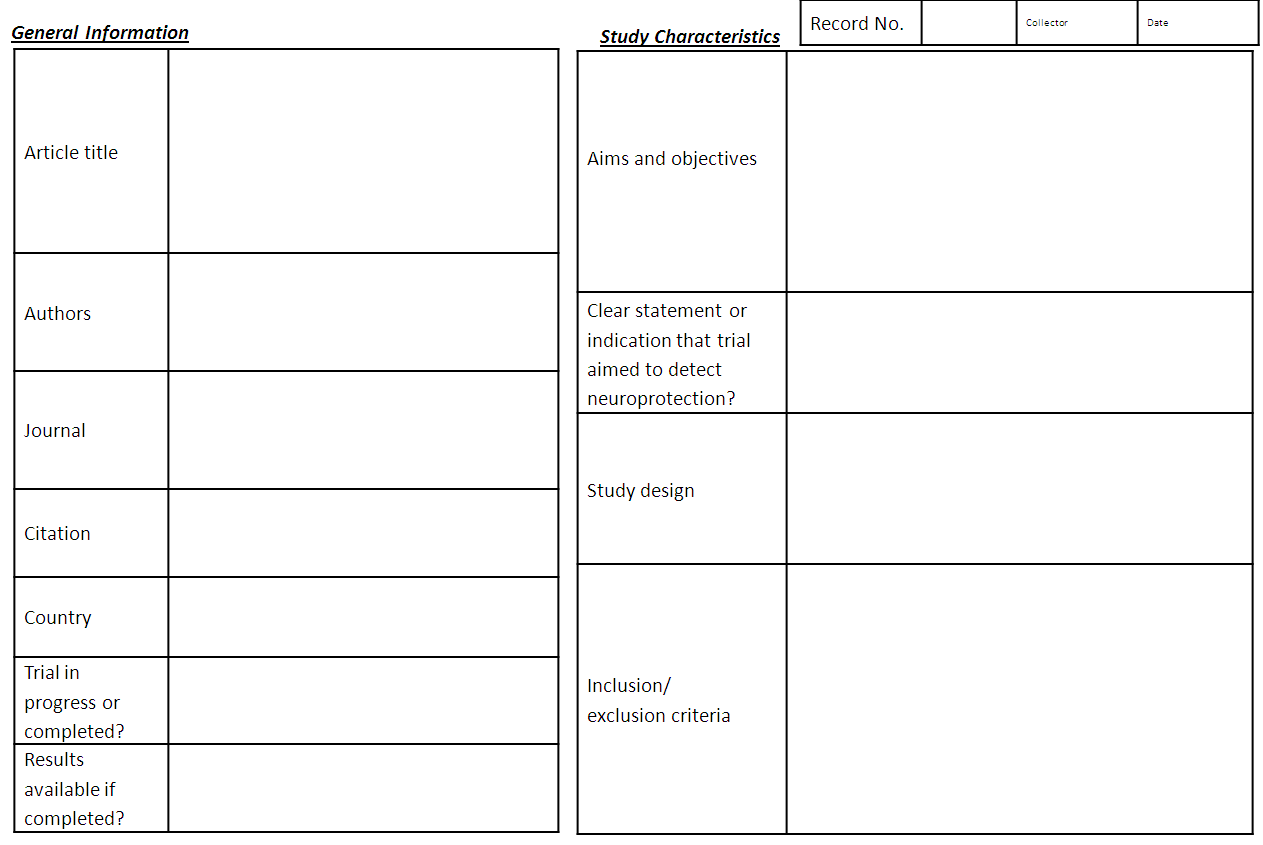
**

**
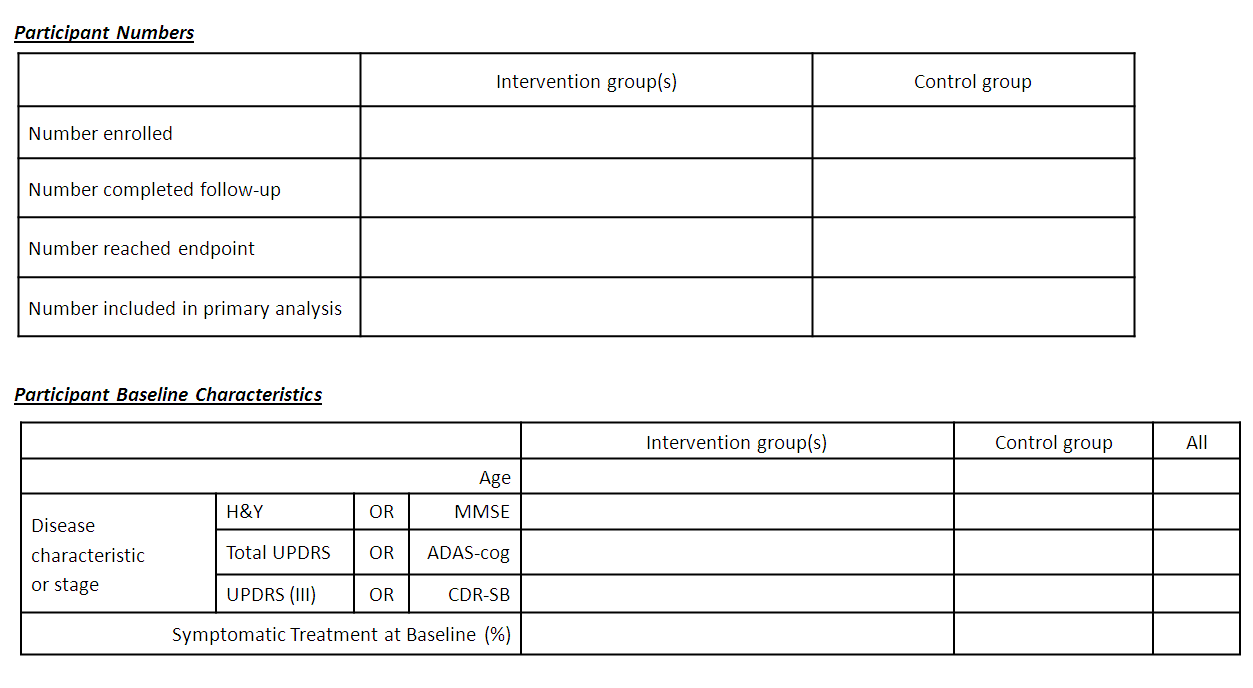
**

**
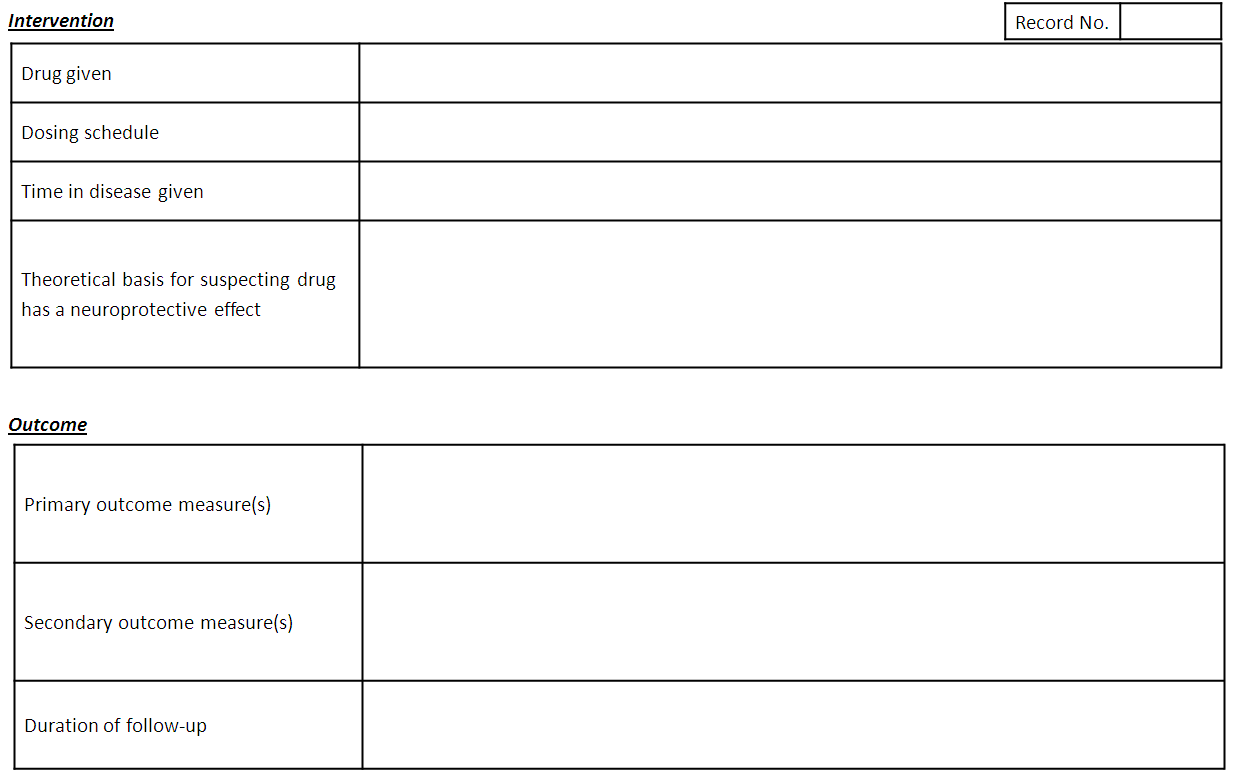
**

**
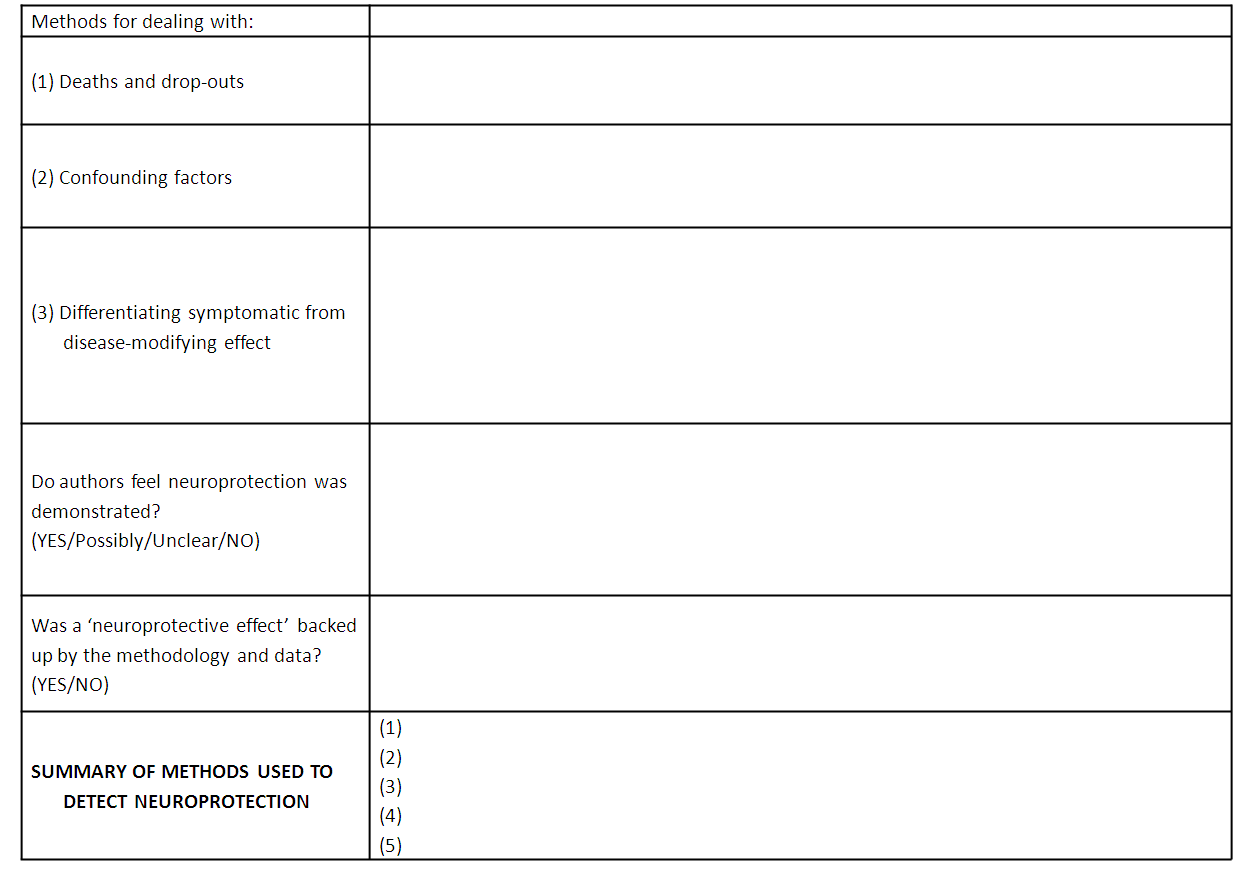
**

Supplement: Additional file 2: — Data collection pro forma. (DOCX 204 kb) [file 12883_2016_606_MOESM2_ESM.docx]
