# Supplementary material for: A review of clinical trial designs used to detect a disease-modifying effect of drug therapy in Alzheimer’s disease and Parkinson’s disease
Source: BMC Neurol. 2016 Jun 16;16:92. doi: 10.1186/s12883-016-0606-3 (PMC4910262; doi:10.1186/s12883-016-0606-3)
Supplement: Additional file 3: — Baseline characteristics of participants in completed AD RCTs. (DOCX 50 kb) [file 12883_2016_606_MOESM3_ESM.docx]

**Additional file 3: Baseline characteristics of participants in completed randomised controlled trials of putative disease-modifying agents in Alzheimer’s disease**

| **Trial** | **Year published** | **Location** | **Active agent(s)** | **Putative mechanism(s)** | **Number of participants randomised** | **Characteristics of participants at baseline** | | | | | |
| --- | --- | --- | --- | --- | --- | --- | --- | --- | --- | --- | --- |
|  |  |  |  |  |  | **Age** | **MMSE** | **ADAS-cog** | **CDR-SB** | **% on symptomatic treatment** | |
| Aβ immunisation [1] | 2005 | USA/Europe | AN1792 | Anti-amyloid | 372 | 72 | 20 | 23 | - | >59% |  |
| LEADe [2] | 2010 | International | Atorvastatin | Anti-amyloid | 640 | 74 | 22 | 22 | 5.8 | 100% | (n=640) |
| Bapineuzumab (phase 3)  APOE ε4 carriers [3] | 2012 | USA | Bapineuzumab | Anti-amyloid | 1121 | 72 | 21 | 24 | - | 92% | (n=1006) |
| Bapineuzumab (phase 3) APOE ε4 non-carriers [3] | 2012 | USA | Bapineuzumab (2 dosages) | Anti-amyloid | 1331 | 73 | 21 | 22 | - | 90% | (n=1001) |
| Bapineuzumab (phase 2) [4] | 2009 | USA | Bapineuzumab (4 dosages) | Anti-amyloid | 234 | 69 | 21 | - | - | 96% | (n=219) |
| ABBY [5, 6] | 2014 § | USA | Crenezumab (2 dosages) | Anti-amyloid | 431 | - | - | - | - | Unclear |  |
| BLAZE [6-8] | 2014 § | USA | Crenezumab (2 dosages) | Anti-amyloid | 91 | - | - | - | - | Unclear |  |
| Scyllo-inositol [9] | 2011 | North America | Scyllo-inositol (ELND005)  (3 dosages) | Anti-amyloid | 353 | 73 | 20 | 23 | 5.3 | 91% | (n=318) |
| IDENTITY [10, 11] | 2013 | International | Semagacestat (LY450139) | Anti-amyloid | 1537 | 73 | 21 | 23 | - | 100% | (n=1537) |
| IDENTITY2 [12] | 2013 ‡ | International | Semagacestat (LY450139) | Anti-amyloid | 1111 | 73 | - | 24 | - | 100% | (n=1111) |
| Simvastatin [13] | 2002 | Germany | Simvastatin | Anti-amyloid | 44 | 68 | 17 | 31 | - | Unclear |  |
| EXPEDITION 1 [14] | 2014 | International | Solanezumab (LY2062430) | Anti-amyloid | 1012 | 75 | 21 | 22 | 5.0 | 88% | (n=894) |
| EXPEDITION 2 [14] | 2014 | International | Solanezumab (LY2062430) | Anti-amyloid | 1040 | 72 | 21 | 24 | 5.3 | 91% | (n=943) |
| Tarenflurbil (phase 3 )[15] | 2009 | International | Tarenflurbil (2 dosages) • | Anti-amyloid | 1684 | 75 | 23 | 26 | 4.9 | 80% | (n=1342) |
| Tarenflurbil (phase 2) [16] | 2008 | Canada/UK | Tarenflurbil (2 dosages) | Anti-amyloid | 210 | 75 | 21 | 31 | 6.4 | 95% | (n=179/189) |
| Alphase [17] | 2011 | North America | Tramiprosate (2 dosages) | Anti-amyloid | 1052 | 74 | 21 | 22 | 5.7 | 100% | (n=1052) |
| DARAD [18] | 2013 | Canada | Doxycycline and Rifampicin | Anti-amyloid and  anti-tau | 406 | 79 | - | - | - | >93% |  |
| T-817MA [19] | 2013 Φ | North America | T-817MA | Anti-amyloid and promotes neurogenesis | 373 | - | - | - | - | 100% | (n=373) |
| Celecoxib [20] | 2007 | International | Celecoxib | Anti-inflammatory | 425 | 74 | - | 25 | - | Unclear |  |
| DAD2000 [21] | 2002 § | International | Dapsone | Anti-inflammatory | 201 | - | - | - | - | Unclear |  |
| Diclofenac + misoprostol [22] | 1999 | Australia | Diclofenac | Anti-inflammatory | 41 | 73 | 18 | - | - | Unclear |  |
| Docosahexaenoic acid [23] | 2010 | USA | Docosahexaenoic acid | Anti-inflammatory | 402 | 76 | 21 | 24 | 5.7 | ≥ 86% |  |
| OmegAD [24] | 2006 | Sweden | Docosahexaenoic acid and eicosapentaenoic acid | Anti-inflammatory | 204 | 73 | 23 | 26 | 5.9 | 100% | (n=204) |
| Escitalopram [25] | 2012 Φ | South Korea | Escitalopram | Anti-inflammatory and  promotes neurogenesis | 74 | - | - | - | - | Unclear |  |
| **Trial** | **Year published** | **Location** | **Active agent(s)** | **Putative mechanism(s)** | **Number of participants randomised** | **Characteristics of participants at baseline** | | | | | |
|  |  |  |  |  |  | **Age** | **MMSE** | **ADAS-cog** | **CDR-SB** | **% on symptomatic treatment** | |
| Hydroxychloroquine [26] | 2001 | Netherlands | Hydroxychloroquine | Anti-inflammatory | 168 | 71 | - | 18 | - | Unclear |  |
| Ibuprofen [27] | 2009 | Italy | Ibuprofen | Anti-inflammatory | 132 | 74 | 20 | 26 | 4.5 | 100% | (n=132) |
| Dutch indomethacin [28] | 2008 | Netherlands | Indomethacin | Anti-inflammatory | 51 | 72 | 19 | 20 | - | Unclear |  |
| American indomethacin [29] | 1993 | USA | Indomethacin | Anti-inflammatory | 44 | 78 | - | - | - | Unclear |  |
| Masitinib (phase 2) [30] | 2011 | France | Masitinib | Anti-inflammatory | 34 | 73 | 19 | 20 | - | 100% | (n=34) |
| Prednisone [31] | 2000 | USA | Prednisone | Anti-inflammatory and immunosuppressant | 138 | 73 | 22 | 22 | 5.4 | Unclear |  |
| Resveratrol [32] | 2015 | USA | Resveratrol | Anti-oxidant | 119 | 71 | 20 | 25 | 5.2 | 100% | (n=119) |
| Rofecoxib [33] | 2004 | USA | Rofecoxib | Anti-inflammatory | 692 | 76 | 21 | 21 | 5.6 | 58% | (n=401) |
| Rofecoxib or naproxen [34] | 2003 | USA | Rofecoxib or Naproxen | Anti-inflammatory | 351 | 74 | 21 | 24 | 5.7 | 68% | (n=238) |
| DAV.I.D.E. [35] | 1998 | Italy | Dihydroergokryptine | Antioxidant | 215 | 74 | 20 | - | - | 0% | (n=0) |
| Nutritional formulation [36] | 2014 | USA | Nutraceutical formulation * | Antioxidant | 143 | 78 | 22 | - | - | Unclear |  |
| Czech/Slovak selegiline [37] | 1999 | Czech Rep/Slovakia | Selegiline | Antioxidant | 173 | 83 | 19 | - | - | Unclear |  |
| Canadian selegiline [38] | 1998 | Canada | Selegiline | Antioxidant | 60 | 70 | 18 | - | - | Unclear |  |
| KUOSTAD [39] | 1995 Φ | Finland | Selegiline | Antioxidant | 80 | - | 19 | - | - | Unclear |  |
| Nebraska selegiline [40] | 1993 | USA | Selegiline | Antioxidant | 39 | 73 | 19 | - | 6.0 | Unclear |  |
| Selegiline & tocopherol [41] | 1997 | USA | Selegiline, α-tocopherol or both agents | Antioxidant | 341 | 73 | 13 | - | 11.0 | 0% | (n=0) |
| VALID [42] | 2011 | USA | Divalproex sodium (valproate) | Anti-tau | 313 | 76 | 17 | 30 | 7.3 | >91% |  |
| TauRx (phase 2) [43] | 2015 | UK/Singapore | Methylthioninium  (3 dosages) | Anti-tau | 321 | 74 | 19 | 25 | - | 0% | (n=0) |
| Donepezil MRI/MRS [44] | 2003 | USA | Donepezil | Cholinesterase inhibitor | 67 | 73 | 19 | 26 | - | 0% | (n=0) |
| Donepezil international [45] | 1999 | International | Donepezil (2 dosages) | Cholinesterase inhibitor | 818 | 72 | 20 | - | - | 0% | (n=0) |
| Donepezil USA clinical [46] | 1998 | USA | Donepezil | Cholinesterase inhibitor | 473 | 73 | 19 | - | - | 0% | (n=0) |
| Galantamine [47] | 2000 | USA | Galantamine (2 dosages) | Cholinesterase inhibitor | 636 | 75 | 19 | 20 | - | 0% | (n=0) |
| GAP Study [48, 49] | 2013 ‡Φ | North America | Intravenous immunoglobulin (2 dosages) | Immune modulator | 390 | 70 | 21 | - | - | 100% | (n=390) |
| CONCERT [50, 51] | 2012 † | International | Dimebon (Latrepirdine)  (2 dosages) | Mitochondrial stabiliser | 1003 | - | - | - | - | 100% | (n=1003) |
| CONNECTION [52, 53] | 2010 § | International | Dimebon (Latrepirdine)  (2 dosages) | Mitochondrial stabiliser | 598 | 74 | 18 | - | - | 0% | (n=0) |
| Russian Dimebon [54] | 2008 | Russia | Dimebon (Latrepirdine) | Mitochondrial stabiliser | 183 | 68 | 18 | 30 | - | 0% | (n=0) |
| Cerebrolysin [55] | 2006 | Europe | Cerebrolysin (3 dosages) | Neurotrophic | 279 | 74 | 20 | 36 | - | 0% | (n=0) |
| **Trial** | **Year published** | **Location** | **Active agent(s)** | **Putative mechanism(s)** | **Number of participants randomised** | **Characteristics of participants at baseline** | | | | | |
|  |  |  |  |  |  | **Age** | **MMSE** | **ADAS-cog** | **CDR-SB** | **% on symptomatic treatment** | |
| Memantine PET [56] | 2013 | China | Memantine | NMDA receptor antagonist | 26 | 65 | 12 | 43 | - | 0% | (n=0) |
| Memantine MRI [57] | 2012 | Europe | Memantine | NMDA receptor antagonist | 278 | 74 | 17 | - | - | 72% | (n=198/275) |
| Memantine MRS [58] | 2011 | USA | Memantine | NMDA receptor antagonist | 17 | 76 | 21 | 45 | - | Unclear |  |
| Memantine vs. donepezil MRS [59] | 2010 | USA | Memantine | NMDA receptor antagonist | 67 | 77 | - | 23 | - | 0% | (n=0) |
| Memantine multimodal [60] | 2008 | Austria | Memantine | NMDA receptor antagonist | 37 | 76 | 19 | 28 |  | 0% | (n=0) |
| REFLECT-1 [61] | 2010 | International | Rosiglitazone (2 dosages) | PPAR-γ agonist | 581 | 72 | 19 | 26 | - | 0% | (n=0) |
| Rosiglitazone genetics [62] | 2006 | Europe/New Zealand | Rosiglitazone (3 dosages) | PPAR-γ agonist | 518 | 70 | 21 | - | - | 0% | (n=0) |
| Azeliragon [63] | 2014 | USA | Azeliragon (2 dosages) | RAGE antagonist | 399 | 73 | 20 | 24 | 5.8 | 100% | (n=399) |

(ADAS-cog , Alzheimer’s Disease Assessment Scale – cognitive subscale [64]; CDR-SB, The Washington University Clinical Dementia Rating Sum-of-Boxes score [65]; MMSE, Mini-Mental State Examination [66]; NMDA, N-methyl-D-aspartate; PPAR-γ, Peroxisome proliferator-activated-receptor-γ; RAGE, Receptor for Advanced Glycation Endpoints)

† Results available in commentary article, but not yet published as a paper.

‡ Results available on ClinicalTrials.gov, but not yet published as a paper.

§ Results available online as a press release, but not yet published as a paper.

• Initially patients were assigned to treatment with 400mg or 800mg of tarenflurbil twice daily. After an analysis of phase two data indicated that patients with mild Alzheimer’s disease had a more robust response to 800mg twice daily all patients on 400mg twice daily were switched to 800mg twice daily. In subsequent analyses all patients taking tarenflurbil were treated as a single group.

Φ Published as a conference abstract, but not yet published as a paper.

* Formulation contains folate, alpha-tocopherol, vitamin B12, S-adenosyl methioinine, N-acetyl cysteine and acetyl-L-carnitine.

**References**

1. Gilman S, Koller M, Black RS, Jenkins L, Griffith SG, et al. Clinical effects of Abeta immunization (AN1792) in patients with AD in an interrupted trial. Neurology. 2005;64:1553-62.
2. Feldman HH, Doody RS, Kivipelto M, Sparks DL, Waters DD, Jones RW, et al. Randomized controlled trial of atorvastatin in mild to moderate Alzheimer disease: LEADe. Neurology. 2010;74:956-64.
3. Salloway S, Sperling R, Fox NC, Blennow K, Klunk W, Raskind M, et al. Two phase 3 trials of bapineuzumab in mild-to-moderate Alzheimer's disease. N Engl J Med. 2014;370:322-33.
4. Salloway S, Sperling R, Gilman S, Fox NC, Blennow K, Raskind M, et al. A phase 2 multiple ascending dose trial of bapineuzumab in mild to moderate Alzheimer disease. Neurology. 2009;73:2061-70.
5. A Study to Evaluate the Efficacy and Safety of MABT5102A in Patient With Mild to Moderate Alzheimer's Disease (ABBY). ClinicalTrials.gov. 2015. http://www.clinicaltrials.gov/ct2/show/NCT01343966. Accessed 9 Oct 2015.
6. Roche announces phase II clinical results of crenezumab in Alzheimers disease. Roche. 2014. http://www.roche.com/investors/updates/inv-update-2014-07-16.htm. Accessed 12 Oct 2015.
7. A Study to Evaluate the Impact of MABT5102A on Brain Amyloid Load and Related Biomarkers in Patients with Mild to Moderate Alzheimer's Disease. ClinicalTrials.gov. 2015. http://www.clinicaltrials.gov/ct2/show/NCT01397578. Accessed 9 Oct 2015.
8. AC Immune receives milestone payment for crenezumab moving into phase III clinical development in Alzheimer's disease. Swiss Biotech. 2015. http://www.swissbiotech.org/b/index.php?1=1&id=665679. Accessed 12 Oct 2015.
9. Salloway S, Sperling R, Keren R, Porsteinsson AP, van Dyck CH, Tariot PN, et al. A phase 2 randomized trial of ELND005, scyllo-inositol, in mild to moderate Alzheimer disease. Neurology. 2011;77:1253-62.
10. Effect of LY450139 on the Long Term Progression of Alzheimer's Disease. ClinicalTrials.gov. 2015. http://www.clinicaltrials.gov/ct2/show/NCT00594568. Accessed 9 Oct 2015.
11. Doody RS, Raman R, Farlow M, Iwatsubo T, Vellas B, Joffe S, et al. A phase 3 trial of semagacestat for treatment of Alzheimer's disease. N Engl J Med. 2013;369:341-50.
12. Effect of LY450139, on the progression of Alzheimer's disease as compared with placebo (IDENTITY-2). ClinicalTrials.gov. 2015. http://www.clinicaltrials.gov/ct2/show/NCT00762411. Accessed 22 Sep 2015.
13. Simons M, Schwarzler F, Lutjohann D, von BK, Beyreuther K, Dichgans J, et al. Treatment with simvastatin in normocholesterolemic patients with Alzheimer's disease: A 26-week randomized, placebo-controlled, double-blind trial. Ann Neurol. 2002;52:346-50.
14. Doody RS, Thomas RG, Farlow M, Iwatsubo T, Vellas B, Joffe S, et al. Phase 3 trials of solanezumab for mild-to-moderate Alzheimer's disease. N Engl J Med. 2014;370:311-21.
15. Green RC, Schneider LS, Amato DA, Beelen AP, Wilcock G, Swabb EA, et al. Effect of tarenflurbil on cognitive decline and activities of daily living in patients with mild Alzheimer disease: a randomized controlled trial. JAMA. 2009;302:2557-64.
16. Wilcock GK, Black SE, Hendrix SB, Zavitz KH, Swabb EA, Laughlin MA. Efficacy and safety of tarenflurbil in mild to moderate Alzheimer's disease: a randomised phase II trial. Lancet Neurol. 2008;7:483-93.
17. Aisen PS, Gauthier S, Ferris SH, Saumier D, Haine D, Garceau D, et al.Tramiprosate in mild-to-moderate Alzheimer's disease - a randomized, double-blind, placebo-controlled, multi-centre study (the Alphase Study). Arch Med Sci. 2011;7:102-11.
18. Molloy DW, Standish TI, Zhou Q, Guyatt G. A multicenter, blinded, randomized, factorial controlled trial of doxycycline and rifampin for treatment of Alzheimer's disease: the DARAD trial. Int J Geriatr Psychiatry. 2013;28:463-70.
19. Schneider L, Porsteinsson A, Farlow M, Shimakura A, Nakagawa M, Iwakami N. The neuroprotective and neurotrophic agent T-817MA for Alzheimer's disease: Randomized, double-blind, placebo-controlled proof-of-concept trial outcomes. Alzheimers Dement. 2013;9:530-1.
20. Soininen H, West C, Robbins J, Niculescu L: Long-term efficacy and safety of celecoxib in Alzheimer's disease. Dement Geriatr Cogn Disord. 2007;23:8-21.
21. Alzheimer disease: phase 2 trial results reported by Immune Network Ltd. The Free Library. 2002. http://www.thefreelibrary.com/Alzheimer%20Disease:%20Phase%202%20Trial%20Results%20Reported%20by%20Immune%20Network...-a092852880. Accessed 22 Sep 2015.
22. Scharf S, Mander A, Ugoni A, Vajda F, Christophidis N. A double-blind, placebo-controlled trial of diclofenac/misoprostol in Alzheimer's disease. Neurology. 1999;53:197-1.
23. Quinn JF, Raman R, Thomas RG, Yurko-Mauro K, Nelson EB, van DC, et al. Docosahexaenoic acid supplementation and cognitive decline in Alzheimer disease: a randomized trial. JAMA. 2010;304:1903-11.
24. Freund-Levi Y, Eriksdotter-Jonhagen M, Cederholm T, Basun H, Faxen-Irving G, Garlind A, et al. Omega-3 fatty acid treatment in 174 patients with mild to moderate Alzheimer disease: OmegAD study: a randomized double-blind trial. Arch Neurol. 2006;63:1402-8.
25. Lee DY, Kim KW, Jhoo JH, Ryu S, Choo IH, Seo EH, at al. A multicenter, randomized, placebo-controlled, double-blind clincial trial of escitalopram on its atrophy-delaying effect in Alzheimer's disease. Alzheimers Dement. 2012;8:603.
26. Van Gool WA, Weinstein HC, Scheltens P, Walstra GJ. Effect of hydroxychloroquine on progression of dementia in early Alzheimer's disease: an 18-month randomised, double-blind, placebo-controlled study. Lancet. 2001;358:455-460.
27. Pasqualetti P, Bonomini C, Dal FG, Paulon L, Sinforiani E, Marra C, et al. A randomized controlled study on effects of ibuprofen on cognitive progression of Alzheimer's disease. Aging Clin Exp Res. 2009;21:102-10.
28. de JD, Jansen R, Hoefnagels W, Jellesma-Eggenkamp M, Verbeek M, Borm G, et al. No effect of one-year treatment with indomethacin on Alzheimer's disease progression: a randomized controlled trial. PLoS One. 2008;3:e1475.
29. Rogers J, Kirby LC, Hempelman SR, Berry DL, McGeer PL, Kaszniak AW, et al. Clinical trial of indomethacin in Alzheimer's disease. Neurology. 1993;43:1609-1611.
30. Piette F, Belmin J, Vincent H, Schmidt N, Pariel S, Verny M, et al. Masitinib as an adjunct therapy for mild-to-moderate Alzheimer's disease: a randomised, placebo-controlled phase 2 trial. Alzheimers Res Ther. 2011;3:16.
31. Aisen PS, Davis KL, Berg JD, Schafer K, Campbell K, Thomas RG, et al. A randomized controlled trial of prednisone in Alzheimer's disease. Alzheimer's Disease Cooperative Study. Neurology. 2000;54:588-593.
32. Turner RS, Thomas RG, Craft S, van Dyck CH, Mintzer J, Reynolds BA, et al. A randomized, double-blind, placebo-controlled trial of resveratrol for Alzheimer disease. Neurology. 2015. doi:10.1212/WNL.0000000000002035.
33. Reines SA, Block GA, Morris JC, Liu G, Nessly ML, Lines CR, et al. Rofecoxib: no effect on Alzheimer's disease in a 1-year, randomized, blinded, controlled study. Neurology. 2004;62:66-71.
34. Aisen PS, Schafer KA, Grundman M, Pfeiffer E, Sano M, Davis KL, et al. Effects of rofecoxib or naproxen vs placebo on Alzheimer disease progression: a randomized controlled trial. JAMA. 2003;289:2819-2826.
35. Cucinotta D, De Leo D, Frattola L, Trabucchi M, Albizatti M, Beltramelli A, et al. Dihydroergokryptine as long-term treatment of Alzheimer type dementia: a multicenter two-year follow-up. Arch Gerontol Geriatr. 1998;Suppl 6:103-10.
36. Remington R, Bechtel C, Larsen D, Samar A, Doshanjh L, Fishman P, et al. A Phase II Randomized Clinical Trial of a Nutritional Formulation for Cognition and Mood in Alzheimer's Disease. J Alzheimers Dis. 2015;45:395-405.
37. Filip V, Kolibas E. Selegiline in the treatment of Alzheimer's disease: a long-term randomized placebo-controlled trial. Czech and Slovak Senile Dementia of Alzheimer Type Study Group. J Psychiatry Neurosci. 1999;24:234-43.
38. Freedman M, Rewilak D, Xerri T, Cohen S, Gordon AS, Shandling M, et al. L-deprenyl in Alzheimer's disease: cognitive and behavioral effects. Neurology. 1998;50:660-668.
39. Koivisto K, Helkala E-L, Hanninen T, Vanhanen M, Aaltonen H, Reinikainen K, et al. Three-year follow-up of long-term selegiline treatment of Alzheimer's disease. J Neurol. 1995;242:S34-S35.
40. Burke WJ, Roccaforte WH, Wengel SP, Bayer BL, Ranno AE, Willcockson NK. L-deprenyl in the treatment of mild dementia of the Alzheimer type: results of a 15-month trial. J Am Geriatr Soc. 1993;41:1219-25.
41. Sano M, Ernesto C, Thomas RG, Klauber MR, Schafer K, Grundman M, et al. A controlled trial of selegiline, alpha-tocopherol, or both as treatment for Alzheimer's disease. The Alzheimer's Disease Cooperative Study. New Engl J Med. 1997;336:1216-1222.
42. Tariot PN, Schneider LS, Cummings J, Thomas RG, Raman R, Jakimovich LJ, et al. Alzheimer's Disease Cooperative Study Group. Chronic divalproex sodium to attenuate agitation and clinical progression of Alzheimer disease. Arch Gen Psychiatry 2011;68:853-61.
43. Wischik CM, Staff RT, Wischik DJ, Bentham P, Murray AD, Storey JM, et al. Tau aggregation inhibitor therapy: an exploratory phase 2 study in mild or moderate Alzheimer's disease. J Alzheimers Dis. 2015;44:705-20.
44. Krishnan KR, Charles HC, Doraiswamy PM, Mintzer J, Weisler R, Yu X, et al. Randomized, placebo-controlled trial of the effects of donepezil on neuronal markers and hippocampal volumes in Alzheimer's disease. Am J Psychiatry. 2003;160:2003-11.
45. Burns A, Rossor M, Hecker J, Gauthier S, Petit H, Moller HJ, et al. The effects of donepezil in Alzheimer's disease - results from a multinational trial. Dement Geriatr Cogn Disord. 1999;10:237-44.
46. Rogers SL, Farlow MR, Doody RS, Mohs R, Friedhoff LT. A 24-week, double-blind, placebo-controlled trial of donepezil in patients with Alzheimer's disease. Donepezil Study Group. Neurology. 1998;50:136-45.
47. Raskind MA, Peskind ER, Wessel T, Yuan W. Galantamine in AD: A 6-month randomized, placebo-controlled trial with a 6-month extension. The Galantamine USA-1 Study Group. Neurology. 2000;54:2261-68.
48. A Phase 3 Study Evaluating Safety and Effectiveness of Immune Globulin Intravenous (IGIV 10%) for the Treatment of Mild-to-Moderate Alzheimer's Disease. ClinicalTrials.gov. 2015. http://www.clinicaltrials.gov/ct2/show/NCT00818662. Accessed 9 Oct 2015.
49. Relkin N. Results of the GAP 160701 study: A phase 3 clinical trial of intravenous immunoglobulin for mild-to-moderate Alzheimer's disease. Alzheimers Dement. 2013;9:530.
50. Safety and Efficacy Study Evaluating Dimebon in Patients With Mild to Moderate Alzheimer's Disease on Donepezil (CONCERT). ClinicalTrials.gov. 2012. http://www.clinicaltrials.gov/ct2/show/NCT00829374. Accessed 9 Oct 2015.
51. Sweetlove M. Phase III CONCERT Trial of Latrepirdine. Pharm Med. 2012;26:113-5.
52. A Safety and Efficacy Study of Oral Dimebon in Patients With Mild-To-Moderate Alzheimer's Disease (CONNECTION). ClinicalTrials.gov. 2012. http://www.clinicaltrials.gov/ct2/show/NCT00675623. Accessed 9 Oct 2015.
53. Pfizer And Medivation Announce Results From Two Phase 3 Studies In Dimebon (latrepirdine*) Alzheimer's Disease Clinical Development Program. Pfizer. 2010. http://press.pfizer.com/press-release/pfizer-and-medivation-announce-results-two-phase-3-studies-dimebon-latrepirdine-alzhei. Accessed 12 Oct 2015.
54. Doody RS, Gavrilova SI, Sano M, Thomas RG, Aisen PS, Bachurin SO, et al. Effect of dimebon on cognition, activities of daily living, behaviour, and global function in patients with mild-to-moderate Alzheimer's disease: a randomised, double-blind, placebo-controlled study. Lancet. 2008;372:207-15.
55. Alvarez XA, Cacabelos R, Laredo M, Couceiro V, Sampedro C, Varela M, et al. A 24-week, double-blind, placebo-controlled study of three dosages of Cerebrolysin in patients with mild to moderate Alzheimer's disease. Eur J Neurol. 2006;13:43-54.
56. Wang T, Huang Q, Reiman EM, Chen K, Li X, Li G, et al. Effects of memantine on clinical ratings, fluorodeoxyglucose positron emission tomography measurements, and cerebrospinal fluid assays in patients with moderate to severe Alzheimer dementia: a 24-week, randomized, clinical trial. J Clin Psychopharmacol. 2013;33:636-42.
57. Wilkinson D, Fox NC, Barkhof F, Phul R, Lemming O, Scheltens P. Memantine and brain atrophy in Alzheimer's disease: a 1-year randomized controlled trial. J Alzheimers Dis. 2012;29:459-69.
58. Ashford JW, Adamson M, Beale T, La D, Hernandez B, Noda A, et al. MR spectroscopy for assessment of memantine treatment in mild to moderate Alzheimer dementia. J Alzheimers Dis. 2011;26 Suppl 3:331-6.
59. Modrego PJ, Fayed N, Errea JM, Rios C, Pina MA, Sarasa M. Memantine versus donepezil in mild to moderate Alzheimer's disease: A randomized trial with magnetic resonance spectroscopy. Eur J Neurol. 2010;17:405-12.
60. Schmidt R, Ropele S, Pendl B, Ofner P, Enzinger C, Schmidt H, et al. Longitudinal multimodal imaging in mild to moderate Alzheimer disease: a pilot study with memantine. J Neurol Neurosurg Psychiatry. 2008;79:1312-7.
61. Gold M, Alderton C, Zvartau-Hind M, Egginton S, Saunders AM, Irizarry M, et al. Rosiglitazone monotherapy in mild-to-moderate Alzheimer's disease: results from a randomized, double-blind, placebo-controlled phase III study. Dement Geriatr Cogn Disord. 2010;30:131-46.
62. Risner ME, Saunders AM, Altman JF, Ormandy GC, Craft S, Foley IM, et al. Efficacy of rosiglitazone in a genetically defined population with mild-to-moderate Alzheimer's disease. Pharmacogenomics J. 2006;6:246-54.
63. Galasko D, Bell J, Mancuso JY, Kupiec JW, Sabbagh MN, van DC, et al. Clinical trial of an inhibitor of RAGE-Abeta interactions in Alzheimer disease. Neurology. 2014;82:1536-42.
64. Mohs RC, Knopman D, Petersen RC, Ferris SH, Ernesto C, Grundman M, et al. Development of cognitive instruments for use in clinical trials of antidementia drugs: additions to the Alzheimer's Disease Assessment Scale that broaden its scope. The Alzheimer's Disease Cooperative Study. Alzheimer Dis Assoc Disord. 1997;11:S13-S21.
65. Morris JC. The Clinical Dementia Rating (CDR): current version and scoring rules. Neurology. 1993;43:2412-4.
66. Folstein MF, Folstein SE, McHugh PR. "Mini-mental state". A practical method for grading the cognitive state of patients for the clinician. J Psychiatr Res. 1975;12:189-98.
