# Supplementary material for: A review of clinical trial designs used to detect a disease-modifying effect of drug therapy in Alzheimer’s disease and Parkinson’s disease
Source: BMC Neurol. 2016 Jun 16;16:92. doi: 10.1186/s12883-016-0606-3 (PMC4910262; doi:10.1186/s12883-016-0606-3)
Supplement: Additional file 4: — Baseline characteristics of participants in completed PD RCTs. (DOCX 42 kb) [file 12883_2016_606_MOESM4_ESM.docx]

**Additional file 4: Baseline characteristics of participants in completed randomised controlled trials of putative disease-modifying agents in Parkinson’s disease**

| **Trial** | **Year published** | **Location** | **Active agent(s)** | **Putative mechanism(s)** | **Number of participants randomised** | **Characteristics of participants at baseline** | | | | | |
| --- | --- | --- | --- | --- | --- | --- | --- | --- | --- | --- | --- |
|  |  |  |  |  |  | **Age** | **H&Y** | **Total UPDRS** | **UPDRS (III)** | **% on symptomatic treatment** | |
| PRECEPT [1] | 2007 | North America | CEP-1347 (3 dosages) | Anti-apoptotic | 806 | 60 | 1.8 | 25 | 18 | 0% | (n=0) |
| Green tea [2, 3] | 2009 † | China | Green tea polyphenols (3 dosages) | Anti-apoptotic | 410 | - | - | - | - | 0% | (n=0) |
| TCH346 [4] | 2006 | International | TCH346 (3 dosages) | Anti-apoptotic | 301 | 62 | 1.4 | 22 ‡ | - | 0% | (n=0) |
| Exenatide [5] | 2013 | UK | Exenatide | Anti-inflammatory and  promotes neurogenesis | 45 | 60 | 2.2 | 55 | 24 | 100% | (n=45) |
| H_2_-water [6] | 2013 | Japan | H_2_- dissolved water | Antioxidant | 18 | 63 | 2.1 | 17 | 13 | 100% | (n=18) |
| ROADS [7] | 1996 | North America | Lazabemide (4 dosages) | Antioxidant | 321 | 64 | - | 21 | 15 | 0% | (n=0) |
| Subtherapeutic pergolide [8] | 2004 | UK | Pergolide | Antioxidant | 106 | 61 | 1.6 | 25 ‡ | - | 0% | (n=0) |
| CALM-PD-CIT [9] | 2002 | North America | Pramipexole | Antioxidant | 82 | 61 | - | 33 | 22 | 0% | (n=0) |
| Rasagiline cognition [10] | 2015 • | USA | Rasagiline | Antioxidant | 50 | - | - | - | - | 100% | (n=50) |
| Russian rasagiline [11] | 2012 § | Russia | Rasagiline | Antioxidant | 40 | - | 1.0 | - | - | 0% | (n=0) |
| ADAGIO [12] | 2009 | International | Rasagiline (2 dosages) | Antioxidant | 1176 | 62 | 1.5 | 20 | 14 | 0% | (n=0) |
| TEMPO [13] | 2004 | North America | Rasagiline (2 dosages) | Antioxidant | 404 | 60 | 1.8 | 24 | 17 | 0% | (n=0) |
| REAL-PET [14] | 2003 | International | Ropinirole | Antioxidant | 186 | 60 | 1.8 | - | 19 | 0% | (n=0) |
| UK/France FDOPA PET [15] | 2002 | UK/France | Ropinirole | Antioxidant | 45 | 61 | 1.8 | - | 13 | 0% | (n=0) |
| Norwegian-Danish [16] | 1999 | Scandinavia | Selegiline | Antioxidant | 163 | 64 | 2.0 | 36 | 25 | 34% | (n=54) |
| SELEDO [17] | 1999 | European | Selegiline | Antioxidant | 116 | 61 | 2.1 | - | - | 33% | (n=36/109) |
| Swedish selegiline [18] | 1998 | Sweden | Selegiline | Antioxidant | 157 | 64 | 1.4 | 22 | 16 | 0% | (n=0) |
| SINDEPAR [19] | 1995 | USA | Selegiline | Antioxidant | 101 | 66 | - | 22 | 13 | 0% | (n=0) |
| Finnish selegiline [20] | 1992 | Finland | Selegiline | Antioxidant | 54 | 61 | 1.4 | - | - | 0% | (n=0) |
| Tetrud and Langston [21] | 1989 | USA | Selegiline | Antioxidant | 54 | 61 | 1.5 | - | 22 | 0% | (n=0) |
| DATATOP [22] | 1993 | North America | Selegiline and α-tocopherol | Antioxidant | 800 | 61 | 1.7 | 25 | 17 | 0% | (n=0) |
| QE3 [23] | 2014 | North America | Coenzyme Q_10_ (2 dosages) | Antioxidant and mitochondrial stabiliser | 600 | 63 | 1.6 | 23 | 16 | 0% | (n=0) |
| QE2 [24] | 2002 | USA | Coenzyme Q_10_ (3 dosages) | Antioxidant and  mitochondrial stabiliser | 80 | 61 | 1.8 | 23 | 17 | 0% | (n=0) |
| Creatine-CoQ10 [25] | 2015 | China | Coenzyme Q_10_ and Creatine | Antioxidant and mitochondrial stabiliser | 75 | 63 | - | - | 18 | 100% | (n=75) |
| NET-PD LS-1 Creatine [26] | 2015 | North America | Creatine | Antioxidant and mitochondrial stabiliser | 1741 | 62 | - | 26 | 18 | Unclear – some were treated at baseline | |

| **Trial** | **Year published** | **Location** | **Active agent(s)** | **Putative mechanism(s)** | **Number of participants randomised** | **Characteristics of participants at baseline** | | | | | |
| --- | --- | --- | --- | --- | --- | --- | --- | --- | --- | --- | --- |
|  |  |  |  |  |  | **Age** | **H&Y** | **Total UPDRS** | **UPDRS (III)** | **% on symptomatic treatment** | |
| German Creatine [27] | 2006 | Germany | Creatine | Antioxidant and mitochondrial stabiliser | 60 | 60 | 1.5 | 27 | 17 | Unclear - some were treated at baseline. | |
| Fenugreek [28] | 2014 | India | IBHB (extract of *Trigonella foenum-graecum*, L. seeds) | Antioxidant and mitochondrial stabiliser | 50 | 61 | 1.6 | 41 | 28 | 100% | (n=50) |
| Ubiquinol-10 [29] | 2015 | Japan | Ubiquinol-10 | Antioxidant and mitochondrial stabiliser | 64 | 63 | 1.9 | 11 | 8 | 91% | (n=58) |
| FAIRPARK [30] | 2014 | France | Deferiprone | Antioxidant via iron chelation | 40 | 61 | 2.0 | - | 23.9 | 100% | (n=40) |
| ELLDOPA [31] | 2004 | North America | Levodopa (3 dosages) | Dopaminergic | 361 | 65 | 1.9 | 28 | 19 | 0% | (n=0) |
| PROUD [32] | 2013 | International | Pramipexole | Dopaminergic | 535 | 63 | 1.5 | 25 | 17 | 0% | (n=0) |
| GM1 ganglioside [33] | 2013 | USA | GM1 ganglioside | Influences lysosomal integrity & intracellular calcium homeostasis | 77 | 59 | - | 29 | 19 | ≥65% | (n≥50) |
| MitoQ trial [34] | 2010 | Australasia | Mitoquinone (2 dosages) | Mitochondrial-targeted antioxidant | 130 | - | - | 26 | - | 0% | (n=0) |
| Riluzole international [35] | 2002 § | International | Riluzole (2 dosages) | NMDA antagonist | 1084 | 63 | - | - | - | 0% | (n=0) |
| Riluzole USA [36] | 2002 | USA | Riluzole | NMDA antagonist | 20 | 62 | 1.2 | 31 | 20 | 50% | (n=10) |
| GPI-1485 (6 month trial) [37] | 2002 † | - | GPI-1485 (2 dosages) | Trophic factor | 300 | - | - | - | - | - | - |

(H&Y, Hoehn and Yahr staging scale [38]; NMDA, N-metyl-D-aspartate; Total UPDRS, total score derived from the Unified Parkinson’s Disease Rating Scale [39]; UPDRS (III), motor component of the Unified Parkinson’s Disease Rating Scale)

† Results available online as a press release, but not yet published as a paper.

‡ Only the combined scores of the UPDRS (III) and UPDRS (II) were given. The UPDRS (I) was not reported, but only adds about one point at this stage of the disease.

§ Published as a conference abstract, but not yet published as a paper.

• Results available on ClinicalTrials.gov, but not yet published as a paper.

**References**

1. Parkinson Study Group. Mixed lineage kinase inhibitor CEP-1347 fails to delay disability in early Parkinson disease. Neurology. 2007;69:1480-90.
2. Efficacy and safety of green tea polyphenol in de novo Parkinson's disease patients. ClinicalTrials.gov. 2011. http://www.clinicaltrials.gov/ct2/show/NCT00461942. Accessed 22 Sep 2015.
3. Ability to Slow Disease Progression and Safety and Tolerability of Green Tea Polyphenols in Early Parkinson's Disease. The Michael J.Fox Foundation for Parkinson's Research. 2013. https://www.michaeljfox.org/foundation/grant-detail.php?grant_id=187. Accessed 22 Sep 2015.
4. Olanow CW, Schapira AH, Lewitt PA, Kieburtz K, Sauer D, Olivieri G, et al. TCH346 as a neuroprotective drug in Parkinson's disease: a double-blind, randomised, controlled trial. Lancet Neurol. 2006;5:1013-20.
5. Aviles-Olmos I, Dickson J, Kefalopoulou Z, Djamshidian A, Ell P, Soderlund T, et al. Exenatide and the treatment of patients with Parkinson's disease. J Clin Invest. 2013;123:2730-6.
6. Yoritaka A, Takanashi M, Hirayama M, Nakahara T, Ohta S, Hattori N. Pilot study of H_2_ therapy in Parkinson's disease: A randomized double-blind placebo-controlled trial. Mov Disord. 2013;28:836-9.
7. The Parkinson Study Group. Effect of lazabemide on the progression of disability in early Parkinson's disease. Ann Neurol. 1996;40:99-107.
8. Grosset K, Grosset D, Lees A, Parkinson's Disease Research Group of the United Kingdom. Trial of subtherapeutic pergolide in de novo Parkinson's disease. Mov Disord. 2005;20:363-6.
9. Parkinson Study Group. Dopamine transporter brain imaging to assess the effects of pramipexole vs levodopa on Parkinson disease progression. JAMA. 2002;287:1653-61.
10. The Effect of Rasagiline on Cognition in Parkinson's Disease. ClinicalTrials.gov. 2015. http://www.clinIcaltrials.gov/ct2/show/NCT01382342. Accessed 9 Oct 2015.
11. Illarioshkin S, Karabanov A, Mirkasimov A, Verejutina I. Rasagiline in drug-nave Russian patients with early Parkinson's disease. Mov Disord. 2012;27:380.
12. Olanow CW, Rascol O, Hauser R, Feigin PD, Jankovic J, Lang A, et al. A double-blind, delayed-start trial of rasagiline in Parkinson's disease. N Eng J Med. 2009;361:1268-78.
13. Parkinson Study Group. A controlled, randomized, delayed-start study of rasagiline in early Parkinson disease. Arch Neurol. 2004;61:561-6.
14. Whone AL, Watts RL, Stoessl AJ, Davis M, Reske S, Nahmias C, et al. Slower progression of Parkinson's disease with ropinirole versus levodopa: The REAL-PET study. Ann Neurol. 2003;54:93-101.
15. Rakshi JS, Pavese N, Uema T, Ito K, Morrish PK, Bailey DL, et al. A comparison of the progression of early Parkinson's disease in patients started on ropinirole or L-dopa: an 18F-dopa PET study. J Neural Transm. 2002;109:1433-43.
16. Larsen JP, Boas J, Erdal JE. Does selegiline modify the progression of early Parkinson's disease? Results from a five-year study. The Norwegian-Danish Study Group. Eur J Neurol. 1999;6:539-547.
17. Przuntek H, Conrad B, Dichgans J, Kraus PH, Krauseneck P, Pergande G, et al. SELEDO: a 5-year long-term trial on the effect of selegiline in early Parkinsonian patients treated with levodopa. Eur J Neurol. 1999;6:141-150.
18. Palhagen S, Heinonen EH, Hagglund J, Kaugesaar T, Kontants H, Maki-Ikola O, et al. Selegiline delays the onset of disability in de novo parkinsonian patients. Swedish Parkinson Study Group. Neurology 1998;51:520-5.
19. Olanow CW, Hauser RA, Gauger L, Malapira T, Koller W, Hubble J, et al. The effect of deprenyl and levodopa on the progression of Parkinson's disease. Ann Neurol. 1995;38:771-7.
20. Myllyla VV, Sotaniemi KA, Vuorinen JA, Heinonen EH. Selegiline as initial treatment in de novo parkinsonian patients. Neurology. 1992;42:339-43.
21. Tetrud JW, Langston JW. The effect of deprenyl (selegiline) on the natural history of Parkinson's disease. Science. 1989;245:519-22.
22. The Parkinson Study Group. Effects of tocopherol and deprenyl on the progression of disability in early Parkinson's disease. N Eng J Med. 1993;328:176-83.
23. Beal MF, Oakes D, Shoulson I, Henchcliffe C, Galpern WR, Haas R, et al. A randomized clinical trial of high-dosage coenzyme Q10 in early Parkinson disease: no evidence of benefit. JAMA Neurol. 2014;71:543-52.
24. Shults CW, Oakes D, Kieburtz K, Beal MF, Haas R, Plumb S, et al. Effects of coenzyme Q10 in early Parkinson disease: evidence of slowing of the functional decline. Arch Neurol. 2002;59:1541-50.
25. Li Z, Wang P, Yu Z, Cong Y, Sun H, Zhang J, et al. The effect of creatine and coenzyme q10 combination therapy on mild cognitive impairment in Parkinson's disease. Eur Neurol. 2015;73:205-211.
26. Kieburtz K, Tilley BC, Elm JJ, Babcock D, Hauser R, Ross GW, et al. Effect of creatine monohydrate on clinical progression in patients with Parkinson disease: a randomized clinical trial. JAMA. 2015;313:584-93.
27. Bender A, Koch W, Elstner M, Schombacher Y, Bender J, Moeschl M, et al. Creatine supplementation in Parkinson disease: a placebo-controlled randomized pilot trial. Neurology. 2006;67:1262-4.
28. Nathan J, Panjwani S, Mohan V, Joshi V, Thakurdesai PA. Efficacy and safety of standardized extract of Trigonella foenum-graecum L seeds as an adjuvant to L-Dopa in the management of patients with Parkinson's disease. Phytother Res. 2014;28:172-8.
29. Yoritaka A, Kawajiri S, Yamamoto Y, Nakahara T, Ando M, Hashimoto K, et al. Randomized, double-blind, placebo-controlled pilot trial of reduced coenzyme Q10 for Parkinson's disease. Parkinsonism Relat Disord. 2015;21:911-6.
30. Devos D, Moreau C, Devedjian JC, Kluza J, Petrault M, Laloux C, et al. Targeting chelatable iron as a therapeutic modality in Parkinson's disease. Antioxid Redox Signal. 2014;21:195-210.
31. Fahn S, Oakes D, Shoulson I, Kieburtz K, Rudolph A, Lang A, et al. Levodopa and the progression of Parkinson's disease. N Eng J Med. 2004;351:2498-508.
32. Schapira AH, McDermott MP, Barone P, Comella CL, Albrecht S, Hsu HH, et al. Pramipexole in patients with early Parkinson's disease (PROUD): a randomised delayed-start trial. Lancet Neurol. 2013;12:747-55.
33. Schneider JS, Gollomp SM, Sendek S, Colcher A, Cambi F, Du W. A randomized, controlled, delayed start trial of GM1 ganglioside in treated Parkinson's disease patients. J Neurol Sci. 2013;324:140-8.
34. Snow BJ, Rolfe FL, Lockhart MM, Frampton CM, O'Sullivan JD, Fung V, et al. A double-blind, placebo-controlled study to assess the mitochondria-targeted antioxidant MitoQ as a disease-modifying therapy in Parkinson's disease. Mov Disord. 2010;25:1670-4.
35. Rascol O, Olanow W, Brooks D, Koch P, Truffinet R, Bejuit R. A 2-year, multicenter, placebo-controlled, double-blind, parallel-group study of the effect of riluzole on Parkinson's disease progression. Mov Disord. 2002;17:S39.
36. Jankovic J, Hunter C. A double-blind, placebo-controlled and longitudinal study of riluzole in early Parkinson's disease. Parkinsonism Relat Disord. 2002;8:271-6.
37. Guilford Pharmaceuticals Inc: Final phase II GPI 1485 (NIL-A) imaging data presented at the annual meeting of the American Academy of Neurology. PR Newswire. 2002. http://www.prnewswire.co.uk/news-releases/final-phase-ii-gpi-1485-nil-a-imaging-data-presented-at-the-annual-meeting-of-the-american-academy-of-neurology-155593265.html. Accessed 22 Sep 2015.
38. Goetz CG, Poewe W, Rascol O, Sampaio C, Stebbins GT, Counsell C, et al. Movement Disorder Society Task Force report on the Hoehn and Yahr staging scale: status and recommendations. Mov Disord. 2004;19:1020-8.
39. Fahn S, Eton RL, UPDRS Development Committee. The Unified Parkinson's Disease Rating Scale. In Recent Developments in Parkinson's Disease. Edited by Fahn S, Marsden CD, Calne D, et al. Florham Park, New Jersey: Macmillan Healthcare Information; 1987. p153-63.
