# Supplementary material for: A review of clinical trial designs used to detect a disease-modifying effect of drug therapy in Alzheimer’s disease and Parkinson’s disease
Source: BMC Neurol. 2016 Jun 16;16:92. doi: 10.1186/s12883-016-0606-3 (PMC4910262; doi:10.1186/s12883-016-0606-3)
Supplement: Additional file 5: — Status and key design features of ongoing or unpublished AD RCTs. (DOCX 42 kb) [file 12883_2016_606_MOESM5_ESM.docx]

**Additional file 5: Status and key design features of ongoing or unpublished randomised controlled trials of putative disease-modifying agents in Alzheimer’s disease**

| **Trial** | **Trial status** | **Comment** | **Location** | **Active agent(s)** | **Putative mechanism** | **Number of participants randomised** | **Pre-defined trial length (months)** | **Primary outcome measures** | **Biomarkers or**  **time-to-event outcomes used as secondary outcome measures** | **Methods used to differentiate symptomatic from disease-modifying effects of the agent** |
| --- | --- | --- | --- | --- | --- | --- | --- | --- | --- | --- |
| Pfizer Bapineuzumab APOE ε4 carriers [1] | Terminated early in 2012 | Terminated early after two large phase 3 studies showed no benefit | North America | Bapineuzumab | Anti-amyloid | 1099 | 18 | ADAS-cog  DAD | CSF: phospho-tau  MRI: whole brain vol.  PiB PET | Long-term follow-up.  Imaging and CSF biomarkers. |
| Pfizer Bapineuzumab APOE ε4 non-carriers [2] | Terminated early in 2012 | Terminated early after two large phase 3 studies showed no benefit | North America | Bapineuzumab | Anti-amyloid | 835 | 18 | ADAS-cog  DAD | CSF: phospho-tau  MRI: whole brain vol.  PiB PET | Long-term follow-up.  Imaging and CSF biomarkers. |
| Carvedilol [3] | Ongoing, estimated completion Jan 2016 | - | USA | Carvedilol | Anti-amyloid | Aiming to recruit 50 | 6 | HVLT | CSF: Aβ | Long-term follow-up.  CSF biomarkers, |
| Marguerite RoAD [4] | Ongoing, estimated completion Mar 2019 | - | International | Gantenerumab | Anti-amyloid | Aiming to recruit 1000 | 24 | ADAS-cog  ADCS-ADL  AV-45 PET | CSF: Aβ42  CSF: phospho-tau  CSF: total tau  Volumetric MRI | Long-term follow-up.  Imaging and CSF biomarkers. |
| GENISTEÍNA_2 [5] | Ongoing, estimated completion Sep 2018 | - | Spain | Genistein | Anti-amyloid | Aiming to recruit 50 | 6 | CSF: Aβ | CSF: phospho-tau | CSF biomarkers. |
| NILVAD [6] | Ongoing, estimated completion Dec 2017 | - | European | Nilvadipine | Anti-amyloid | Aiming to recruit 200 | 18 | ADAS-cog | - | Long-term follow-up. |
| NIC5-15 [7] | Unpublished, completed 2013. | - | USA | Pinitol (NIC5-15) | Anti-amyloid | Aimed to recruit 40 | 6 | ADAS-cog | Plasma: Aβ | Long-term follow-up and blood biomarkers. |
| **Trial** | **Trial status** | **Comment** | **Location** | **Active agent(s)** | **Putative mechanism** | **Number of participants randomised** | **Pre-defined trial length (months)** | **Primary outcome measures** | **Biomarkers or**  **time-to-event outcomes used as secondary outcome measures** | **Methods used to differentiate symptomatic from disease-modifying effects of the agent** |
| PIT-ROAD [8] | Terminated early in 2012 | The reasons for early termination are not clear. | Japan | Pitavastatin | Anti-amyloid | 38 | 12 | ADAS-cog | - | Long-term follow-up. |
| EXPEDITION 3 [9] | Ongoing, estimated completion Oct 2018 | - | International | Solanezumab (LY2062430) | Anti-amyloid | Aiming to recruit 2100 | 18 | ADAS-cog  ADCS-ADL | AV-45 PET  CSF: Aβ  Plasma: Aβ  Volumetric MRI | Long-term follow-up.  Delayed-start extension study after 18 months.  Imaging, CSF and blood biomarkers. |
| CLASP [10] | Unpublished, completed 2007 | - | USA | Simvastatin | Anti-amyloid | Aimed to recruit 400 | 18 | ADAS-cog | - | Long-term follow-up. |
| Global efficacy tarenflurbil [11] | Terminated early in 2008 | Terminated early as Myriad decided to stop developing tarenflurbil | International | Tarenflurbil | Anti-amyloid | 841 | 18 | ADAS-cog  ADCS-ADL | - | Long-term follow-up. |
| European 3APS [12] | Terminated early in 2007. | Terminated after Alphase study reported negative results. [PRESS RELEASE REFERENCE] | European | Tramiprosate  (2 dosages) | Anti-amyloid | 966 | 18 | Unclear | Unclear | Long-term follow-up. |
| EPOCH [13] | Ongoing, estimated completion Jul 2019 | - | International | Verubecestat  MK-8931 | Anti-amyloid | Aiming to recruit 1960 | 18 | ADAS-cog  ADCS-ADL | CSF: phosphor-tau  CSF: total tau  MRI: hippocampal vol.  Vizamyl PET | Long-term follow-up.  Delayed-start extension study after 18 months.  Imaging and CSF biomarkers. |
| Masitinib (phase 3) [14] | Ongoing, estimated completion Dec 2016 | - | Spain | Masitinib | Anti-inflammatory | Aiming to recruit 396 | 6 | ADAS-cog  ADCS-ADL | - | Long-term follow-up. |
| **Trial** | **Trial status** | **Comment** | **Location** | **Active agent(s)** | **Putative mechanism** | **Number of participants randomised** | **Pre-defined trial length (months)** | **Primary outcome measures** | **Biomarkers or**  **time-to-event outcomes used as secondary outcome measures** | **Methods used to differentiate symptomatic from disease-modifying effects of the agent** |
| SUN-AK [15] | Unpublished, completed 2015 | - | Germany | Epigallocatechin-gallate | Antioxidant | 21 | 18 | ADAS-cog | MRI: whole brain vol.  Time to hospitalisation  Time to death related to Alzheimer’s disease | Long-term follow-up.  Imaging biomarker.  Time-to-event outcome. |
| MNEMOSYNE [16] | Unpublished, completed 2011 | - | Canada | Neptune krill oil  (NKO^TM^) | Antioxidant | 175 | 6 | NTB | - | Long-term follow-up. |
| Rasagiline Rescue [17] | Ongoing, estimated completion Dec 16 | - | USA | Rasagiline | Antioxidant | Aiming to recruit 50 | 6 | FDG PET | - | Imaging biomarker. |
| RGM [18] | Unpublished, completed 2011 | - | USA | Resveratrol, Glucose and malate | Antioxidant | 27 | 12 | ADAS-cog | - | Long-term follow-up. |
| AD-IDEA [19] | Unpublished, completed 2013 | - | France | Vitamin D | Antioxidant | Aiming to recruit 120 | 6 | ADAS-cog | - | Long-term follow-up. |
| TRx-237-005 [20] | Ongoing, estimated completion May 2016 | - | International | Methylthioninium | Anti-tau | Aiming to recruit 700 | 18 | ADAS-cog  ADCS-CGIC  FDG PET | CSF: unspecified  MRI: whole brain vol. | Long-term follow-up.  Imaging and CSF biomarkers. |
| TRx-237-015 [21] | Ongoing, estimated completion Feb 2016 | - | International | Methylthioninium | Anti-tau | Aiming to recruit 833 | 15 | ADAS-cog  ADCS-CGIC | CSF: unspecified  FDG PET  MRI: whole brain vol. | Long-term follow-up.  Imaging and CSF biomarkers. |
| AMBAR [22] | Ongoing, estimated completion Dec 2016 | - | USA/Spain | Plasmapheresis with 20% albumin and intravenous immunoglobulin (3 dosages of immunoglobulin - high, low and none) | Immune modulator | Aiming to recruit 350 | 14 | ADAS-cog  ADCS-ADL | CSF: Aβ40, Aβ42  CSF: phospho-tau  CSF: total tau  FDG PET  MRI: hippocampal vol.  MRI: posterior cingulate  Plasma: Aβ40, Aβ42 | Long-term follow-up.  Imaging, CSF and blood biomarkers. |
| **Trial** | **Trial status** | **Comment** | **Location** | **Active agent(s)** | **Putative mechanism** | **Number of participants randomised** | **Pre-defined trial length (months)** | **Primary outcome measures** | **Biomarkers or**  **time-to-event outcomes used as secondary outcome measures** | **Methods used to differentiate symptomatic from disease-modifying effects of the agent** |
| NOURISH AD [23] | Ongoing, estimated completion Jul 2017 | - | USA | AC-1204 | Mitochondrial stabiliser | Aiming to recruit 480 | 6 | ADAS-cog | - | Long-term follow-up. |
| Riluzole [24] | Ongoing, estimated completion Nov 2017 | - | USA | Riluzole | NMDA antagonist | Aiming to recruit 48 | 6 | MRS: NAA in the prefrontal cortex and hippocampus  FDG PET | MRS: glutamate levels | Imaging biomarkers. |
| STEADFAST [25] | Ongoing, estimated completion Mar 2018 | - | USA | Azeliragon (TTP488) | RAGE antagonist | Aiming to recruit 800 | 18 | ADAS-cog  CDR-SB | FDG PET  Plasma: Aβ  Volumetric MRI | Long-term follow up.  Imaging and CSF biomarkers. |

**Key**

**Clinical rating scales**

ADAS-cog Alzheimer’s Disease Assessment Scale – cognitive subscale [26] [No distinction made between different versions]

ADCS-ADL Alzheimer’s Disease Cooperative Study – Activities of Daily Living inventory [27]

ADCS-CGIC Alzheimer’s Disease Cooperative Study Clinical - Global Impression of Change [28]

CDR-SB The Washington University Clinical Dementia Rating Sum-of-Boxes score [29]

DAD Disability Assessment for Dementia [30]

HVLT Hopkin’s Verbal Learning Test [31]

NTB Neuropsychological Test Battery [32]

**Biomarker modalities Metabolites**

CSF Cerebrospinal Fluid NAA N-acetylaspartate

MRI Magnetic Resonance Imaging

MRS Magnetic Resonance Spectroscopy

PET Positron Emission Tomography

**Proteins Other**

Aβ Amyloid beta IVIG Intravenous immunoglobulin

NMDA N-methyl-D-aspartate

RAGE Receptor for Advanced Glycation Endpoints

vol. Volume

**PET ligands**

AV-45 (E)-4-(2-(6-(2-(2-(2-18F-fluoroethoxy)ethoxy)ethoxy)pyridin-3-yl)vinyl)-N-methyl benzenamine

FDG [^18^F]-2-fluoro-2-deoxyglucose

PiB [^11^C]Pittsburgh compound B

Vizamyl [18F]-flutemetamol

**References**

1. Study evaluating the safety and efficacy of bapineuzumab in Alzheimer disease patients. ClinicalTrials.gov. 2012. http://www.clinicaltrials.gov/ct2/show/nct00676143. Accessed 22 Sep 2015.
2. Study evaluating the efficacy and safety of bapineuzumab in Alzheimer disease patients. ClinicalTrials.gov. 2013. http://www.clinicaltrials.gov/ct2/show/NCT00667810. Accessed 22 Sep 2015.
3. Trial of Carvedilol in Alzheimer's Disease. ClinicalTrials.gov. 2015. http://www.clinicaltrials.gov/ct2/show/NCT01354444. Accessed 9 Oct 2015.
4. A Study of Gantenerumab in Patients With Mild Alzheimer Disease. ClinicalTrials.gov. 2015. http://www.clinicaltrials.gov/ct2/show/NCT02051608. Accessed 9 Oct 2015.
5. Genistein as a Possible Treatment for Alzheimer's Disease. (GENISTEÍNA_2). ClinicalTrials.gov. 2015. http://www.clinicaltrials.gov/ct2/show/NCT01982578. Accessed 9 Oct 2015.
6. A Phase III Trial of Nilvadipine to Treat Alzheimer's Disease (NILVAD). ClinicalTrials.gov. 2015. http://www.clinicaltrials.gov/ct2/show/NCT02017340. Accessed 9 Oct 2015.
7. A Single Site, Randomized, Double-blind, Placebo Controlled Trial of NIC5-15 in Subjects with Alzheimer's Disease. ClinicalTrials.gov. 2013. http://www.clinicaltrials.gov/ct2/show/NCT01928420. Accessed 9 Oct 2015.
8. The clinical study of Pitavastatin treatment for group of mild to moderate Alzheimer's disease (PIT-ROAD). ClinicalTrials.gov. 2012. http://www.clincialtrials.gov/CT2/show/NCT00548145. Accessed 22 Sep 2015.
9. Progress of Mild Alzheimer's Disease in Participants on Solanezumab Versus Placebo (EXPEDITION 3). ClinicalTrials.gov. 2015. http://www.clinicaltrials.gov/ct2/show/NCT01900665. Accessed 9 Oct 2015.
10. Cholesterol lowering agents to slow progression (CLASP) of Alzheimer's disease study. ClinicalTrials.gov. 2009. http://www.clinicaltrials.gov/ct2/show/NCT00053599. Accessed 22 Sep 2015.
11. Wilcock G, Black S, Balch A, Amato D, Beelen A, Schneider L, et al. Safety and efficacy of tarenflurbil in subjects with mild Alzheimer's disease: results from an 18-month international multi-centre phase 3 trial. Alzheimers Dement. 2009;54:86.
12. European Study of 3APS in Mild to Moderate Alzheimer's Disease Patients. ClinicalTrials.gov. 2007. http://www.clinicaltrials.gov/ct2/show/NCT00217763. Accessed 9 Oct 2015.
13. An Efficacy and Safety Trial of Verubecestat (MK-8931) in Mild to Moderate Alzheimer's Disease (P07738) (EPOCH). ClinicalTrials.gov. 2015. http://www.clinicaltrials.gov/ct2/show/NCT01739348. Accessed 9 Oct 2015.
14. A Phase 3 Study to Evaluate the Safety and Efficacy of Masitinib in Patients with Mild to Moderate Alzheime'rs Disease. ClinicalTrials.gov. 2013. http://www.clinicaltrials.gov/ct2/show/NCT01872598. Accessed 9 Oct 2015.
15. Sunphenon EGCg (Epigallocatechin-Gallate) in the early stage of Alzheimer's disease (SUN-AK). ClinicalTrials.gov. 2015. http://www.clinicaltrials.gov/ct2/show/NCT00951834. Accessed 22 Sep 2015.
16. Neptune Krill Oil (NKO^TM^) in Early Stage Alzheimer's Disease (MNEMOSYNE). ClinicalTrials.gov. 2011. http://www.clinicaltrials.gov/ct2/show/NCT00867828. Accessed 9 Oct 2015.
17. Rasagiline Rescue in Alzheimer's Disease Clinical Trial (R2). ClinicalTrials.gov. 2015. http://www.clinicaltrials.gov/ct2/show/NCT02359552. Accessed 9 Oct 2015.
18. Randomized trial of a nutritional supplement in Alzheimer's disease. ClinicalTrials.gov. 2012. http://www.clinicaltrials.gov/ct2/show/NCT00678431. Accessed 22 Sep 2015.
19. Annweiler C, Fantino B, Parot-Schinkel E, Thiery S, Gautier J, Beauchet O. Alzheimer's disease - input of vitamin D with mEmantine assay (AD-IDEA trial): Study protocol for a randomized controlled trial. Trials. 2011;12:230.
20. Safety and Efficacy Study Evaluating TRx0237 in Subjects with Mild Alzheimer's Disease. ClinicalTrials.gov. 2015. http://www.clinicaltrials.gov/ct2/show/NCT01689233. Accessed 9 Oct 2015.
21. Safety and Efficacy Study Evaluating TRx0237 in Subjects with Mild to Moderate Alzheimer's Disease. ClinicalTrials.gov. 2014. http://www.clinicaltrials.gov/ct2/show/NCT01689246. Accessed 9 Oct 2015.
22. A Study to Evaluate Albumin and Immunoglobulin in Alzheimer's Disease (AMBAR). ClinicalTrials.gov. 2015. http://www.clinicaltrials.gov/ct2/show/NCT01561053. Accessed 9 Oct 2015.
23. AC-1204 26-Week Long Term Efficacy Response Trial with Optional Open-label Ext (NOURISH AD). ClinicalTrials.gov. 2015. http://www.clinicaltrials.gov/ct2/show/NCT01741194. Accessed 9 Oct 2015.
24. Riluzole in mild Alzheimer’s disease. ClinicalTrials.gov. 2015. http://www.clinicaltrials.gov/ct2/show/NCT01703117. Accessed 22 Sep 2015.
25. Evaluation of the Efficacy and Safety of Azeliragon (TTP488) in Patients with Mild Alzheimer's Disease (STEADFAST). 2015. http://www.clinicaltrials.gov/ct2/show/NCT02080364. Accessed 9 Oct 2015.
26. Mohs RC, Knopman D, Petersen RC, Ferris SH, Ernesto C, Grundman M, et al. Development of cognitive instruments for use in clinical trials of antidementia drugs: additions to the Alzheimer's Disease Assessment Scale that broaden its scope. The Alzheimer's Disease Cooperative Study. Alzheimer Dis Assoc Disord. 1997;11:S13-S21.
27. Galasko D, Bennett D, Sano M, Ernesto C, Thomas R, Grundman M, et al. An inventory to assess activities of daily living for clinical trials in Alzheimer's disease. The Alzheimer's Disease Cooperative Study. Alzheimer Dis Assoc Disord. 1997;11:S33-S39.
28. Schneider LS, Olin JT, Doody RS, Clark CM, Morris JC, Reisberg B, et al. Validity and reliability of the Alzheimer's Disease Cooperative Study-Clinical Global Impression of Change. The Alzheimer's Disease Cooperative Study. Alzheimer Dis Assoc Disord. 1997;11:S22-S32.
29. Morris JC. The Clinical Dementia Rating (CDR): current version and scoring rules. Neurology. 1993; 43:2412-4
30. Gelinas I, Gauthier L, McIntyre M, Gauthier S. Development of a functional measure for persons with Alzheimer's disease: the disability assessment for dementia. Am J Occup Ther. 1999;53:471-81.
31. Shapiro AM, Benedict RH, Schretlen D, Brandt J. Construct and concurrent validity of the Hopkins Verbal Learning Test-revised. Clin Neuropsychol. 1999;13:348-58.
32. Harrison J, Minassian SL, Jenkins L, Black RS, Koller M, Grundman M. A neuropsychological test battery for use in Alzheimer disease clinical trials. Arch Neurol. 2007;64:1323-9.
