# Supplementary material for: A review of clinical trial designs used to detect a disease-modifying effect of drug therapy in Alzheimer’s disease and Parkinson’s disease
Source: BMC Neurol. 2016 Jun 16;16:92. doi: 10.1186/s12883-016-0606-3 (PMC4910262; doi:10.1186/s12883-016-0606-3)
Supplement: Additional file 6: — Status and key design features of ongoing or unpublished PD RCTs. (DOCX 33 kb) [file 12883_2016_606_MOESM6_ESM.docx]

**Additional file 6: Status and key design features of ongoing or unpublished randomised controlled trials of putative disease-modifying agents in Parkinson’s disease**

| **Trial** | **Trial status** | **Location** | **Active agent(s)** | **Putative mechanism** | **Number of participants randomised** | **Pre-defined trial length (months)** | **Primary outcome measures** | **Biomarkers or**  **time-to-event outcomes used as secondary outcome measures** | **Methods used to differentiate symptomatic from disease-modifying effects of the agent** |
| --- | --- | --- | --- | --- | --- | --- | --- | --- | --- |
| MIREILLE [1] | Unpublished, completed 2013 | France | Bee venom | Anti-inflammatory | 50 | 12 | UPDRS (III) | [^123^I]FP-CIT SPECT | Long-term follow-up.  The change in the UPDRS (III) in the ‘off state’ will be compared between groups to ‘evaluate the effect of disease progression’.  Imaging biomarker. |
| Exenatide-PD [2] | Ongoing, estimated completion June 2016 | UK | Exenatide | Anti-inflammatory and  promotes neurogenesis | 60 | 14 | UPDRS (III) | CSF: BDNF  [^123^I]FP-CIT SPECT | Analysis of primary outcomes after wash-out (12 weeks) period.  CSF and imaging biomarkers. |
| STEADY-PD [3] | Ongoing, estimated completion  March 2019 | North America | Isradipine | Calcium channel blocker | Aiming to recruit 336 | 36 | Total UPDRS | Time to dopaminergic treatment  Time to dopaminergic motor complications | Long-term follow-up.  Time-to-event outcome. |
| G-CSF [4] | Unpublished, unclear if completed | Taiwan | G-CSF  (2 dosages) | Haematopoietic  growth factor | Aiming to recruit 36 | 24 | UPDRS (III) | - | Long-term follow-up. |
| ZONIST [5] | Unpublished, unclear if completed | Iran | Zonisamide | Neuromodulator | Aiming to recruit 60 | 12 | Time to dopaminergic treatment | - | Time-to-event outcome. |
| NICOPARK2 [6] | Unpublished, completed 2013 | France | Transdermal nicotine | Nicotinic agonist | 40 | 50* | UPDRS (III)  [^123^I]FP-CIT SPECT | - | Analysis of primary outcomes after wash-out (5 weeks) period.  Imaging biomarker. |
| NIC-PD [7] | Ongoing, estimated completion January 2015 | Germany and USA | Transdermal nicotine | Nicotinic agonist | Aiming to recruit 160 | 14* | Total UPDRS | Time to dopaminergic treatment | Long-term follow-up.  Analysis of both primary and secondary outcomes after wash-out (8 weeks) period.  Time-to-event outcome. |
| GPI-1485  (2 year trial) [8] | Unpublished, completed 2006 | USA | GPI-1485 | Trophic factor | Aiming to recruit 200 | 24 | [^123^I]β-CIT: striatal uptake | [^123^I]β-CIT: putamen and caudate uptake | Imaging biomarker. |

**Key**

‘Pre-defined trial length’ refers to the length of the trial not including any washout period unless the pre-defined primary analyses related to the change in an outcome measure from baseline until the end of the washout period (these studies are marked with an asterisk (*).

**Clinical rating scales**

Total UPDRS Total score derived from the Unified Parkinson’s Disease Rating Scale [9]

UPDRS (III) Motor component of the Unified Parkinson’s Disease Rating Scale [9]

**Biomarker modalities Other**

CSF Cerebral Spinal Fluid BDNF Brain-Derived Neurotrophic Factor

SPECT Single-Photon Emission Computed Tomography G-CSF Granulocyte-Colony Stimulating Factor

**SPECT ligands**

[^123^I]FP-CIT [^123^I]-2β-carbomethoxy-3β-(4-iodophenyl)-N-(3-fluoropropyl)-N-tropane

[^123^I]β-CIT [^123^I]-2β-carbomethoxy-3β-(4-iodophenyl tropane

**References**

1. Bee venom for the treatment of Parkinson disease (MIREILLE). ClinicalTrials.gov. 2014. http://www.clinicaltrials.gov/ct2/show/NCT01341431. Accessed 22 Sep 2015.
2. Trial of Exenatide for Parkinson's Disease (EXENATIDE-PD). ClinicalTrials.gov. 2015. http://www.clinicaltrials.gov/ct2/show/NCT01971242. Accessed 22 Sep 2015.
3. Efficacy of Isradipine in Early Parkinson's Disease ClinicalTrials.gov. 2015. http://www.clinicaltrials.gov/ct2/show/NCT02168842. Accessed 22 Sep 2015.
4. Study of the neuro-protective effect of Granulocyte-colony Stimulating Factor on early stage Parkinson's disease. ClinicalTrials.gov. 2013. http://www.clinicaltrials.gov/ct2/show/NCT01227681. Accessed 22 Sep 2015.
5. Study of zonisamide in early Parkinson disease (ZONIST). ClinicalTrials.gov. 2013. http://www.clinicaltrials.gov/ct2/show/NCT01766128. Accessed 22 Sep 2015.
6. Efficacy of transdermal nicotine, on motor symptoms in advanced Parkinson's disease (NICOPARK2). ClinicalTrials.gov. 2013. http://www.clinicaltrials.gov/ct2/show/NCT00873392. Accessed 22 Sep 2015.
7. Disease-modifying Potential of Transdermal NICotine in Early Parkinson's Disease (NIC-PD). ClinicalTrials.gov. 2014. http://www.clinicaltrials.gov/ct2/show/NCT01560754. Accessed 22 Sep 2015.
8. 2 year study to evaluate the effects of GPI 1485 on [123I]b-CIT/SPECT scanning and clinical efficacy in patients with PD. ClinicalTrials.gov. 2008. http://www.clinicaltrials.gov/ct2/show/NCT00209508. Accessed 22 Sep 2015.
9. Fahn S, Eton RL, UPDRS Development Committee. The Unified Parkinson's Disease Rating Scale. In: Fahn S, Marsden CD, Calne D, et al, editors. Recent Developments in Parkinson's Disease. Florham Park, New Jersey: Macmillan Healthcare Information; 1987. p153-63.
