# Supplementary material for: A review of clinical trial designs used to detect a disease-modifying effect of drug therapy in Alzheimer’s disease and Parkinson’s disease
Source: BMC Neurol. 2016 Jun 16;16:92. doi: 10.1186/s12883-016-0606-3 (PMC4910262; doi:10.1186/s12883-016-0606-3)
Supplement: Additional file 7: — Methods used to differentiate symptomatic from disease-modifying drug effects in all RCTs in AD. (DOCX 57 kb) [file 12883_2016_606_MOESM7_ESM.docx]

**Additional file 7: Overview of methods used to differentiate symptomatic from disease-modifying effects of putative disease-modifying agents in all included randomised controlled trials in Alzheimer’s disease**

| **Trial** | **Wash-in analysis** | **Wash-out analysis** | **Delayed-start trial design** | **Long-term follow-up** | **Biomarkers (primary or secondary outcome measures)** | | | | | **Time-to-event outcomes** |
| --- | --- | --- | --- | --- | --- | --- | --- | --- | --- | --- |
|  |  |  |  |  | **Imaging** | **CSF** | **Blood** | **Urine** | **EEG** |  |
| Aβ immunisation [1] |  |  |  | 15 months¥ | Volumetric MRI | Aβ  Total tau |  |  |  |  |
| LEADe [2] |  | 8 weeks |  | 18 months† | Volumetric MRI |  |  |  |  |  |
| Bapineuzumab (phase 3) APOE ε4 carriers [3] |  |  |  | 18 months† | PiB PET  Volumetric MRI | Phospho-tau |  |  |  |  |
| Bapineuzumab (phase 3) APOE ε4 non-carriers [3] |  |  |  | 18 months† | PiB PET  Volumetric MRI | Phospho-tau |  |  |  |  |
| Bapineuzumab (phase 2) [4] |  |  |  | 18 months† | Volumetric MRI | Aβ42  Phospho-tau  Total tau |  |  |  |  |
| ABBY [5, 6] |  |  |  | 17 monthsФ | Volumetric MRI | Unspecified |  |  |  |  |
| BLAZE [6-8] |  |  |  | 17 monthsФ | AV-45 PET  FDG PET  Volumetric MRI | Unspecified |  |  |  |  |
| Scyllo-inositol [9] |  |  |  | 18 months† | MRS Volumetric MRI | Aβ40, Aβ42  Phospho-tau  Total tau |  |  |  |  |
| IDENTITY [10, 11] |  | 16 weeks | 18 month delay |  | AV-35 PET  FDG PET Volumetric MRI | Aβ42  Phospho-tau  Tau | Aβ |  |  |  |
| IDENTITY2 [12] |  | 16 weeks | 18 month delay |  | AV-35 PET  FDG PET Volumetric MRI | Aβ42  Phospho-tau  Total tau | Aβ |  |  |  |
| Simvastatin [13] |  |  |  |  |  | Aβ40, Aβ42 |  |  |  |  |
| EXPEDITION 1 [14] |  |  |  | 18 months† | AV-45  Volumetric MRI | Aβ40, Aβ42  Phospho-tau  Total tau | Aβ40  Aβ42 |  |  |  |
| EXPEDITION 2 [14] |  |  |  | 18 months† | AV-45  Volumetric MRI | Aβ40, Aβ42  Phospho-tau  Total tau | Aβ40  Aβ42 |  |  |  |
| Tarenflurbil (phase 3) [15] |  |  |  | 18 months‡ |  |  |  |  |  |  |
| Tarenflurbil (phase 2) [16] |  |  | 12 month delay | 24 months† |  |  |  |  |  |  |
| **Trial** | **Wash-in analysis** | **Wash-out analysis** | **Delayed-start trial design** | **Long-term follow-up** | **Biomarkers (primary or secondary outcome measures)** | | | | | **Time-to-event outcomes** |
|  |  |  |  |  | **Imaging** | **CSF** | **Blood** | **Urine** | **EEG** |  |
| Alphase [17] |  |  |  |  | Volumetric MRI | Aβ and tau | Aβ | Aβ |  |  |
| DARAD [18] |  |  |  | 12 months† |  |  |  |  |  |  |
| T-817MA [19] |  |  |  | 12 monthsФ | Volumetric MRI |  |  |  |  |  |
| Celecoxib [20] |  |  |  | 12 months¥ |  |  |  |  |  |  |
| DAD2000 [21] |  |  |  | 12 monthsФ |  |  |  |  |  |  |
| Diclofenac + misoprostol [22] |  |  |  | 6 months¥ |  |  |  |  |  |  |
| Docosahexaenoic acid [23] |  |  |  | 18 months† | Volumetric MRI |  |  |  |  |  |
| OmegAD [24] |  |  | 6 month delay | 12 months† |  |  |  |  |  |  |
| Escitalopram [25] |  |  |  |  | Volumetric MRI |  |  |  |  |  |
| Hydroxychloroquine [26] |  |  |  | 18 months† |  |  |  |  |  |  |
| Ibuprofen [27] |  |  |  | 12 months¥ |  |  |  |  |  |  |
| Dutch indomethacin [28] |  |  |  | 12 months¥ |  |  |  |  |  |  |
| American indomethacin [29] |  |  |  | 6 months¥ |  |  |  |  |  |  |
| Masitinib (phase 2) [30] |  |  |  | 6 months† |  |  |  |  |  |  |
| Prednisone [31] |  |  |  | 12 months¥ |  |  |  |  |  |  |
| Resveratrol [32] |  |  |  |  | Volumetric MRI | Aβ40, Aβ42  Phospho-tau  Total tau | Aβ40 Aβ42 |  |  |  |
| Rofecoxib [33] |  | 12 weeks |  | 12 months† |  |  |  |  |  |  |
| Rofecoxib or naproxen [34] |  | 8 weeks |  |  |  |  |  |  |  | Time to the following separate endpoints: (1) 4 point decline in ADAS-cog; (2) 1 step worsening on global CDR; (3) 15 point decline on ADCS-ADL; (4) institutionalisation; (5)death |
| DAV.I.D.E .[35] |  |  | 12 month delay | 24 months† |  |  |  |  |  |  |
| Nutritional formulation [36] |  |  | 3-6 month delay |  |  |  |  |  |  |  |
| Czech/Slovak selegiline [37] | 6 and 12 weeks |  |  | 6 months¥ |  |  |  |  | Dominant frequencies |  |
| Canadian selegiline [38] |  | 12 weeks |  |  |  |  |  |  |  |  |
| KUOSTAD [39] |  |  |  | 36 monthsФ |  |  |  |  |  |  |
| Nebraska selegiline [40] |  |  |  | 15 months¥ |  |  |  |  |  |  |
| Selegiline & tocopherol [41] |  |  |  | 24 months† |  |  |  |  |  | Time to either severe dementia (Global CDR = 3), loss of ability to perform basic ADLs, institutionalisation, or death |
| **Trial** | **Wash-in analysis** | **Wash-out analysis** | **Delayed-start trial design** | **Long-term follow-up** | **Biomarkers (primary or secondary outcome measures)** | | | | | **Time-to-event outcomes** |
|  |  |  |  |  | **Imaging** | **CSF** | **Blood** | **Urine** | **EEG** |  |
| VALID [42] |  | 8 weeks |  | 24 months† | Volumetric MRI |  |  |  |  | Time to clinically significant agitation or psychosis (defined as a score of ≥ 3 on ≥ 1 NPI items assessing delusions, hallucinations and agitation/aggression) |
| TauRx (phase 2) [43] |  |  |  | 6 months† | HMPAO SPECT |  |  |  |  |  |
| Donepezil MRI/MRS [44] |  | 6 weeks |  | 6 months† | MRS  Volumetric MRI |  |  |  |  |  |
| Donepezil international [45] |  | 6 weeks |  | 6 months† |  |  |  |  |  |  |
| Donepezil USA clinical [46] |  | 6 weeks |  | 6 months† |  |  |  |  |  |  |
| Galantamine [47] | 12 weeks |  | 6 month delay | 12 months† |  |  |  |  |  |  |
| GAP Study [48, 49] |  |  |  | 18 monthsФ | AV-45 PET  FDG PET | Aβ42  Phospho-tau  Total tau |  |  |  |  |
| CONCERT [50, 51] |  |  |  | 12 monthsФ |  |  |  |  |  |  |
| CONNECTION [52, 53] |  |  |  | 6 monthsФ |  |  |  |  |  |  |
| Russian Dimebon [54] |  |  |  | 6 months† |  |  |  |  |  |  |
| Cerebrolysin [55] | 4 weeks | 12 weeks |  | 6 months† |  |  |  |  |  |  |
| Memantine PET [56] |  |  |  |  | FDG PET | Aβ40, Aβ42  Phospho-tau  Total tau |  |  |  |  |
| Memantine MRI [57] |  |  |  |  | Volumetric MRI |  |  |  |  |  |
| Memantine MRS [58] |  |  |  |  | MRS |  |  |  |  |  |
| Memantine vs. donepezil MRS [59] |  |  |  |  | MRS |  |  |  |  |  |
| Memantine multimodal [60] |  |  |  |  | FDG PET  MRS  Volumetric MRI |  |  |  |  |  |
| REFLECT-1 [61] |  |  |  | 6 months¥ |  |  |  |  |  |  |
| Rosiglitazone genetics [62] |  |  |  | 6 months† |  |  |  |  |  |  |
| Azeliragon [63] |  |  |  | 18 months† | Volumetric MRI | Aβ1-x  Aβ40, Aβ42  Phospho-tau  Total tau |  |  |  |  |

| **Trial** | **Wash-in analysis** | **Wash-out analysis** | **Delayed-start trial design** | **Long-term follow-up** | **Biomarkers (primary or secondary outcome measures)** | | | | | **Time-to-event outcomes** |
| --- | --- | --- | --- | --- | --- | --- | --- | --- | --- | --- |
|  |  |  |  |  | **Imaging** | **CSF** | **Blood** | **Urine** | **EEG** |  |
| **Planned, Ongoing and**  **Unpublished RCTs** |  |  |  |  |  |  |  |  |  |  |
| Pfizer Bapineuzumab  APOE ε4 carriers [64] |  |  |  | 18 months | Volumetric MRI  PiB PET | Phospho-tau |  |  |  |  |
| Pfizer Bapineuzumab  APOE ε4 non-carriers [65] |  |  |  | 18 months | Volumetric MRI  PiB PET | Phospho-tau |  |  |  |  |
| Carvedilol [66] |  |  |  | 6 months |  | Aβ |  |  |  |  |
| Marguerite RoAD [67] |  |  |  | 24 months | AV-45 PET  Volumetric MRI | Aβ  Phospho-tau  Total tau |  |  |  |  |
| GENISTEÍNA_2 [68] |  |  |  |  |  | Aβ  Phospho-tau |  |  |  |  |
| NILVAD [69] |  |  |  | 18 months |  |  |  |  |  |  |
| NIC5-15 [70] |  |  |  | 6 months |  |  | Aβ |  |  |  |
| PIT-ROAD [71] |  |  |  | 12 months |  |  |  |  |  |  |
| EXPEDITION 3 [72] |  |  | 18 months  (extension study) | 18 months | AV-45 PET  Volumetric MRI | Aβ | Aβ |  |  |  |
| CLASP [73] |  |  |  | 18 months |  |  |  |  |  |  |
| Global efficacy tarenflurbil [74] |  |  |  | 18 months |  |  |  |  |  |  |
| European 3APS [75] |  |  |  | 18 months |  |  |  |  |  |  |
| EPOCH [76] |  |  | 18 months  (extension study) | 18 months | Volumetric MRI  Vizamyl PET | Phospho-tau  Total tau |  |  |  |  |
| Masitinib (phase 3) [77] |  |  |  | 6 months |  |  |  |  |  |  |
| SUN-AK [78] |  |  |  | 18 months | Volumetric MRI |  |  |  |  | Time to the following endpoints: (1) hospitalisation; (2) death related to Alzheimer’s disease |
| MNEMOSYNE [79] |  |  |  | 6 months |  |  |  |  |  |  |
| Rasagiline Rescue [80] |  |  |  |  | FDG PET |  |  |  |  |  |
| RGM [81] |  |  |  | 12 months |  |  |  |  |  |  |
| AD-IDEA [82] |  |  |  | 6 months |  |  |  |  |  |  |
| TRx-237-005 [83] |  |  |  | 18 months | FDG PET  Volumetric MRI | Unspecified |  |  |  |  |
| **Trial** | **Wash-in analysis** | **Wash-out analysis** | **Delayed-start trial design** | **Long-term follow-up** | **Biomarkers (primary or secondary outcome measures)** | | | | | **Time-to-event outcomes** |
|  |  |  |  |  | **Imaging** | **CSF** | **Blood** | **Urine** | **EEG** |  |
| TRx-237-015 [84] |  |  |  | 15 months | FDG PET Volumetric MRI | Unspecified |  |  |  |  |
| AMBAR [85] |  |  |  | 14 months | FDG PET  Volumetric MRI | Aβ40, Aβ42  Phospho-tau  Total tau | Aβ40  Aβ42 |  |  |  |
| NOURISH AD [86] |  |  |  | 6 months |  |  |  |  |  |  |
| Riluzole [87] |  |  |  |  | MRS  FDG PET |  |  |  |  |  |
| STEADFAST [88] |  |  |  | 18 months | FDG PET Volumetric MRI |  | Aβ |  |  |  |

**Key**

**Long-term follow-up studies (published studies only)**

**Biomarker modalities** † Image published (e.g. Kaplan Meier plot) from which the presence/absence of sustained

CSF Cerebrospinal fluid divergence in outcome measures could be inferred. No formal slope analyses conducted.

EEG Electroencephalography ‡ Formal slope analyses conducted to look for sustained divergence.

MRI Magnetic Resonance Imaging ¥ No formal slope analysis conducted nor image published from which sustained divergence

MRS Magnetic Resonance Spectroscopy in outcome measures between groups can be inferred. Furthermore, no alternative

PET Positron Emission Tomography strategy used to try to demonstrate disease-modification.

SPECT Single Photon Emission Computed Tomography Ф Insufficient information to classify (e.g. only published as conference abstract)

**Proteins Clinical rating scales**

Aβ Amyloid beta ADAS-cog Alzheimer’s Disease Assessment Scale – cognitive subscale [89]

Aβ40 Amyloid beta isomer, length 40 amino acids ADCS-ADL Alzheimer’s Disease Cooperative Study – Activities of Daily Living inventory [90]

Aβ42 Amyloid beta isomer, length 42 amino acids Global CDR The Washington University Clinical Dementia Rating global score [91]

NPI Neuropsychiatric Inventory [92]

**PET ligands**

AV-45 (E)-4-(2-(6-(2-(2-(2-18F-fluoroethoxy)ethoxy)ethoxy)pyridin-3-yl)vinyl)-N-methyl benzenamine

FDG [^18^F]-2-fluoro-2-deoxyglucose

PiB [^11^C]Pittsburgh compound B

Vizamyl [18F]-flutemetamol

**SPECT ligands**

HMPAO [^99m^Tc]-hexamethylpropylene amine oxidase

**References**

1. Gilman S, Koller M, Black RS, Jenkins L, Griffith SG, et al. Clinical effects of Abeta immunization (AN1792) in patients with AD in an interrupted trial. Neurology. 2005;64:1553-62.
2. Feldman HH, Doody RS, Kivipelto M, Sparks DL, Waters DD, Jones RW, et al. Randomized controlled trial of atorvastatin in mild to moderate Alzheimer disease: LEADe. Neurology. 2010;74:956-64.
3. Salloway S, Sperling R, Fox NC, Blennow K, Klunk W, Raskind M, et al. Two phase 3 trials of bapineuzumab in mild-to-moderate Alzheimer's disease. N Engl J Med. 2014;370:322-33.
4. Salloway S, Sperling R, Gilman S, Fox NC, Blennow K, Raskind M, et al. A phase 2 multiple ascending dose trial of bapineuzumab in mild to moderate Alzheimer disease. Neurology. 2009;73:2061-70.
5. A Study to Evaluate the Efficacy and Safety of MABT5102A in Patient With Mild to Moderate Alzheimer's Disease (ABBY). ClinicalTrials.gov. 2015. http://www.clinicaltrials.gov/ct2/show/NCT01343966. Accessed 9 Oct 2015.
6. Roche announces phase II clinical results of crenezumab in Alzheimers disease. Roche. 2014. http://www.roche.com/investors/updates/inv-update-2014-07-16.htm. Accessed 12 Oct 2015.
7. A Study to Evaluate the Impact of MABT5102A on Brain Amyloid Load and Related Biomarkers in Patients with Mild to Moderate Alzheimer's Disease. ClinicalTrials.gov. 2015. http://www.clinicaltrials.gov/ct2/show/NCT01397578. Accessed 9 Oct 2015.
8. AC Immune receives milestone payment for crenezumab moving into phase III clinical development in Alzheimer's disease. Swiss Biotech. 2015. http://www.swissbiotech.org/b/index.php?1=1&id=665679. Accessed 12 Oct 2015.
9. Salloway S, Sperling R, Keren R, Porsteinsson AP, van Dyck CH, Tariot PN, et al. A phase 2 randomized trial of ELND005, scyllo-inositol, in mild to moderate Alzheimer disease. Neurology. 2011;77:1253-62.
10. Effect of LY450139 on the Long Term Progression of Alzheimer's Disease. ClinicalTrials.gov. 2015. http://www.clinicaltrials.gov/ct2/show/NCT00594568. Accessed 9 Oct 2015.
11. Doody RS, Raman R, Farlow M, Iwatsubo T, Vellas B, Joffe S, et al. A phase 3 trial of semagacestat for treatment of Alzheimer's disease. N Engl J Med. 2013;369:341-50.
12. Effect of LY450139, on the progression of Alzheimer's disease as compared with placebo (IDENTITY-2). ClinicalTrials.gov. 2015. http://www.clinicaltrials.gov/ct2/show/NCT00762411. Accessed 22 Sep 2015.
13. Simons M, Schwarzler F, Lutjohann D, von BK, Beyreuther K, Dichgans J, et al. Treatment with simvastatin in normocholesterolemic patients with Alzheimer's disease: A 26-week randomized, placebo-controlled, double-blind trial. Ann Neurol. 2002;52:346-50.
14. Doody RS, Thomas RG, Farlow M, Iwatsubo T, Vellas B, Joffe S, et al. Phase 3 trials of solanezumab for mild-to-moderate Alzheimer's disease. N Engl J Med. 2014;370:311-21.
15. Green RC, Schneider LS, Amato DA, Beelen AP, Wilcock G, Swabb EA, et al. Effect of tarenflurbil on cognitive decline and activities of daily living in patients with mild Alzheimer disease: a randomized controlled trial. JAMA. 2009;302:2557-64.
16. Wilcock GK, Black SE, Hendrix SB, Zavitz KH, Swabb EA, Laughlin MA. Efficacy and safety of tarenflurbil in mild to moderate Alzheimer's disease: a randomised phase II trial. Lancet Neurol. 2008;7:483-93.
17. Aisen PS, Gauthier S, Ferris SH, Saumier D, Haine D, Garceau D, et al.Tramiprosate in mild-to-moderate Alzheimer's disease - a randomized, double-blind, placebo-controlled, multi-centre study (the Alphase Study). Arch Med Sci. 2011;7:102-11.
18. Molloy DW, Standish TI, Zhou Q, Guyatt G. A multicenter, blinded, randomized, factorial controlled trial of doxycycline and rifampin for treatment of Alzheimer's disease: the DARAD trial. Int J Geriatr Psychiatry. 2013;28:463-70.
19. Schneider L, Porsteinsson A, Farlow M, Shimakura A, Nakagawa M, Iwakami N. The neuroprotective and neurotrophic agent T-817MA for Alzheimer's disease: Randomized, double-blind, placebo-controlled proof-of-concept trial outcomes. Alzheimers Dement. 2013;9:530-1.
20. Soininen H, West C, Robbins J, Niculescu L: Long-term efficacy and safety of celecoxib in Alzheimer's disease. Dement Geriatr Cogn Disord. 2007;23:8-21.
21. Alzheimer disease: phase 2 trial results reported by Immune Network Ltd. The Free Library. 2002. http://www.thefreelibrary.com/Alzheimer%20Disease:%20Phase%202%20Trial%20Results%20Reported%20by%20Immune%20Network...-a092852880. Accessed 22 Sep 2015.
22. Scharf S, Mander A, Ugoni A, Vajda F, Christophidis N. A double-blind, placebo-controlled trial of diclofenac/misoprostol in Alzheimer's disease. Neurology. 1999;53:197-1.
23. Quinn JF, Raman R, Thomas RG, Yurko-Mauro K, Nelson EB, van DC, et al. Docosahexaenoic acid supplementation and cognitive decline in Alzheimer disease: a randomized trial. JAMA. 2010;304:1903-11.
24. Freund-Levi Y, Eriksdotter-Jonhagen M, Cederholm T, Basun H, Faxen-Irving G, Garlind A, et al. Omega-3 fatty acid treatment in 174 patients with mild to moderate Alzheimer disease: OmegAD study: a randomized double-blind trial. Arch Neurol. 2006;63:1402-8.
25. Lee DY, Kim KW, Jhoo JH, Ryu S, Choo IH, Seo EH, at al. A multicenter, randomized, placebo-controlled, double-blind clincial trial of escitalopram on its atrophy-delaying effect in Alzheimer's disease. Alzheimers Dement. 2012;8:603.
26. Van Gool WA, Weinstein HC, Scheltens P, Walstra GJ. Effect of hydroxychloroquine on progression of dementia in early Alzheimer's disease: an 18-month randomised, double-blind, placebo-controlled study. Lancet. 2001;358:455-460.
27. Pasqualetti P, Bonomini C, Dal FG, Paulon L, Sinforiani E, Marra C, et al. A randomized controlled study on effects of ibuprofen on cognitive progression of Alzheimer's disease. Aging Clin Exp Res. 2009;21:102-10.
28. de JD, Jansen R, Hoefnagels W, Jellesma-Eggenkamp M, Verbeek M, Borm G, et al. No effect of one-year treatment with indomethacin on Alzheimer's disease progression: a randomized controlled trial. PLoS One. 2008;3:e1475.
29. Rogers J, Kirby LC, Hempelman SR, Berry DL, McGeer PL, Kaszniak AW, et al. Clinical trial of indomethacin in Alzheimer's disease. Neurology. 1993;43:1609-1611.
30. Piette F, Belmin J, Vincent H, Schmidt N, Pariel S, Verny M, et al. Masitinib as an adjunct therapy for mild-to-moderate Alzheimer's disease: a randomised, placebo-controlled phase 2 trial. Alzheimers Res Ther. 2011;3:16.
31. Aisen PS, Davis KL, Berg JD, Schafer K, Campbell K, Thomas RG, et al. A randomized controlled trial of prednisone in Alzheimer's disease. Alzheimer's Disease Cooperative Study. Neurology. 2000;54:588-593.
32. Turner RS, Thomas RG, Craft S, van Dyck CH, Mintzer J, Reynolds BA, et al. A randomized, double-blind, placebo-controlled trial of resveratrol for Alzheimer disease. Neurology. 2015. doi:10.1212/WNL.0000000000002035.
33. Reines SA, Block GA, Morris JC, Liu G, Nessly ML, Lines CR, et al. Rofecoxib: no effect on Alzheimer's disease in a 1-year, randomized, blinded, controlled study. Neurology. 2004;62:66-71.
34. Aisen PS, Schafer KA, Grundman M, Pfeiffer E, Sano M, Davis KL, et al. Effects of rofecoxib or naproxen vs placebo on Alzheimer disease progression: a randomized controlled trial. JAMA. 2003;289:2819-2826.
35. Cucinotta D, De Leo D, Frattola L, Trabucchi M, Albizatti M, Beltramelli A, et al. Dihydroergokryptine as long-term treatment of Alzheimer type dementia: a multicenter two-year follow-up. Arch Gerontol Geriatr. 1998;Suppl 6:103-10.
36. Remington R, Bechtel C, Larsen D, Samar A, Doshanjh L, Fishman P, et al. A Phase II Randomized Clinical Trial of a Nutritional Formulation for Cognition and Mood in Alzheimer's Disease. J Alzheimers Dis. 2015;45:395-405.
37. Filip V, Kolibas E. Selegiline in the treatment of Alzheimer's disease: a long-term randomized placebo-controlled trial. Czech and Slovak Senile Dementia of Alzheimer Type Study Group. J Psychiatry Neurosci. 1999;24:234-43.
38. Freedman M, Rewilak D, Xerri T, Cohen S, Gordon AS, Shandling M, et al. L-deprenyl in Alzheimer's disease: cognitive and behavioral effects. Neurology. 1998;50:660-668.
39. Koivisto K, Helkala E-L, Hanninen T, Vanhanen M, Aaltonen H, Reinikainen K, et al. Three-year follow-up of long-term selegiline treatment of Alzheimer's disease. J Neurol. 1995;242:S34-S35.
40. Burke WJ, Roccaforte WH, Wengel SP, Bayer BL, Ranno AE, Willcockson NK. L-deprenyl in the treatment of mild dementia of the Alzheimer type: results of a 15-month trial. J Am Geriatr Soc. 1993;41:1219-25.
41. Sano M, Ernesto C, Thomas RG, Klauber MR, Schafer K, Grundman M, et al. A controlled trial of selegiline, alpha-tocopherol, or both as treatment for Alzheimer's disease. The Alzheimer's Disease Cooperative Study. New Engl J Med. 1997;336:1216-1222.
42. Tariot PN, Schneider LS, Cummings J, Thomas RG, Raman R, Jakimovich LJ, et al. Alzheimer's Disease Cooperative Study Group. Chronic divalproex sodium to attenuate agitation and clinical progression of Alzheimer disease. Arch Gen Psychiatry 2011;68:853-61.
43. Wischik CM, Staff RT, Wischik DJ, Bentham P, Murray AD, Storey JM, et al. Tau aggregation inhibitor therapy: an exploratory phase 2 study in mild or moderate Alzheimer's disease. J Alzheimers Dis. 2015;44:705-20.
44. Krishnan KR, Charles HC, Doraiswamy PM, Mintzer J, Weisler R, Yu X, et al. Randomized, placebo-controlled trial of the effects of donepezil on neuronal markers and hippocampal volumes in Alzheimer's disease. Am J Psychiatry. 2003;160:2003-11.
45. Burns A, Rossor M, Hecker J, Gauthier S, Petit H, Moller HJ, et al. The effects of donepezil in Alzheimer's disease - results from a multinational trial. Dement Geriatr Cogn Disord. 1999;10:237-44.
46. Rogers SL, Farlow MR, Doody RS, Mohs R, Friedhoff LT. A 24-week, double-blind, placebo-controlled trial of donepezil in patients with Alzheimer's disease. Donepezil Study Group. Neurology. 1998;50:136-45.
47. Raskind MA, Peskind ER, Wessel T, Yuan W. Galantamine in AD: A 6-month randomized, placebo-controlled trial with a 6-month extension. The Galantamine USA-1 Study Group. Neurology. 2000;54:2261-68.
48. A Phase 3 Study Evaluating Safety and Effectiveness of Immune Globulin Intravenous (IGIV 10%) for the Treatment of Mild-to-Moderate Alzheimer's Disease. ClinicalTrials.gov. 2015. http://www.clinicaltrials.gov/ct2/show/NCT00818662. Accessed 9 Oct 2015.
49. Relkin N. Results of the GAP 160701 study: A phase 3 clinical trial of intravenous immunoglobulin for mild-to-moderate Alzheimer's disease. Alzheimers Dement. 2013;9:530.
50. Safety and Efficacy Study Evaluating Dimebon in Patients With Mild to Moderate Alzheimer's Disease on Donepezil (CONCERT). ClinicalTrials.gov. 2012. http://www.clinicaltrials.gov/ct2/show/NCT00829374. Accessed 9 Oct 2015.
51. Sweetlove M. Phase III CONCERT Trial of Latrepirdine. Pharm Med. 2012;26:113-5.
52. A Safety and Efficacy Study of Oral Dimebon in Patients With Mild-To-Moderate Alzheimer's Disease (CONNECTION). ClinicalTrials.gov. 2012. http://www.clinicaltrials.gov/ct2/show/NCT00675623. Accessed 9 Oct 2015.
53. Pfizer And Medivation Announce Results From Two Phase 3 Studies In Dimebon (latrepirdine*) Alzheimer's Disease Clinical Development Program. Pfizer. 2010. http://press.pfizer.com/press-release/pfizer-and-medivation-announce-results-two-phase-3-studies-dimebon-latrepirdine-alzhei. Accessed 12 Oct 2015.
54. Doody RS, Gavrilova SI, Sano M, Thomas RG, Aisen PS, Bachurin SO, et al. Effect of dimebon on cognition, activities of daily living, behaviour, and global function in patients with mild-to-moderate Alzheimer's disease: a randomised, double-blind, placebo-controlled study. Lancet. 2008;372:207-15.
55. Alvarez XA, Cacabelos R, Laredo M, Couceiro V, Sampedro C, Varela M, et al. A 24-week, double-blind, placebo-controlled study of three dosages of Cerebrolysin in patients with mild to moderate Alzheimer's disease. Eur J Neurol. 2006;13:43-54.
56. Wang T, Huang Q, Reiman EM, Chen K, Li X, Li G, et al. Effects of memantine on clinical ratings, fluorodeoxyglucose positron emission tomography measurements, and cerebrospinal fluid assays in patients with moderate to severe Alzheimer dementia: a 24-week, randomized, clinical trial. J Clin Psychopharmacol. 2013;33:636-42.
57. Wilkinson D, Fox NC, Barkhof F, Phul R, Lemming O, Scheltens P. Memantine and brain atrophy in Alzheimer's disease: a 1-year randomized controlled trial. J Alzheimers Dis. 2012;29:459-69.
58. Ashford JW, Adamson M, Beale T, La D, Hernandez B, Noda A, et al. MR spectroscopy for assessment of memantine treatment in mild to moderate Alzheimer dementia. J Alzheimers Dis. 2011;26 Suppl 3:331-6.
59. Modrego PJ, Fayed N, Errea JM, Rios C, Pina MA, Sarasa M. Memantine versus donepezil in mild to moderate Alzheimer's disease: A randomized trial with magnetic resonance spectroscopy. Eur J Neurol. 2010;17:405-12.
60. Schmidt R, Ropele S, Pendl B, Ofner P, Enzinger C, Schmidt H, et al. Longitudinal multimodal imaging in mild to moderate Alzheimer disease: a pilot study with memantine. J Neurol Neurosurg Psychiatry. 2008;79:1312-7.
61. Gold M, Alderton C, Zvartau-Hind M, Egginton S, Saunders AM, Irizarry M, et al. Rosiglitazone monotherapy in mild-to-moderate Alzheimer's disease: results from a randomized, double-blind, placebo-controlled phase III study. Dement Geriatr Cogn Disord. 2010;30:131-46.
62. Risner ME, Saunders AM, Altman JF, Ormandy GC, Craft S, Foley IM, et al. Efficacy of rosiglitazone in a genetically defined population with mild-to-moderate Alzheimer's disease. Pharmacogenomics J. 2006;6:246-54.
63. Galasko D, Bell J, Mancuso JY, Kupiec JW, Sabbagh MN, van DC, et al. Clinical trial of an inhibitor of RAGE-Abeta interactions in Alzheimer disease. Neurology. 2014;82:1536-42.
64. Study evaluating the safety and efficacy of bapineuzumab in Alzheimer disease patients. ClinicalTrials.gov. 2012. http://www.clinicaltrials.gov/ct2/show/nct00676143. Accessed 22 Sep 2015.
65. Study evaluating the efficacy and safety of bapineuzumab in Alzheimer disease patients. ClinicalTrials.gov. 2013. http://www.clinicaltrials.gov/ct2/show/NCT00667810. Accessed 22 Sep 2015.
66. Trial of Carvedilol in Alzheimer's Disease. ClinicalTrials.gov. 2015. http://www.clinicaltrials.gov/ct2/show/NCT01354444. Accessed 9 Oct 2015.
67. A Study of Gantenerumab in Patients With Mild Alzheimer Disease. ClinicalTrials.gov. 2015. http://www.clinicaltrials.gov/ct2/show/NCT02051608. Accessed 9 Oct 2015.
68. Genistein as a Possible Treatment for Alzheimer's Disease. (GENISTEÍNA_2). ClinicalTrials.gov. 2015. http://www.clinicaltrials.gov/ct2/show/NCT01982578. Accessed 9 Oct 2015.
69. A Phase III Trial of Nilvadipine to Treat Alzheimer's Disease (NILVAD). ClinicalTrials.gov. 2015. http://www.clinicaltrials.gov/ct2/show/NCT02017340. Accessed 9 Oct 2015.
70. A Single Site, Randomized, Double-blind, Placebo Controlled Trial of NIC5-15 in Subjects with Alzheimer's Disease. ClinicalTrials.gov. 2013. http://www.clinicaltrials.gov/ct2/show/NCT01928420. Accessed 9 Oct 2015.
71. The clinical study of Pitavastatin treatment for group of mild to moderate Alzheimer's disease (PIT-ROAD). ClinicalTrials.gov. 2012. http://www.clincialtrials.gov/CT2/show/NCT00548145. Accessed 22 Sep 2015.
72. Progress of Mild Alzheimer's Disease in Participants on Solanezumab Versus Placebo (EXPEDITION 3). ClinicalTrials.gov. 2015. http://www.clinicaltrials.gov/ct2/show/NCT01900665. Accessed 9 Oct 2015.
73. Cholesterol lowering agents to slow progression (CLASP) of Alzheimer's disease study. ClinicalTrials.gov. 2009. http://www.clinicaltrials.gov/ct2/show/NCT00053599. Accessed 22 Sep 2015.
74. Wilcock G, Black S, Balch A, Amato D, Beelen A, Schneider L, et al. Safety and efficacy of tarenflurbil in subjects with mild Alzheimer's disease: results from an 18-month international multi-centre phase 3 trial. Alzheimers Dement. 2009;54:86.
75. European Study of 3APS in Mild to Moderate Alzheimer's Disease Patients. ClinicalTrials.gov. 2007. http://www.clinicaltrials.gov/ct2/show/NCT00217763. Accessed 9 Oct 2015.
76. An Efficacy and Safety Trial of Verubecestat (MK-8931) in Mild to Moderate Alzheimer's Disease (P07738) (EPOCH). ClinicalTrials.gov. 2015. http://www.clinicaltrials.gov/ct2/show/NCT01739348. Accessed 9 Oct 2015.
77. A Phase 3 Study to Evaluate the Safety and Efficacy of Masitinib in Patients with Mild to Moderate Alzheime'rs Disease. ClinicalTrials.gov. 2013. http://www.clinicaltrials.gov/ct2/show/NCT01872598. Accessed 9 Oct 2015.
78. Sunphenon EGCg (Epigallocatechin-Gallate) in the early stage of Alzheimer's disease (SUN-AK). ClinicalTrials.gov. 2015. http://www.clinicaltrials.gov/ct2/show/NCT00951834. Accessed 22 Sep 2015.
79. Neptune Krill Oil (NKO^TM^) in Early Stage Alzheimer's Disease (MNEMOSYNE). ClinicalTrials.gov. 2011. http://www.clinicaltrials.gov/ct2/show/NCT00867828. Accessed 9 Oct 2015.
80. Rasagiline Rescue in Alzheimer's Disease Clinical Trial (R2). ClinicalTrials.gov. 2015. http://www.clinicaltrials.gov/ct2/show/NCT02359552. Accessed 9 Oct 2015.
81. Randomized trial of a nutritional supplement in Alzheimer's disease. ClinicalTrials.gov. 2012. http://www.clinicaltrials.gov/ct2/show/NCT00678431. Accessed 22 Sep 2015.
82. Annweiler C, Fantino B, Parot-Schinkel E, Thiery S, Gautier J, Beauchet O. Alzheimer's disease - input of vitamin D with mEmantine assay (AD-IDEA trial): Study protocol for a randomized controlled trial. Trials. 2011;12:230.
83. Safety and Efficacy Study Evaluating TRx0237 in Subjects with Mild Alzheimer's Disease. ClinicalTrials.gov. 2015. http://www.clinicaltrials.gov/ct2/show/NCT01689233. Accessed 9 Oct 2015.
84. Safety and Efficacy Study Evaluating TRx0237 in Subjects with Mild to Moderate Alzheimer's Disease. ClinicalTrials.gov. 2014. http://www.clinicaltrials.gov/ct2/show/NCT01689246. Accessed 9 Oct 2015.
85. A Study to Evaluate Albumin and Immunoglobulin in Alzheimer's Disease (AMBAR). ClinicalTrials.gov. 2015. http://www.clinicaltrials.gov/ct2/show/NCT01561053. Accessed 9 Oct 2015.
86. AC-1204 26-Week Long Term Efficacy Response Trial with Optional Open-label Ext (NOURISH AD). ClinicalTrials.gov. 2015. http://www.clinicaltrials.gov/ct2/show/NCT01741194. Accessed 9 Oct 2015.
87. Riluzole in mild Alzheimer’s disease. ClinicalTrials.gov. 2015. http://www.clinicaltrials.gov/ct2/show/NCT01703117. Accessed 22 Sep 2015.
88. Evaluation of the Efficacy and Safety of Azeliragon (TTP488) in Patients with Mild Alzheimer's Disease (STEADFAST). 2015. http://www.clinicaltrials.gov/ct2/show/NCT02080364. Accessed 9 Oct 2015.
89. Mohs RC, Knopman D, Petersen RC, Ferris SH, Ernesto C, Grundman M, et al. Development of cognitive instruments for use in clinical trials of antidementia drugs: additions to the Alzheimer's Disease Assessment Scale that broaden its scope. The Alzheimer's Disease Cooperative Study. Alzheimer Dis Assoc Disord. 1997;11:S13-S21.
90. Galasko D, Bennett D, Sano M, Ernesto C, Thomas R, Grundman M, et al. An inventory to assess activities of daily living for clinical trials in Alzheimer's disease. The Alzheimer's Disease Cooperative Study. Alzheimer Dis Assoc Disord. 1997;11:S33-S39.
91. Morris JC. The Clinical Dementia Rating (CDR): current version and scoring rules. Neurology. 1993;43:2412-4.
92. Cummings JL, Mega M, Gray K, Rosenberg-Thompson S, Carusi DA, Gornbein J. The Neuropsychiatric Inventory: comprehensive assessment of psychopathology in dementia. Neurology. 1994;44:2308-14.
