# Supplementary material for: A review of clinical trial designs used to detect a disease-modifying effect of drug therapy in Alzheimer’s disease and Parkinson’s disease
Source: BMC Neurol. 2016 Jun 16;16:92. doi: 10.1186/s12883-016-0606-3 (PMC4910262; doi:10.1186/s12883-016-0606-3)
Supplement: Additional file 8: — Methods used to differentiate symptomatic from disease-modifying drug effects in all RCTs in PD. (DOCX 43 kb) [file 12883_2016_606_MOESM8_ESM.docx]

**Additional file 8: Overview of methods used to differentiate symptomatic from disease-modifying effects of putative disease-modifying agents in all included randomised controlled trials in Parkinson’s disease**

| **Trial** | **Wash-in analysis** | **Wash-out analysis** | **Delayed-start trial design** | **Long-term**  **follow-up** | **Biomarkers (primary or secondary outcome measures)** | | | | | | **Time-to-event outcomes** |
| --- | --- | --- | --- | --- | --- | --- | --- | --- | --- | --- | --- |
|  |  |  |  |  | **Imaging** | **CSF** | **Blood** | | **Urine** | **EEG** |  |
| PRECEPT [1] |  | 4 weeks |  | 24 months† | [^123^I]β-CIT SPECT |  |  | |  |  | Time to dopaminergic treatment |
| Green tea [2, 3] |  |  | 6 month delay |  |  |  |  | |  |  |  |
| TCH346 [4] | 4 weeks | 4 weeks |  | 12-18 months† |  |  |  | |  |  | Time to dopaminergic treatment |
| Exenatide [5] |  | 8 weeks |  |  | [^123^I]FP-CIT SPECT |  |  | |  |  |  |
| H_2_-water [6] |  | 8 weeks |  |  |  |  |  | |  |  |  |
| ROADS [7] | 4 and 13 weeks | Staggered  (2 or 4 weeks) |  | 12 months† |  |  |  | |  |  | Time to levodopa treatment |
| Subtherapeutic pergolide [8] | 6 weeks | 4 weeks |  | Until all required levodopa† |  |  |  | |  |  | Time to levodopa treatment |
| CALM-PD-CIT [9] |  |  |  | 46 months† | [^123^I]β-CIT SPECT |  |  | |  |  |  |
| Rasagiline cognition [10] |  |  |  | 6 monthsФ |  |  |  | |  |  |  |
| Russian rasagiline [11] |  | 4 weeks |  |  |  |  |  | |  |  |  |
| ADAGIO [12] |  |  | 8 month delay | 18 months‡ |  |  |  | |  |  |  |
| TEMPO [13] |  |  | 6 month delay | 12 months† |  |  |  | |  |  | Time to dopaminergic treatment |
| REAL-PET [14] |  |  |  | 24 months† | FDOPA PET |  |  | |  |  | Time to dyskinesias |
| UK/France FDOPA PET [15] |  |  |  |  | FDOPA PET |  |  | |  |  |  |
| Norwegian-Danish [16] |  | 4 weeks |  | 60 months† |  |  |  | |  |  | Time to motor fluctuations |
| SELEDO [17] |  |  |  | 60 months† |  |  |  | |  |  | Time until baseline levodopa dose had to be increased by ≥ 50% |
| Swedish selegiline [18] | 6 and 12 weeks | 8 weeks |  | Until all required levodopa† |  |  |  | |  |  | Time to levodopa treatment |
| SINDEPAR [19] | 12 weeks | 8 weeks |  |  |  |  |  | |  |  |  |
| Finnish selegiline [20] | 4 and 8 weeks |  |  | Until all required levodopa† |  |  |  | |  |  | Time to levodopa treatment |
| Tetrud and Langston [21] | 4 weeks | 4 weeks |  | 36 months† |  |  |  | |  |  | Time to levodopa treatment |
| DATATOP [22] | 4 and 12 weeks | 4 and 8 weeks |  | 24 months† |  |  |  | |  |  | Time to levodopa treatment |
| QE3 [23] |  |  |  | 16 months† |  |  |  | |  |  | Time to dopaminergic treatment |
| QE2 [24] | 4 weeks |  |  | 16 months† |  |  |  | |  |  | Time to levodopa treatment |
| Creatine-CoQ10 [25] |  |  |  | 18 months¥ |  |  | Phospholipids | |  |  |  |
| NET-PD LS-1 Creatine [26] |  |  |  | 60-96 months¥ |  |  |  | |  |  |  |
| German Creatine [27] |  |  |  |  | [^123^I]FP-CIT SPECT |  |  | |  |  |  |
| Fenugreek [28] |  |  |  | 6 months¥ |  |  |  | |  |  |  |
| **Trial** | **Wash-in analysis** | **Wash-out analysis** | **Delayed-start trial design** | **Long-term**  **follow-up** | **Biomarkers (primary or secondary outcome measures)** | | | | | | **Time-to-event outcomes** |
|  |  |  |  |  | **Imaging** | **CSF** | | **Blood** | **Urine** | **EEG** |  |
| Ubiquinol-10 [29] | 8 weeks | 8 weeks |  | 48 weeks† (n=31)  96 weeks† (n=33) |  |  | |  |  |  | Time to levodopa treatment |
| FAIRPARK [30] |  |  | 6 month delay |  | MRI: R2* |  | |  |  |  |  |
| ELLDOPA [31] |  | 2 weeks |  | 10 months† | [^123^I]β-CIT SPECT |  | |  |  |  |  |
| PROUD [32] |  |  | 6-9 month delay |  | [^123^I]FP-CIT SPECT |  | |  |  |  |  |
| GM1 ganglioside [33] |  | 1 and 2 years | 6 month delay | 52 months† | [^11^C]MP PET [34] |  | |  |  |  |  |
| MitoQ trial [35] | 4 weeks |  |  | 12 months† |  |  | |  |  |  | Time to dopaminergic treatment |
| Riluzole international [36] |  | 8 weeks |  |  | FDOPA PET |  | |  |  |  | Time to dopaminergic treatment |
| Riluzole USA [37] |  | 6 weeks | 6 month delay | 12 months† |  |  | |  |  |  | Time to dopaminergic treatment |
| GPI-1485 (6 month trial) [38] |  |  |  |  | [^123^I]β-CIT SPECT |  | |  |  |  |  |
| **Planned, Ongoing and**  **Unpublished RCTs** |  |  |  |  |  |  | |  |  |  |  |
| MIREILLE [39] |  |  |  | 12 months | [^123^I]FP-CIT SPECT |  | |  |  |  |  |
| Exenatide-PD [40] |  | 12 weeks |  |  | [^123^I]FP-CIT SPECT | BDNF | |  |  |  |  |
| STEADY-PD [41] |  |  |  | 36 months |  |  | |  |  |  | Time to dopaminergic treatment  Time to motor complications |
| G-CSF [42] |  |  |  | 24 months |  |  | |  |  |  |  |
| ZONIST [43] |  |  |  |  |  |  | |  |  |  | Time to dopaminergic treatment |
| NICOPARK2 [44] |  | 5 weeks |  |  | [^123^I]FP-CIT SPECT |  | |  |  |  |  |
| NIC-PD [45] |  | 8 weeks |  | 14 months |  |  | |  |  |  | Time to dopaminergic treatment |
| GPI-1485 (2 year trial) [46] |  |  |  |  | [^123^I]β-CIT SPECT |  | |  |  |  |  |

**Key**

**Long-term follow-up studies (published studies only)**

**Biomarker modalities** † Image published (e.g. Kaplan Meier plot) from which the presence/absence of sustained

CSF Cerebrospinal fluid divergence in outcome measures could be inferred. No formal slope analyses conducted.

EEG Electroencephalography ‡ Formal slope analyses conducted to look for sustained divergence.

MRI Magnetic Resonance Imaging ¥ No formal slope analysis conducted nor image published from which sustained divergence

PET Positron Emission Tomography in outcome measures between groups can be inferred. Furthermore, no alternative

SPECT Single Photon Emission Computed Tomography strategy used to try to demonstrate disease-modification.

Ф Insufficient information to classify (e.g. only published as conference abstract)

**CSF measurements MRI measurements**

BDNF Brain-Derived Neurotrophic Factor R2* Proton transverse relaxation rate

**PET ligands**

FDOPA [^18^F]6-fluoro-L-3,4-dihydroxyphenylalanine

[^11^C]MP [^11^C]Methylphenidate

**SPECT ligands**

[^123^I]FP-CIT [^123^I]-2β-carbomethoxy-3β-(4-iodophenyl)-N-(3-fluoropropyl)-N-tropane

[^123^I]β-CIT [^123^I]-2β-carbomethoxy-3β-(4-iodophenyl tropane)

**References**

1. Parkinson Study Group. Mixed lineage kinase inhibitor CEP-1347 fails to delay disability in early Parkinson disease. Neurology. 2007;69:1480-90.
2. Efficacy and safety of green tea polyphenol in de novo Parkinson's disease patients. ClinicalTrials.gov. 2011. http://www.clinicaltrials.gov/ct2/show/NCT00461942. Accessed 22 Sep 2015.
3. Ability to Slow Disease Progression and Safety and Tolerability of Green Tea Polyphenols in Early Parkinson's Disease. The Michael J.Fox Foundation for Parkinson's Research. 2013. https://www.michaeljfox.org/foundation/grant-detail.php?grant_id=187. Accessed 22 Sep 2015.
4. Olanow CW, Schapira AH, Lewitt PA, Kieburtz K, Sauer D, Olivieri G, et al. TCH346 as a neuroprotective drug in Parkinson's disease: a double-blind, randomised, controlled trial. Lancet Neurol. 2006;5:1013-20.
5. Aviles-Olmos I, Dickson J, Kefalopoulou Z, Djamshidian A, Ell P, Soderlund T, et al. Exenatide and the treatment of patients with Parkinson's disease. J Clin Invest. 2013;123:2730-6.
6. Yoritaka A, Takanashi M, Hirayama M, Nakahara T, Ohta S, Hattori N. Pilot study of H_2_ therapy in Parkinson's disease: A randomized double-blind placebo-controlled trial. Mov Disord. 2013;28:836-9.
7. The Parkinson Study Group. Effect of lazabemide on the progression of disability in early Parkinson's disease. Ann Neurol. 1996;40:99-107.
8. Grosset K, Grosset D, Lees A, Parkinson's Disease Research Group of the United Kingdom. Trial of subtherapeutic pergolide in de novo Parkinson's disease. Mov Disord. 2005;20:363-6.
9. Parkinson Study Group. Dopamine transporter brain imaging to assess the effects of pramipexole vs levodopa on Parkinson disease progression. JAMA. 2002;287:1653-61.
10. The Effect of Rasagiline on Cognition in Parkinson's Disease. ClinicalTrials.gov. 2015. http://www.clinIcaltrials.gov/ct2/show/NCT01382342. Accessed 9 Oct 2015.
11. Illarioshkin S, Karabanov A, Mirkasimov A, Verejutina I. Rasagiline in drug-nave Russian patients with early Parkinson's disease. Mov Disord. 2012;27:380.
12. Olanow CW, Rascol O, Hauser R, Feigin PD, Jankovic J, Lang A, et al. A double-blind, delayed-start trial of rasagiline in Parkinson's disease. N Eng J Med. 2009;361:1268-78.
13. Parkinson Study Group. A controlled, randomized, delayed-start study of rasagiline in early Parkinson disease. Arch Neurol. 2004;61:561-6.
14. Whone AL, Watts RL, Stoessl AJ, Davis M, Reske S, Nahmias C, et al. Slower progression of Parkinson's disease with ropinirole versus levodopa: The REAL-PET study. Ann Neurol. 2003;54:93-101.
15. Rakshi JS, Pavese N, Uema T, Ito K, Morrish PK, Bailey DL, et al. A comparison of the progression of early Parkinson's disease in patients started on ropinirole or L-dopa: an 18F-dopa PET study. J Neural Transm. 2002;109:1433-43.
16. Larsen JP, Boas J, Erdal JE. Does selegiline modify the progression of early Parkinson's disease? Results from a five-year study. The Norwegian-Danish Study Group. Eur J Neurol. 1999;6:539-547.
17. Przuntek H, Conrad B, Dichgans J, Kraus PH, Krauseneck P, Pergande G, et al. SELEDO: a 5-year long-term trial on the effect of selegiline in early Parkinsonian patients treated with levodopa. Eur J Neurol. 1999;6:141-150.
18. Palhagen S, Heinonen EH, Hagglund J, Kaugesaar T, Kontants H, Maki-Ikola O, et al. Selegiline delays the onset of disability in de novo parkinsonian patients. Swedish Parkinson Study Group. Neurology 1998;51:520-5.
19. Olanow CW, Hauser RA, Gauger L, Malapira T, Koller W, Hubble J, et al. The effect of deprenyl and levodopa on the progression of Parkinson's disease. Ann Neurol. 1995;38:771-7.
20. Myllyla VV, Sotaniemi KA, Vuorinen JA, Heinonen EH. Selegiline as initial treatment in de novo parkinsonian patients. Neurology. 1992;42:339-43.
21. Tetrud JW, Langston JW. The effect of deprenyl (selegiline) on the natural history of Parkinson's disease. Science. 1989;245:519-22.
22. The Parkinson Study Group. Effects of tocopherol and deprenyl on the progression of disability in early Parkinson's disease. N Eng J Med. 1993;328:176-83.
23. Beal MF, Oakes D, Shoulson I, Henchcliffe C, Galpern WR, Haas R, et al. A randomized clinical trial of high-dosage coenzyme Q10 in early Parkinson disease: no evidence of benefit. JAMA Neurol. 2014;71:543-52.
24. Shults CW, Oakes D, Kieburtz K, Beal MF, Haas R, Plumb S, et al. Effects of coenzyme Q10 in early Parkinson disease: evidence of slowing of the functional decline. Arch Neurol. 2002;59:1541-50.
25. Li Z, Wang P, Yu Z, Cong Y, Sun H, Zhang J, et al. The effect of creatine and coenzyme q10 combination therapy on mild cognitive impairment in Parkinson's disease. Eur Neurol. 2015;73:205-211.
26. Kieburtz K, Tilley BC, Elm JJ, Babcock D, Hauser R, Ross GW, et al. Effect of creatine monohydrate on clinical progression in patients with Parkinson disease: a randomized clinical trial. JAMA. 2015;313:584-93.
27. Bender A, Koch W, Elstner M, Schombacher Y, Bender J, Moeschl M, et al. Creatine supplementation in Parkinson disease: a placebo-controlled randomized pilot trial. Neurology. 2006;67:1262-4.
28. Nathan J, Panjwani S, Mohan V, Joshi V, Thakurdesai PA. Efficacy and safety of standardized extract of Trigonella foenum-graecum L seeds as an adjuvant to L-Dopa in the management of patients with Parkinson's disease. Phytother Res. 2014;28:172-8.
29. Yoritaka A, Kawajiri S, Yamamoto Y, Nakahara T, Ando M, Hashimoto K, et al. Randomized, double-blind, placebo-controlled pilot trial of reduced coenzyme Q10 for Parkinson's disease. Parkinsonism Relat Disord. 2015;21:911-6.
30. Devos D, Moreau C, Devedjian JC, Kluza J, Petrault M, Laloux C, et al. Targeting chelatable iron as a therapeutic modality in Parkinson's disease. Antioxid Redox Signal. 2014;21:195-210.
31. Fahn S, Oakes D, Shoulson I, Kieburtz K, Rudolph A, Lang A, et al. Levodopa and the progression of Parkinson's disease. N Eng J Med. 2004;351:2498-508.
32. Schapira AH, McDermott MP, Barone P, Comella CL, Albrecht S, Hsu HH, et al. Pramipexole in patients with early Parkinson's disease (PROUD): a randomised delayed-start trial. Lancet Neurol. 2013;12:747-55.
33. Schneider JS, Gollomp SM, Sendek S, Colcher A, Cambi F, Du W. A randomized, controlled, delayed start trial of GM1 ganglioside in treated Parkinson's disease patients. J Neurol Sci. 2013;324:140-8.
34. Schneider JS, Cambi F, Gollomp SM, Kuwabara H, Brasic JR, Leiby B, et al. GM1 ganglioside in Parkinson's disease: Pilot study of effects on dopamine transporter binding. J Neurol Sci. 2015;356:118-23.
35. Snow BJ, Rolfe FL, Lockhart MM, Frampton CM, O'Sullivan JD, Fung V, et al. A double-blind, placebo-controlled study to assess the mitochondria-targeted antioxidant MitoQ as a disease-modifying therapy in Parkinson's disease. Mov Disord. 2010;25:1670-4.
36. Rascol O, Olanow W, Brooks D, Koch P, Truffinet R, Bejuit R. A 2-year, multicenter, placebo-controlled, double-blind, parallel-group study of the effect of riluzole on Parkinson's disease progression. Mov Disord. 2002;17:S39.
37. Jankovic J, Hunter C. A double-blind, placebo-controlled and longitudinal study of riluzole in early Parkinson's disease. Parkinsonism Relat Disord. 2002;8:271-6.
38. Guilford Pharmaceuticals Inc: Final phase II GPI 1485 (NIL-A) imaging data presented at the annual meeting of the American Academy of Neurology. PR Newswire. 2002. http://www.prnewswire.co.uk/news-releases/final-phase-ii-gpi-1485-nil-a-imaging-data-presented-at-the-annual-meeting-of-the-american-academy-of-neurology-155593265.html. Accessed 22 Sep 2015.
39. Bee venom for the treatment of Parkinson disease (MIREILLE). ClinicalTrials.gov. 2014. http://www.clinicaltrials.gov/ct2/show/NCT01341431. Accessed 22 Sep 2015.
40. Trial of Exenatide for Parkinson's Disease (EXENATIDE-PD). ClinicalTrials.gov. 2015. http://www.clinicaltrials.gov/ct2/show/NCT01971242. Accessed 22 Sep 2015.
41. Efficacy of Isradipine in Early Parkinson's Disease ClinicalTrials.gov. 2015. http://www.clinicaltrials.gov/ct2/show/NCT02168842. Accessed 22 Sep 2015.
42. Study of the neuro-protective effect of Granulocyte-colony Stimulating Factor on early stage Parkinson's disease. ClinicalTrials.gov. 2013. http://www.clinicaltrials.gov/ct2/show/NCT01227681. Accessed 22 Sep 2015.
43. Study of zonisamide in early Parkinson disease (ZONIST). ClinicalTrials.gov. 2013. http://www.clinicaltrials.gov/ct2/show/NCT01766128. Accessed 22 Sep 2015.
44. Efficacy of transdermal nicotine, on motor symptoms in advanced Parkinson's disease (NICOPARK2). ClinicalTrials.gov. 2013. http://www.clinicaltrials.gov/ct2/show/NCT00873392. Accessed 22 Sep 2015.
45. Disease-modifying Potential of Transdermal NICotine in Early Parkinson's Disease (NIC-PD). ClinicalTrials.gov. 2014. http://www.clinicaltrials.gov/ct2/show/NCT01560754. Accessed 22 Sep 2015.
46. 2 year study to evaluate the effects of GPI 1485 on [123I]b-CIT/SPECT scanning and clinical efficacy in patients with PD. ClinicalTrials.gov. 2008. http://www.clinicaltrials.gov/ct2/show/NCT00209508. Accessed 22 Sep 2015.
