# Supplementary material for: A review of clinical trial designs used to detect a disease-modifying effect of drug therapy in Alzheimer’s disease and Parkinson’s disease
Source: BMC Neurol. 2016 Jun 16;16:92. doi: 10.1186/s12883-016-0606-3 (PMC4910262; doi:10.1186/s12883-016-0606-3)
Supplement: Additional file 9: — Key design features and outcome measures in published AD RCTs. (DOCX 63 kb) [file 12883_2016_606_MOESM9_ESM.docx]

**Additional file 9: Key design features and outcome measures used in published randomised controlled trials of putative disease-modifying agents in Alzheimer’s disease**

| **Trial** | **Primary outcome measures** | **Biomarkers or time-to-event outcomes used as secondary outcome measures** | **Methods used to deal with deaths and drop-outs (primary outcome)** | | | | | | **Methods used to differentiate symptomatic from disease-modifying effects of the agent** |
| --- | --- | --- | --- | --- | --- | --- | --- | --- | --- |
|  |  |  | **ITT** | **Complete-case analysis** | **Survival analysis** | **LOCF** | **Mixed model** | **Data**  **imputation** |  |
| Aβ immunisation [1] | ADAS-cog  MRI: whole brain vol. | CSF: Aβ  CSF: total-tau | X | NR | NR | NR | NR | NR | Long-term follow-up. Imaging and CSF biomarkers.  [n=22 for CSF] |
| LEADe [2] | ADCS-CGIC  ADAS-cog | MRI: hippocampal vol.  MRI: whole brain vol. | ✓ | X | X | X | ✓ | X | Long-term follow-up.  Analysis of primary and secondary outcomes after wash-out (8 weeks) period.  Imaging biomarker. |
| Bapineuzumab (phase 3) APOE ε4 carriers [3] | ADAS-cog  DAD | CSF: phospho-tau  MRI: whole brain vol.  PiB PET: amyloid load | ✓ | X | X | X | ✓ | X | Long-term follow-up.  Imaging and CSF biomarkers.  [n=212 for CSF, n=590 for MRI and n=115 for PiB PET] |
| Bapineuzumab (phase 3) APOE ε4 non-carriers [3] | ADAS-cog  DAD | CSF: phospho-tau  MRI: whole brain vol.  PiB PET: amyloid load | ✓ | X | X | X | ✓ | X | Long-term follow-up.  Imaging and CSF biomarkers.  [n=178 for CSF, n=559 for MRI and n=39 for PiB PET] |
| Bapineuzumab (phase 2 )[4] | ADAS-cog  CDR-SB  DAD  MMSE | CSF: Aβ42  CSF: phospho-tau and total-tau  MRI: ventricular vol.  MRI: whole brain vol. | ✓ | X | X | X | ✓ | X | Long-term follow-up.  Imaging and CSF biomarkers.  [n=35 for CSF] |
| ABBY [5, 6] | ADAS-cog  CDR-SB | CSF: details unclear  MRI: details unclear | NR | NR | NR | NR | NR | NR | Long-term follow-up.  Imaging and CSF biomarkers. |
| BLAZE [6-8] | AV-45 PET | CSF: details unclear  FDG PET  Volumetric MRI | NR | NR | NR | NR | NR | NR | Long-term follow-up.  Imaging and CSF biomarkers. |
| Scyllo-inositol [9] | ADCS-ADL  MRI: ventricular vol.  NTB | CSF: Aβ40 and Aβ42  CSF: phospho-tau and total tau  MRI: cortical ribbon thickness  MRI: hippocampal vol.  MRI: whole brain vol.  MRS: scyllo-inositol and MI | ✓ | X | X | X | ✓ | X | Long-term follow-up.  Imaging and CSF biomarkers. |

| **Trial** | **Primary outcome measures** | **Biomarkers or time-to-event outcomes used as secondary outcome measures** | **Methods used to deal with deaths and drop-outs (primary outcome)** | | | | | | **Methods used to differentiate symptomatic from disease-modifying effects of the agent** | |
| --- | --- | --- | --- | --- | --- | --- | --- | --- | --- | --- |
|  |  |  | **ITT** | **Complete-case analysis** | **Survival analysis** | **LOCF** | **Mixed model** | **Data**  **imputation** |  |  |
| IDENTITY [10, 11] | ADAS-cog  ADCS-ADL | AV-45 PET: amyloid load  CSF: Aβ42  CSF: phospho-tau and tau  FDG PET  Plasma: Aβ  Volumetric MRI | ✓ | X | X | X | ✓ | X | Randomised delayed-start design: the group initially treated with placebo commenced active treatment after 18 months.  Analysis of primary outcomes after wash-out (16 weeks) period.  Imaging, CSF and blood biomarkers.  [n=47 for CSF, n=208 for MRI, n=844 for plasma, n=59 for PET] | |
| IDENTITY2 [12] | ADAS-cog  ADCS-ADL | AV-45 PET: amyloid load  CSF: Aβ42  CSF: phospho-tau and tau  FDG PET  Plasma: Aβ  Volumetric MRI | NR | NR | NR | NR | NR | NR | Randomised delayed-start design: the group initially treated with placebo commenced active treatment after 18 months.  Analysis of primary outcomes after wash-out (16 weeks) period.  Imaging, CSF and blood biomarkers. | |
| Simvastatin [13] | CSF: Aβ40  CSF: Aβ42 | - | X | ✓ | X | X | X | X | CSF biomarker. | |
| EXPEDITION 1 [14] | ADAS-cog  ADCS-ADL | AV-45 PET  CSF: Aβ40 and Aβ42  CSF: phospho-tau and tau  Plasma: Aβ40 and Aβ42  Volumetric MRI | ✓ | X | X | X | ✓ | X | Long-term follow-up.  Imaging, CSF and blood biomarkers. | |
| EXPEDITION 2 [14] | ADAS-cog  ADCS-ADL | AV-45 PET  CSF: Aβ40 and Aβ42  CSF: phospho-tau and tau  Plasma: Aβ40 and Aβ42  Volumetric MRI | ✓ | X | X | X | ✓ | X | Long-term follow-up.  Imaging, CSF and blood biomarkers. | |
| Tarenflurbil (phase 3) [15] | ADCS-ADL  ADAS-cog | - | ✓ | X | X | X | ✓ | Imputed missing data with values the same number of SDs from the treatment group mean as each participants last observed values. | Long-term follow-up.  In addition to analysing the change in primary and secondary outcome measures over the study period slope analyses were conducted to explore the possibility of disease modification. | |
| **Trial** | **Primary outcome measures** | **Biomarkers or time-to-event outcomes used as secondary outcome measures** | **Methods used to deal with deaths and drop-outs (primary outcome)** | | | | | | | **Methods used to differentiate symptomatic from disease-modifying effects of the agent** |
|  |  |  | **ITT** | **Complete-case analysis** | **Survival analysis** | **LOCF** | **Mixed model** | **Data**  **imputation** | |  |
| Tarenflurbil (phase 2) [16] | ADCS-ADL  ADAS-cog  CDR-SB | - | ✓ | X | X | X | ✓ | X | | Long-term follow-up.  Randomised delayed-start design: the group initially treated with placebo commenced active treatment after 12 months.  Only Canadian patients (n=86) entered the 12-24 months follow-up phase. |
| Alphase [17] | ADAS-cog  CDR-SB  MRI: hippocampal vol. | CSF, plasma and urine: Aβ  CSF: tau  MRI: entorhinal cortex vol.  MRI: whole brain vol. | ✓ | X | X | ✓ | ✓ | X | | CSF and imaging biomarkers.  [n=312 for MRI] |
| DARAD [18] | CDR-SB  SADAS-cog | - | ✓ | X | X | X | ✓ | X | | Long-term follow-up. |
| T-817MA [19] | ADAS-cog | MRI: hippocampal vol.  MRI: whole brain vol. | NR | NR | NR | NR | NR | NR | | Long-term follow-up.  Imaging biomarker.  [n=17 for MRI] |
| Celecoxib [20] | ADAS-cog  CIBIC+  Time to the following separate endpoints: (1) 4 point decline on ADAS-cog; (2) 5 point decline on CIBIC+. | - | ✓ | ✓ | ✓ | X | X | X | | Long-term follow-up.  Time-to-event outcomes. |
| DAD2000 [21] | ADAS-cog | - | NR | NR | NR | NR | NR | NR | | Long-term follow-up. |
| Diclofenac + misoprostol [22] | ADAS-cog  CGIC  GDS | - | ✓ | X | X | X | X | Week 12 observations used as the final observation for those who withdrew early. The time of the last observation was included in the model to adjust for confounding by time. | | Long-term follow-up. |

| **Trial** | **Primary outcome measures** | **Biomarkers or time-to-event outcomes used as secondary outcome measures** | **Methods used to deal with deaths and drop-outs (primary outcome)** | | | | | | **Methods used to differentiate symptomatic from disease-modifying effects of the agent** |
| --- | --- | --- | --- | --- | --- | --- | --- | --- | --- |
|  |  |  | **ITT** | **Complete-case analysis** | **Survival analysis** | **LOCF** | **Mixed model** | **Data**  **imputation** |  |
| Docosahexaenoic acid [23] | ADAS-cog  CDR-SB | MRI: hippocampal vol.  MRI: ventricular vol.  MRI: whole brain vol. | ✓ | X | X | X | ✓ | X | Long-term follow-up.  Imaging biomarker.  [n=102 for MRI] |
| OmegAD [24] | ADAS-cog  MMSE | - | ✓ | X | X | ✓ | X | X | Long-term follow-up.  Randomised delayed-start design: the group initially treated with placebo commenced active treatment after 6 months. |
| Escitalopram [25] | MRI: hippocampal vol.  MRI: whole brain vol. | - | NR | NR | NR | NR | NR | NR | Imaging biomarker. |
| Hydroxychloroquine [26] | IDDD | - | ✓ | X | X | X | X | Missing 18-month scores estimated from the 9-month change or, If also missing, from the baseline score and regression function of the baseline and 18-month scores of those with complete data. | Long-term follow-up. |
| Ibuprofen [27] | ADAS-cog | - | ✓ | X | X | ✓ | X | X | Long-term follow-up. |
| Dutch indomethacin [28] | ADAS-cog | - | X | X | X | ✓ | X | X | Long-term follow-up. |
| American indomethacin [29] | ADAS  BNT  MMSE  Token Test | - | X | ✓ | X | X | X | X | Long-term follow-up. |
| Masitinib (phase 2) [30] | ADAS-cog | - | ✓ | X | X | ✓ | X | X | Long-term follow-up. |

| **Trial** | **Primary outcome measures** | **Biomarkers or time-to-event outcomes used as secondary outcome measures** | **Methods used to deal with deaths and drop-outs (primary outcome)** | | | | | | **Methods used to differentiate symptomatic from disease-modifying effects of the agent** |
| --- | --- | --- | --- | --- | --- | --- | --- | --- | --- |
|  |  |  | **ITT** | **Complete-case analysis** | **Survival analysis** | **LOCF** | **Mixed model** | **Data**  **imputation** |  |
| Prednisone [31] | ADAS-cog | - | ✓ | X | X | X | X | Missing data imputed using an estimate of the change over the unobserved period based on all individuals with data in the subject’s treatment arm. | Long-term follow-up. |
| Resveratrol [32] | CSF: Aβ40, Aβ42  CSF: phospho-tau, tau  Plasma: Aβ40, Aβ42  MRI:  entorhinal cortex vol.  hippocampal vol.  ventricular vol.  whole brain vol. | - | ✓ | X | X | X | ✓ | X | CSF, blood and imaging biomarkers.  [n=77 for CSF] |
| Rofecoxib [33] | ADAS-cog  CIBIC+ | - | ✓ | X | X | X | ✓ | X | Long-term follow-up.  Analysis of primary outcomes after wash-out (12 weeks) period.  After the primary study 90% of patients in the rofecoxib group were switched to placebo for 12 weeks, while the others remained on their previous treatment until month 15. |
| Rofecoxib or naproxen [34] | ADAS-cog | Time to the following separate endpoints: (1) 4 point decline in ADAS-cog; (2) 1 step worsening on global CDR; (3) 15 point decline on ADCS-ADL; (4) institutionalisation; (5)death | ✓ | Conducted in addition to separate analyses using LOCF and data imputation. | X | ✓ | X | Missing data imputed by estimating the change over the unobserved period from all individuals with data in the subject’s treatment group. | Analysis of primary outcomes after wash-out (8 weeks) period.  Time-to-event outcomes. |
| DAV.I.D.E. [35] | Gottfries-Bråne-Steen Scale | - | X | ✓ | X | X | X | X | Long-term follow-up.  Randomised delayed-start design: the group initially treated with placebo commenced active treatment after 12 months. |
| **Trial** | **Primary outcome measures** | **Biomarkers or time-to-event outcomes used as secondary outcome measures** | **Methods used to deal with deaths and drop-outs (primary outcome)** | | | | | | **Methods used to differentiate symptomatic from disease-modifying effects of the agent** |
|  |  |  | **ITT** | **Complete-case analysis** | **Survival analysis** | **LOCF** | **Mixed model** | **Data**  **imputation** |  |
| Nutritional formulation [36] | CLOX  DRS | - | NR | NR | NR | NR | NR | NR | Randomised delayed-start design: the group initially treated with placebo commenced active treatment after 3-6 months. |
| Czech/Slovak selegiline [37] | Sternberg’s Memory Scanning Test | EEG: mean frequency of α and other dominant frequencies | X | ✓ | X | X | X | X | Long-term follow-up.  Analysis of primary outcomes after wash-in (6 and 12 weeks) periods.  EEG biomarker. |
| Canadian selegiline [38] | BPRS | - | X | ✓ | X | X | X | X | Analysis of primary outcomes after wash-out (12 weeks) period.  Before entering the randomised controlled phase of the study both groups received placebo for 4 weeks. |
| KUOSTAD [39] | MMSE | - | ✓ | ✓ | X | X | X | X | Long-term follow-up. |
| Nebraska selegiline [40] | Blessed Dementia Scale CDR-SB  Global CDR  MMSE | - | X | ✓ | X | X | X | X | Long-term follow-up. |
| Selegiline & tocopherol [41] | Time to any of the following: death, institutionalization, loss of ability to perform basic ADLs, or severe dementia (Global CDR = 3) | - | ✓ | X | ✓ | X | X | X | Long-term follow-up.  Time-to-event outcome. |
| VALID [42] | Time to clinically significant agitation or psychosis (score of ≥ 3 on ≥ 1 NPI items assessing delusions, hallucinations and agitation/aggression). | MRI: hippocampal vol.  MRI: ventricular vol.  MRI: whole brain vol. | ✓ | X | ✓ | X | X | X | Long-term follow-up.  Time-to-event outcome.  Analysis of secondary clinical outcome measures after wash-out (2 months) period.  Imaging biomarker.  [n=88 for MRI] |

| **Trial** | **Primary outcome measures** | **Biomarkers or time-to-event outcomes used as secondary outcome measures** | **Methods used to deal with deaths and drop-outs (primary outcome)** | | | | | | **Methods used to differentiate symptomatic from disease-modifying effects of the agent** |
| --- | --- | --- | --- | --- | --- | --- | --- | --- | --- |
|  |  |  | **ITT** | **Complete-case analysis** | **Survival analysis** | **LOCF** | **Mixed model** | **Data imputation** |  |
| TauRx (phase 2) [43] | ADAS-cog | HMPAO SPECT | ✓ | X | X | ✓ | X | X | Long-term follow-up.  Imaging biomarker.  [n=135 for SPECT] |
| Donepezil MRI/MRS [44] | MRS: N-acetylaspartate | MRI: hippocampal vol.  MRS: MI | ✓ | X | X | ✓ | X | X | Long-term follow-up.  Imaging biomarkers.  Analysis of primary outcomes after wash-out (6 weeks) period. |
| Donepezil international [45] | ADAS-cog  CIBIC+ | - | ✓ | X | X | ✓ | X | X | Long-term follow-up.  Analysis of primary and secondary outcomes after wash-out (6 weeks) period. |
| Donepezil USA clinical [46] | ADAS-cog  CIBIC+ | - | ✓ | X | X | ✓ | X | X | Long-term follow-up.  Analysis of primary outcomes and secondary outcomes after wash-out (6 weeks) period. |
| Galantamine [47] | ADAS-cog  CIBIC+ | - | ✓ | X | X | ✓ | X | X | Long-term follow-up. Randomised delayed-start design: the group initially treated with placebo commenced active treatment after 6 months. After 6 months all patients were treated with the lower of the two doses of galantamine given in the first 6 months. |
| GAP Study [48, 49] | ADAS-cog  ADCS-ADL | AV-45 PET  CSF: Aβ42  CSF: phospho-tau and tau  FDG PET | NR | NR | NR | NR | NR | NR | Long-term follow-up.  Imaging and CSF biomarkers. |
| CONCERT [50, 51] | ADAS-cog  ADCS-ADL | - | NR | NR | NR | NR | NR | NR | Long-term follow-up. |
| CONNECTION [52, 53] | ADAS-cog  CIBIC+ | - | NR | NR | NR | NR | NR | NR | Long-term follow-up. |
| Russian Dimebon [54] | ADAS-cog | - | ✓ | Conducted in addition to separate analyses using LOCF. | X | ✓ | X | X | Long-term follow-up. |

| **Trial** | **Primary outcome measures** | **Biomarkers or time-to-event outcomes used as secondary outcome measures** | **Methods used to deal with deaths and drop-outs (primary outcome)** | | | | | | **Methods used to differentiate symptomatic from disease-modifying effects of the agent** |
| --- | --- | --- | --- | --- | --- | --- | --- | --- | --- |
|  |  |  | **ITT** | **Complete-case analysis** | **Survival analysis** | **LOCF** | **Mixed model** | **Data imputation** |  |
| Cerebrolysin [55] | ADAS-cog  CIBIC+ | - | ✓ | X | X | ✓ | X | X | Long-term follow-up.  Analysis of primary outcomes after wash-in (4 weeks) and wash-out (3 months) periods. |
| Memantine PET [56] | FDG PET  SIB | CSF: Aβ40, Aβ42  CSF: phospho-tau and tau | X | ✓ | X | X | X | X | Imaging and CSF biomarkers. |
| Memantine MRI [57] | MRI: whole brain vol. | MRI: hippocampal vol. | X | X | X | X | ✓ | X | Imaging biomarker. |
| Memantine MRS [58] | MRS: NAA/Cr ratio in inferior parietal region | - | X | ✓ | X | X | X | X | Imaging biomarker. |
| Memantine vs. donepezil MRS [59] | MRS: NAA and NAA/Cr, Cho/Cr and MI/Cr ratios in the posterior cingulate gyrus, right temporal lobe, right frontal lobe and left occipital lobe | - | X | ✓ | X | X | X | X | Imaging biomarker. |
| Memantine multimodal [60] | FDG PET  MRI: hippocampal atrophy  MRI: whole brain vol.  MRS: NAA and MI | - | NR | NR | NR | NR | NR | NR | Imaging biomarkers. |
| REFLECT-1 [61] | ADAS-cog  CIBIC+ | - | ✓ | X | X | ✓ | ✓ | Multiple imputation methods and LOCF used to check results of mixed modelling of data with missing values. | Long-term follow-up. |
| Rosiglitazone genetics [62] | ADAS-cog  CIBIC+ | - | ✓ | X | X | ✓ | X | X | Long-term follow-up. |
| Azeliragon [63] | ADAS-cog | CSF: Aβ1-x, Aβ40, Aβ42  CSF: phospho-tau and total tau  MRI: hippocampal vol.  MRI: whole brain vol. | ✓ | Conducted in addition to separate analyses using LOCF, mixed modelling and data imputation. | X | ✓ | ✓ | Multiple imputation method used. | Long-term follow-up.  Imaging and CSF biomarkers. [n=52 for CSF, n=124 for MRI] |

**Key**

Under the heading ‘methods used to deal with deaths and drop-outs (primary outcome)’ a tick (✓) indicates that a given method was used and a cross (X) that it was not used or that the authors did not state it was used. Where ‘NR’ is present in all the boxes under this heading then a given study did not report any relevant information.

**Clinical rating scales**

ADAS-cog Alzheimer’s Disease Assessment Scale – cognitive subscale [64] [No distinction made between different versions]

ADCS-ADL Alzheimer’s Disease Cooperative Study – Activities of Daily Living inventory [65]

ADCS-CGIC Alzheimer’s Disease Cooperative Study Clinical - Global Impression of Change [66]

Blessed Dementia Scale Blessed Dementia Scale [67]

BNT Boston Naming Test [68]

BPRS Brief Psychiatric Rating Scale [69]

CDR-SB The Washington University Clinical Dementia Rating Sum-of-Boxes score [70]

CGIC Clinical Global Impression of Change [71]

CIBIC+ Clinician Interview-Based Impression of Change with Caregiver Input (ADCS version) [72]

CLOX Clock Drawing Test [73]

DAD Disability Assessment for Dementia [74]

DRS Dementia Rating Scale [75]

GDS Global Deterioration Scale [76]

Global CDR The Washington University Clinical Dementia Rating global score [70]

Gottfries-Bråne-Steen Scale Gottfries-Bråne-Steen Scale [77]

IDDD Interview for Deterioration in Daily living activities in Dementia [78]

MMSE Mini-Mental State Examination [79]

NPI Neuropsychiatric Inventory [80]

NTB Neuropsychological Test Battery [81]

SADAS-cog Standardised Alzheimer’s Disease Assessment Scale [82]

SIB Severe Impairment Battery [83]

Sternberg’s Memory Scanning Test Sternberg’s Memory Scanning Test[84]

Token Test Token Test [85]

**Biomarker modalities Protein and metabolites**

CSF Cerebrospinal Fluid Aβ Amyloid beta

EEG Electroencephalography Aβ40 Amyloid beta isomer, length 40 amino acids

MRI Magnetic Resonance Imaging Aβ42 Amyloid beta isomer, length 42 amino acids

MRS Magnetic Resonance Spectroscopy Cho/Cr ratio Choline/Creatine ratio

PET Positron Emission Tomography MI Myoinositol

SPECT Single Photon Emission Computed Tomography MI/Cr ratio Myoinositol/Creatine ratio

NAA N-acetylaspartate

NAA/Cr ratio N-acetylaspartate/Creatine ratio

**PET ligands**

AV-45 (E)-4-(2-(6-(2-(2-(2-18F-fluoroethoxy)ethoxy)ethoxy)pyridin-3-yl)vinyl)-N-methyl benzenamine

FDG [^18^F]-2-fluoro-2-deoxyglucose

PiB [^11^C]Pittsburgh compound B

**SPECT ligands**

HMPAO [^99m^Tc]-hexamethylpropylene amine oxidase

**Other**

ADLs Activities of Daily Living

ITT Intention-To-Treat analysis

LOCF Last Observation Carried Forward

SD Standard Deviation

vol. Volume

**References**

1. Gilman S, Koller M, Black RS, Jenkins L, Griffith SG, et al. Clinical effects of Abeta immunization (AN1792) in patients with AD in an interrupted trial. Neurology. 2005;64:1553-62.
2. Feldman HH, Doody RS, Kivipelto M, Sparks DL, Waters DD, Jones RW, et al. Randomized controlled trial of atorvastatin in mild to moderate Alzheimer disease: LEADe. Neurology. 2010;74:956-64.
3. Salloway S, Sperling R, Fox NC, Blennow K, Klunk W, Raskind M, et al. Two phase 3 trials of bapineuzumab in mild-to-moderate Alzheimer's disease. N Engl J Med. 2014;370:322-33.
4. Salloway S, Sperling R, Gilman S, Fox NC, Blennow K, Raskind M, et al. A phase 2 multiple ascending dose trial of bapineuzumab in mild to moderate Alzheimer disease. Neurology. 2009;73:2061-70.
5. A Study to Evaluate the Efficacy and Safety of MABT5102A in Patient With Mild to Moderate Alzheimer's Disease (ABBY). ClinicalTrials.gov. 2015. http://www.clinicaltrials.gov/ct2/show/NCT01343966. Accessed 9 Oct 2015.
6. Roche announces phase II clinical results of crenezumab in Alzheimers disease. Roche. 2014. http://www.roche.com/investors/updates/inv-update-2014-07-16.htm. Accessed 12 Oct 2015.
7. A Study to Evaluate the Impact of MABT5102A on Brain Amyloid Load and Related Biomarkers in Patients with Mild to Moderate Alzheimer's Disease. ClinicalTrials.gov. 2015. http://www.clinicaltrials.gov/ct2/show/NCT01397578. Accessed 9 Oct 2015.
8. AC Immune receives milestone payment for crenezumab moving into phase III clinical development in Alzheimer's disease. Swiss Biotech. 2015. http://www.swissbiotech.org/b/index.php?1=1&id=665679. Accessed 12 Oct 2015.
9. Salloway S, Sperling R, Keren R, Porsteinsson AP, van Dyck CH, Tariot PN, et al. A phase 2 randomized trial of ELND005, scyllo-inositol, in mild to moderate Alzheimer disease. Neurology. 2011;77:1253-62.
10. Effect of LY450139 on the Long Term Progression of Alzheimer's Disease. ClinicalTrials.gov. 2015. http://www.clinicaltrials.gov/ct2/show/NCT00594568. Accessed 9 Oct 2015.
11. Doody RS, Raman R, Farlow M, Iwatsubo T, Vellas B, Joffe S, et al. A phase 3 trial of semagacestat for treatment of Alzheimer's disease. N Engl J Med. 2013;369:341-50.
12. Effect of LY450139, on the progression of Alzheimer's disease as compared with placebo (IDENTITY-2). ClinicalTrials.gov. 2015. http://www.clinicaltrials.gov/ct2/show/NCT00762411. Accessed 22 Sep 2015.
13. Simons M, Schwarzler F, Lutjohann D, von BK, Beyreuther K, Dichgans J, et al. Treatment with simvastatin in normocholesterolemic patients with Alzheimer's disease: A 26-week randomized, placebo-controlled, double-blind trial. Ann Neurol. 2002;52:346-50.
14. Doody RS, Thomas RG, Farlow M, Iwatsubo T, Vellas B, Joffe S, et al. Phase 3 trials of solanezumab for mild-to-moderate Alzheimer's disease. N Engl J Med. 2014;370:311-21.
15. Green RC, Schneider LS, Amato DA, Beelen AP, Wilcock G, Swabb EA, et al. Effect of tarenflurbil on cognitive decline and activities of daily living in patients with mild Alzheimer disease: a randomized controlled trial. JAMA. 2009;302:2557-64.
16. Wilcock GK, Black SE, Hendrix SB, Zavitz KH, Swabb EA, Laughlin MA. Efficacy and safety of tarenflurbil in mild to moderate Alzheimer's disease: a randomised phase II trial. Lancet Neurol. 2008;7:483-93.
17. Aisen PS, Gauthier S, Ferris SH, Saumier D, Haine D, Garceau D, et al.Tramiprosate in mild-to-moderate Alzheimer's disease - a randomized, double-blind, placebo-controlled, multi-centre study (the Alphase Study). Arch Med Sci. 2011;7:102-11.
18. Molloy DW, Standish TI, Zhou Q, Guyatt G. A multicenter, blinded, randomized, factorial controlled trial of doxycycline and rifampin for treatment of Alzheimer's disease: the DARAD trial. Int J Geriatr Psychiatry. 2013;28:463-70.
19. Schneider L, Porsteinsson A, Farlow M, Shimakura A, Nakagawa M, Iwakami N. The neuroprotective and neurotrophic agent T-817MA for Alzheimer's disease: Randomized, double-blind, placebo-controlled proof-of-concept trial outcomes. Alzheimers Dement. 2013;9:530-1.
20. Soininen H, West C, Robbins J, Niculescu L: Long-term efficacy and safety of celecoxib in Alzheimer's disease. Dement Geriatr Cogn Disord. 2007;23:8-21.
21. Alzheimer disease: phase 2 trial results reported by Immune Network Ltd. The Free Library. 2002. http://www.thefreelibrary.com/Alzheimer%20Disease:%20Phase%202%20Trial%20Results%20Reported%20by%20Immune%20Network...-a092852880. Accessed 22 Sep 2015.
22. Scharf S, Mander A, Ugoni A, Vajda F, Christophidis N. A double-blind, placebo-controlled trial of diclofenac/misoprostol in Alzheimer's disease. Neurology. 1999;53:197-1.
23. Quinn JF, Raman R, Thomas RG, Yurko-Mauro K, Nelson EB, van DC, et al. Docosahexaenoic acid supplementation and cognitive decline in Alzheimer disease: a randomized trial. JAMA. 2010;304:1903-11.
24. Freund-Levi Y, Eriksdotter-Jonhagen M, Cederholm T, Basun H, Faxen-Irving G, Garlind A, et al. Omega-3 fatty acid treatment in 174 patients with mild to moderate Alzheimer disease: OmegAD study: a randomized double-blind trial. Arch Neurol. 2006;63:1402-8.
25. Lee DY, Kim KW, Jhoo JH, Ryu S, Choo IH, Seo EH, at al. A multicenter, randomized, placebo-controlled, double-blind clincial trial of escitalopram on its atrophy-delaying effect in Alzheimer's disease. Alzheimers Dement. 2012;8:603.
26. Van Gool WA, Weinstein HC, Scheltens P, Walstra GJ. Effect of hydroxychloroquine on progression of dementia in early Alzheimer's disease: an 18-month randomised, double-blind, placebo-controlled study. Lancet. 2001;358:455-460.
27. Pasqualetti P, Bonomini C, Dal FG, Paulon L, Sinforiani E, Marra C, et al. A randomized controlled study on effects of ibuprofen on cognitive progression of Alzheimer's disease. Aging Clin Exp Res. 2009;21:102-10.
28. de JD, Jansen R, Hoefnagels W, Jellesma-Eggenkamp M, Verbeek M, Borm G, et al. No effect of one-year treatment with indomethacin on Alzheimer's disease progression: a randomized controlled trial. PLoS One. 2008;3:e1475.
29. Rogers J, Kirby LC, Hempelman SR, Berry DL, McGeer PL, Kaszniak AW, et al. Clinical trial of indomethacin in Alzheimer's disease. Neurology. 1993;43:1609-1611.
30. Piette F, Belmin J, Vincent H, Schmidt N, Pariel S, Verny M, et al. Masitinib as an adjunct therapy for mild-to-moderate Alzheimer's disease: a randomised, placebo-controlled phase 2 trial. Alzheimers Res Ther. 2011;3:16.
31. Aisen PS, Davis KL, Berg JD, Schafer K, Campbell K, Thomas RG, et al. A randomized controlled trial of prednisone in Alzheimer's disease. Alzheimer's Disease Cooperative Study. Neurology. 2000;54:588-593.
32. Turner RS, Thomas RG, Craft S, van Dyck CH, Mintzer J, Reynolds BA, et al. A randomized, double-blind, placebo-controlled trial of resveratrol for Alzheimer disease. Neurology. 2015. doi:10.1212/WNL.0000000000002035.
33. Reines SA, Block GA, Morris JC, Liu G, Nessly ML, Lines CR, et al. Rofecoxib: no effect on Alzheimer's disease in a 1-year, randomized, blinded, controlled study. Neurology. 2004;62:66-71.
34. Aisen PS, Schafer KA, Grundman M, Pfeiffer E, Sano M, Davis KL, et al. Effects of rofecoxib or naproxen vs placebo on Alzheimer disease progression: a randomized controlled trial. JAMA. 2003;289:2819-2826.
35. Cucinotta D, De Leo D, Frattola L, Trabucchi M, Albizatti M, Beltramelli A, et al. Dihydroergokryptine as long-term treatment of Alzheimer type dementia: a multicenter two-year follow-up. Arch Gerontol Geriatr. 1998;Suppl 6:103-10.
36. Remington R, Bechtel C, Larsen D, Samar A, Doshanjh L, Fishman P, et al. A Phase II Randomized Clinical Trial of a Nutritional Formulation for Cognition and Mood in Alzheimer's Disease. J Alzheimers Dis. 2015;45:395-405.
37. Filip V, Kolibas E. Selegiline in the treatment of Alzheimer's disease: a long-term randomized placebo-controlled trial. Czech and Slovak Senile Dementia of Alzheimer Type Study Group. J Psychiatry Neurosci. 1999;24:234-43.
38. Freedman M, Rewilak D, Xerri T, Cohen S, Gordon AS, Shandling M, et al. L-deprenyl in Alzheimer's disease: cognitive and behavioral effects. Neurology. 1998;50:660-668.
39. Koivisto K, Helkala E-L, Hanninen T, Vanhanen M, Aaltonen H, Reinikainen K, et al. Three-year follow-up of long-term selegiline treatment of Alzheimer's disease. J Neurol. 1995;242:S34-S35.
40. Burke WJ, Roccaforte WH, Wengel SP, Bayer BL, Ranno AE, Willcockson NK. L-deprenyl in the treatment of mild dementia of the Alzheimer type: results of a 15-month trial. J Am Geriatr Soc. 1993;41:1219-25.
41. Sano M, Ernesto C, Thomas RG, Klauber MR, Schafer K, Grundman M, et al. A controlled trial of selegiline, alpha-tocopherol, or both as treatment for Alzheimer's disease. The Alzheimer's Disease Cooperative Study. New Engl J Med. 1997;336:1216-1222.
42. Tariot PN, Schneider LS, Cummings J, Thomas RG, Raman R, Jakimovich LJ, et al. Alzheimer's Disease Cooperative Study Group. Chronic divalproex sodium to attenuate agitation and clinical progression of Alzheimer disease. Arch Gen Psychiatry 2011;68:853-61.
43. Wischik CM, Staff RT, Wischik DJ, Bentham P, Murray AD, Storey JM, et al. Tau aggregation inhibitor therapy: an exploratory phase 2 study in mild or moderate Alzheimer's disease. J Alzheimers Dis. 2015;44:705-20.
44. Krishnan KR, Charles HC, Doraiswamy PM, Mintzer J, Weisler R, Yu X, et al. Randomized, placebo-controlled trial of the effects of donepezil on neuronal markers and hippocampal volumes in Alzheimer's disease. Am J Psychiatry. 2003;160:2003-11.
45. Burns A, Rossor M, Hecker J, Gauthier S, Petit H, Moller HJ, et al. The effects of donepezil in Alzheimer's disease - results from a multinational trial. Dement Geriatr Cogn Disord. 1999;10:237-44.
46. Rogers SL, Farlow MR, Doody RS, Mohs R, Friedhoff LT. A 24-week, double-blind, placebo-controlled trial of donepezil in patients with Alzheimer's disease. Donepezil Study Group. Neurology. 1998;50:136-45.
47. Raskind MA, Peskind ER, Wessel T, Yuan W. Galantamine in AD: A 6-month randomized, placebo-controlled trial with a 6-month extension. The Galantamine USA-1 Study Group. Neurology. 2000;54:2261-68.
48. A Phase 3 Study Evaluating Safety and Effectiveness of Immune Globulin Intravenous (IGIV 10%) for the Treatment of Mild-to-Moderate Alzheimer's Disease. ClinicalTrials.gov. 2015. http://www.clinicaltrials.gov/ct2/show/NCT00818662. Accessed 9 Oct 2015.
49. Relkin N. Results of the GAP 160701 study: A phase 3 clinical trial of intravenous immunoglobulin for mild-to-moderate Alzheimer's disease. Alzheimers Dement. 2013;9:530.
50. Safety and Efficacy Study Evaluating Dimebon in Patients With Mild to Moderate Alzheimer's Disease on Donepezil (CONCERT). ClinicalTrials.gov. 2012. http://www.clinicaltrials.gov/ct2/show/NCT00829374. Accessed 9 Oct 2015.
51. Sweetlove M. Phase III CONCERT Trial of Latrepirdine. Pharm Med. 2012;26:113-5.
52. A Safety and Efficacy Study of Oral Dimebon in Patients With Mild-To-Moderate Alzheimer's Disease (CONNECTION). ClinicalTrials.gov. 2012. http://www.clinicaltrials.gov/ct2/show/NCT00675623. Accessed 9 Oct 2015.
53. Pfizer And Medivation Announce Results From Two Phase 3 Studies In Dimebon (latrepirdine*) Alzheimer's Disease Clinical Development Program. Pfizer. 2010. http://press.pfizer.com/press-release/pfizer-and-medivation-announce-results-two-phase-3-studies-dimebon-latrepirdine-alzhei. Accessed 12 Oct 2015.
54. Doody RS, Gavrilova SI, Sano M, Thomas RG, Aisen PS, Bachurin SO, et al. Effect of dimebon on cognition, activities of daily living, behaviour, and global function in patients with mild-to-moderate Alzheimer's disease: a randomised, double-blind, placebo-controlled study. Lancet. 2008;372:207-15.
55. Alvarez XA, Cacabelos R, Laredo M, Couceiro V, Sampedro C, Varela M, et al. A 24-week, double-blind, placebo-controlled study of three dosages of Cerebrolysin in patients with mild to moderate Alzheimer's disease. Eur J Neurol. 2006;13:43-54.
56. Wang T, Huang Q, Reiman EM, Chen K, Li X, Li G, et al. Effects of memantine on clinical ratings, fluorodeoxyglucose positron emission tomography measurements, and cerebrospinal fluid assays in patients with moderate to severe Alzheimer dementia: a 24-week, randomized, clinical trial. J Clin Psychopharmacol. 2013;33:636-42.
57. Wilkinson D, Fox NC, Barkhof F, Phul R, Lemming O, Scheltens P. Memantine and brain atrophy in Alzheimer's disease: a 1-year randomized controlled trial. J Alzheimers Dis. 2012;29:459-69.
58. Ashford JW, Adamson M, Beale T, La D, Hernandez B, Noda A, et al. MR spectroscopy for assessment of memantine treatment in mild to moderate Alzheimer dementia. J Alzheimers Dis. 2011;26 Suppl 3:331-6.
59. Modrego PJ, Fayed N, Errea JM, Rios C, Pina MA, Sarasa M. Memantine versus donepezil in mild to moderate Alzheimer's disease: A randomized trial with magnetic resonance spectroscopy. Eur J Neurol. 2010;17:405-12.
60. Schmidt R, Ropele S, Pendl B, Ofner P, Enzinger C, Schmidt H, et al. Longitudinal multimodal imaging in mild to moderate Alzheimer disease: a pilot study with memantine. J Neurol Neurosurg Psychiatry. 2008;79:1312-7.
61. Gold M, Alderton C, Zvartau-Hind M, Egginton S, Saunders AM, Irizarry M, et al. Rosiglitazone monotherapy in mild-to-moderate Alzheimer's disease: results from a randomized, double-blind, placebo-controlled phase III study. Dement Geriatr Cogn Disord. 2010;30:131-46.
62. Risner ME, Saunders AM, Altman JF, Ormandy GC, Craft S, Foley IM, et al. Efficacy of rosiglitazone in a genetically defined population with mild-to-moderate Alzheimer's disease. Pharmacogenomics J. 2006;6:246-54.
63. Galasko D, Bell J, Mancuso JY, Kupiec JW, Sabbagh MN, van DC, et al. Clinical trial of an inhibitor of RAGE-Abeta interactions in Alzheimer disease. Neurology. 2014;82:1536-42.
64. Mohs RC, Knopman D, Petersen RC, Ferris SH, Ernesto C, Grundman M, et al. Development of cognitive instruments for use in clinical trials of antidementia drugs: additions to the Alzheimer's Disease Assessment Scale that broaden its scope. The Alzheimer's Disease Cooperative Study. Alzheimer Dis Assoc Disord. 1997;11:S13-S21.
65. Galasko D, Bennett D, Sano M, Ernesto C, Thomas R, Grundman M, et al. An inventory to assess activities of daily living for clinical trials in Alzheimer's disease. The Alzheimer's Disease Cooperative Study. Alzheimer Dis Assoc Disord. 1997;11:S33-S39.
66. Schneider LS, Olin JT, Doody RS, Clark CM, Morris JC, Reisberg B, et al. Validity and reliability of the Alzheimer's Disease Cooperative Study-Clinical Global Impression of Change. The Alzheimer's Disease Cooperative Study. Alzheimer Dis Assoc Disord. 1997;11:S22-S32.
67. Blessed G, Tomlinson BE, Roth M. The association between quantitative measures of dementia and of senile change in the cerebral grey matter of elderly subjects. Br J Psychiatry. 1968;114:797-811.
68. Kaplan E, Goodglass H, Weintraub S. The Boston Naming Test. Philadelphia: Lea & Febiger; 1983.
69. Overall JE, Beller SA. The Brief Psychiatric Rating Scale (BPRS) in geropsychiatric research: I. Factor structure on an inpatient unit. J Gerontol. 1984;39:187-93.
70. Morris JC. The Clinical Dementia Rating (CDR): current version and scoring rules. Neurology. 1993;43:2412-4.
71. Rockville MD. Clinical global impressions. In ECDEU assessment manual for psychopharmacology. Revised. edition. Edited by Guy W: US Department of Health Education and Welfare; 1976. p218-22.
72. Knopman DS, Knapp MJ, Gracon SI, Davis CS. The Clinician Interview-Based Impression (CIBI): a clinician's global change rating scale in Alzheimer's disease. Neurology. 1994;44:2315-21.
73. Shulman K, Shedletsky R, Silver I. The challenge of time: clock drawing and cognitive function in the elderly. Int J Geriatr Psychiatry. 1986;1:135-40.
74. Gelinas I, Gauthier L, McIntyre M, Gauthier S. Development of a functional measure for persons with Alzheimer's disease: the disability assessment for dementia. Am J Occup Ther. 1999;53:471-81.
75. Monsch AU, Bondi MW, Salmon DP, Butters N, Thal LJ, Hansen LA, et al. Clinical validity of the Mattis Dementia Rating Scale in detecting Dementia of the Alzheimer type. A double cross-validation and application to a community-dwelling sample. Arch Neurol. 1995;52:899-904.
76. Reisberg B, Ferris SH, de Leon MJ, Crook T. The Global Deterioration Scale for assessment of primary degenerative dementia. Am J Psychiatry. 1982;139:1136-9.
77. Gottfries CG, Brane G, Gullberg B, Steen G. A new rating scale for dementia syndromes. Arch Gerontol Geriatr. 1982;1:311-30.
78. Teunisse S, Derix MM. The interview for deterioration in daily living activities in dementia: agreement between primary and secondary caregivers. Int Psychogeriatr. 1997;9:155-62.
79. Folstein MF, Folstein SE, McHugh PR. "Mini-mental state". A practical method for grading the cognitive state of patients for the clinician. J Psychiatr Res. 1975;12:189-98.
80. Cummings JL, Mega M, Gray K, Rosenberg-Thompson S, Carusi DA, Gornbein J. The Neuropsychiatric Inventory: comprehensive assessment of psychopathology in dementia. Neurology. 1994;44:2308-14.
81. Harrison J, Minassian SL, Jenkins L, Black RS, Koller M, Grundman M. A neuropsychological test battery for use in Alzheimer disease clinical trials. Arch Neurol 2007;64:1323-9.
82. Standish TI, Molloy DW, Bedard M, Layne EC, Murray EA, Strang D. Improved reliability of the Standardized Alzheimer's Disease Assessment Scale (SADAS) compared with the Alzheimer's Disease Assessment Scale (ADAS). J Am Geriatr Soc. 1996;44:712-716.
83. Saxton J, Swihart AA. Neuropsychological assessment of the severely impaired elderly patient. Clin Geriatr Med. 1989;5:531-543.
84. Sternberg S. High-speed scanning in human memory. Science. 1966;153:652-4.
85. Brookshire RH. A token test battery for testing auditory comprehension in brain-injured adults. Brain Lang. 1978;6:149-57.
