# Supplementary material for: A review of clinical trial designs used to detect a disease-modifying effect of drug therapy in Alzheimer’s disease and Parkinson’s disease
Source: BMC Neurol. 2016 Jun 16;16:92. doi: 10.1186/s12883-016-0606-3 (PMC4910262; doi:10.1186/s12883-016-0606-3)
Supplement: Additional file 10: — Key design features and outcome measures in published PD RCTs. (DOCX 50 kb) [file 12883_2016_606_MOESM10_ESM.docx]

**Additional file 10: Key design features and outcome measures used in published randomised controlled trials of putative disease-modifying agents in Parkinson’s disease**

| **Trial** | **Primary outcome measures** | **Biomarkers or time-to-event outcomes used as secondary outcome measures** | **Methods used to deal with deaths and drop-outs (primary outcome)** | | | | | | **Methods used to differentiate symptomatic from disease-modifying effects of the agent** |
| --- | --- | --- | --- | --- | --- | --- | --- | --- | --- |
|  |  |  | **ITT** | **Complete-case analysis** | **Survival analysis** | **LOCF** | **Mixed model** | **Data**  **imputation** |  |
| PRECEPT[1] | Time to dopaminergic treatment | [^123^I]β-CIT SPECT: striatal uptake | ✓ | X | ✓ | X | X | X | Long-term follow-up. Time-to-event outcome.  Imaging biomarker.  Analysis of secondary outcomes after wash-out (4 weeks after receiving experimental treatment or placebo for ≥ 24 months) period. |
| Green tea [2, 3] | Total UPDRS | - | NR | NR | NR | NR | NR | NR | Randomised delayed-start design: the group initially treated with placebo commenced active treatment after 6 months. |
| TCH346 [4] | Time to dopaminergic treatment | - | ✓ | X | ✓ | X | X | X | Long-term follow-up. Time-to-event outcome.  Analysis of secondary outcomes after wash-in (4 weeks) and wash-out (4 weeks after reaching end-point or 12-18 months) periods. |
| Exenatide [5] | UPDRS (III) | [^123^I]FP-CIT SPECT: putamen, caudate and striatal uptake | ✓ | X | X | ✓ | X | X | Analysis of primary outcome after wash-out period (8 weeks)  Imaging biomarker.  [n=10 for SPECT] |
| H_2_-water [6] | Total UPDRS | - | X | ✓ | X | X | X | X | Analysis of primary and secondary outcomes after wash-out (8 weeks) period. |
| ROADS [7] | Time to levodopa treatment | - | ✓ | X | ✓ | X | X | X | Long-term follow-up. Time-to-event outcome.  Staggered wash-out analysis of secondary outcome measures. Whilst the primary outcome was analysed after 52 weeks, patients were randomised to receive active-agent for 52 or 54 weeks and then placebo for 4 or 2 weeks (placebo of predetermined duration also given if endpoint reached earlier).  Also wash-in (4 and 13 weeks) analyses. |
| Subtherapeutic pergolide [8] | Time to levodopa treatment | - | ✓ | X | ✓ | X | X | X | Long-term follow-up. Time-to-event outcome.  Analysis of secondary outcomes after wash-in (6 weeks) and wash-out (4 weeks after end-point) periods. |

| **Trial** | | **Primary outcome measures** | **Biomarkers or time-to-event outcomes used as secondary outcome measures** | | **Methods used to deal with deaths and drop-outs (primary outcome)** | | | | | | | | | | | **Methods used to differentiate symptomatic from disease-modifying effects of the agent** |
| --- | --- | --- | --- | --- | --- | --- | --- | --- | --- | --- | --- | --- | --- | --- | --- | --- |
|  |  |  |  |  | **ITT** | | **Complete-case analysis** | **Survival analysis** | | **LOCF** | | **Mixed model** | | **Data**  **imputation** | |  |
| CALM-PD-CIT [9] | | [^123^I]β-CIT SPECT: striatal uptake | [^123^I]β-CIT SPECT: putamen and caudate uptake | | ✓ | | Conducted two analyses; one only using available data and the other using data imputation. | X | | X | | X | | Regression models, derived from those with complete data, used to impute missing values. | | Long-term follow-up.  Imaging biomarker. |
| Rasagiline cognition [10] | | RAVLT | - | | NR | | NR | NR | | NR | | NR | | NR | | Long-term follow-up. |
| Russian rasagiline [11] | | UPDRS (III) | - | | NR | | NR | NR | | NR | | NR | | NR | | Analysis of primary outcome after wash-out (4 weeks) period. |
| ADAGIO [12] | | Total UPDRS:  (1) Rate of change per week (weeks 12-36)  (2) Change from baseline to week 72  (3) Rate of change per week (weeks 48-72) | - | | ✓ | | X | X | | X | | ✓ | | Various sensitivity and supportive analyses, including multiple imputation strategies, were used to validate the results and address the issue of missing data. | | Long-term follow-up.  Randomised delayed-start design: the group initially treated with placebo commenced active treatment after 36 weeks.  Subjects could enter the active phase before 36 weeks if they required additional therapy.  Hierarchical primary outcomes designed to demonstrate: (1) rasagiline, compared to placebo, slowed disease progression; (2) early-start treatment of superior benefit over the whole study period, indicating disease-modification; (3) non-inferiority of early-start treatment by showing an enduring, non-diminishing (and thus likely due to a symptomatic effect) difference between the groups. |
| TEMPO [13] | | Total UPDRS | Time to dopaminergic treatment | | ✓ | | X | X | | ✓ | | X | | X | | Long-term follow-up.  Randomised delayed-start design: the group initially treated with placebo commenced active treatment after 6 months.  Subjects could enter the active phase before 6 months if it was determined that they required additional dopaminergic therapy earlier.  Time-to-event outcome. |
| **Trial** | **Primary outcome measures** | | **Biomarkers or time-to-event outcomes used as secondary outcome measures** | **Methods used to deal with deaths and drop-outs (primary outcome)** | | | | | | | | | | | | **Methods used to differentiate symptomatic from disease-modifying effects of the agent** |
|  |  |  |  | **ITT** | | **Complete-case analysis** | | | **Survival analysis** | | **LOCF** | | **Mixed model** | | **Data**  **imputation** |  |
| REAL-PET [14] | FDOPA PET: putamen uptake | | Time to dyskinesias | ✓ | | Those withdrawing after 12 months could attend for a final PET scan and were still included in the analysis. | | | X | | X | | X | | X | Long-term follow-up.  Imaging biomarker.  Time-to-event outcome. |
| UK/France  FDOPA PET [15] | FDOPA PET: putamen uptake | | - | ✓ | | ✓ | | | X | | X | | X | | X | Imaging biomarker. |
| Norwegian-Danish [16] | Levodopa requirement  Total UPDRS  UPDRS (III) | | Time to development of motor fluctuations | ✓ | | X | | | X | | X | | ✓ | | X | Long-term follow-up.  Analysis of primary outcomes after wash-out (4 weeks) period.  Time-to-event outcome. |
| SELEDO [17] | Time until baseline levodopa dose had to be increased by ≥ 50% | | - | ✓ | | X | | | ✓ | | X | | X | | X | Long-term follow-up.  Time-to-event outcome. |
| Swedish selegiline [18] | Time to levodopa treatment | | - | X | | X | | | ✓ | | X | | X | | X | Time-to-event outcome.  Long-term follow-up study, continued until all included patients reached primary endpoint.  Analysis of secondary outcomes after wash-in (6 and 12 weeks) and wash-out (8 weeks after reaching end-point) periods. |
| SINDEPAR [19] | Total UPDRS | | - | X | | ✓ | | | X | | X | | X | | X | Analysis or primary outcome after wash-in (12 weeks) and wash-out (8 weeks after 12 months of follow-up) periods. |
| Finnish selegiline [20] | Time to levodopa treatment | | - | X | | X | | | ✓ | | X | | X | | X | Time-to-event outcome.  Long-term follow-up study, continued until all included patients reached primary endpoint.  Analysis or secondary outcomes after wash-in (4 and 8 weeks) periods. |
| Tetrud and Langston [21] | Time to levodopa treatment | | - | X | | X | | | ✓ | | X | | X | | X | Long-term follow-up.  Time-to-event outcome.  Analysis of secondary outcomes after wash-in (4 weeks) and wash-out (4 weeks after reaching end-point or 36 months) periods. |

| **Trial** | **Primary outcome measures** | **Biomarkers or time-to-event outcomes used as secondary outcome measures** | **Methods used to deal with deaths and drop-outs (primary outcome)** | | | | | | **Methods used to differentiate symptomatic from disease-modifying effects of the agent** |
| --- | --- | --- | --- | --- | --- | --- | --- | --- | --- |
|  |  |  | **ITT** | **Complete-case analysis** | **Survival analysis** | **LOCF** | **Mixed model** | **Data**  **imputation** |  |
| DATATOP [22] | Time to levodopa treatment | - | ✓ | X | ✓ | X | X | X | Long-term follow-up.  Time-to-event outcome.  Analysis of secondary outcomes after wash-in (4 and 12 weeks) and wash-out (4 and 8 weeks after reaching end-point or 24 months) periods. |
| QE3 [23] | Total UPDRS | Time to dopaminergic treatment | X | X | X | ✓ | X | X | Long-term follow-up study.  Time-to-event outcome. |
| QE2 [24] | Total UPDRS | Time to levodopa treatment | ✓ | X | X | ✓ | X | X | Long-term follow-up study.  Analysis of primary and secondary outcomes after wash-in (4 weeks) period.  Time-to-event outcome. |
| Creatine-CoQ10 [25] | MoCA  Plasma phospholipids UPDRS (III) | - | NR | NR | NR | NR | NR | NR | Long-term follow-up study.  Blood biomarker. |
| NET-PD LS-1 Creatine [26] | Global outcome measure | - | ✓ | X | X | X | ✓ | Worst values imputed for those dying prior to 5 years. Other data imputed by a multivariate method. | Long-term follow-up. |
| German Creatine [27] | [^123^I]FP-CIT SPECT: striatal uptake | - | NR | NR | NR | NR | NR | NR | Imaging biomarker. |
| Fenugreek [28] | Total UPDRS and subsections | - | X | ✓ | X | X | X | X | Long-term follow-up study. |
| Ubiquinol-10 [29] | Total UPDRS | Time to levodopa treatment | X | ✓ | X | X | X | X | Long-term follow-up study  Analysis of primary and secondary outcomes after wash-in (8 weeks) and wash-out (8 weeks after 48 or 96 weeks depending on group allocation) periods.  Time-to-event outcome. |

| **Trial** | **Primary outcome measures** | **Biomarkers or time-to-event outcomes used as secondary outcome measures** | **Methods used to deal with deaths and drop-outs (primary outcome)** | | | | | | **Methods used to differentiate symptomatic from disease-modifying effects of the agent** |
| --- | --- | --- | --- | --- | --- | --- | --- | --- | --- |
|  |  |  | **ITT** | **Complete-case analysis** | **Survival analysis** | **LOCF** | **Mixed model** | **Data**  **imputation** |  |
| FAIRPARK [30] | MRI: substantia nigra R2*  UPDRS (III) | - | X | ✓ | X | X | X | X | Randomised delayed-start design: the group initially treated with placebo commenced active treatment after 6 months.  Hierarchical primary outcomes designed to demonstrate: (1) deferiprone, compared to placebo, slowed disease progression; (2) early-start treatment had a superior benefit over the whole study period, indicating a disease-modifying effect; (3) non-inferiority of early-start treatment by showing an enduring, rather than diminishing (and thus likely due to a symptomatic effect) difference between the groups  Imaging biomarker. |
| ELLDOPA [31] | Total UPDRS | [^123^I]β-CIT SPECT: striatal uptake | ✓ | ✓ | X | X | X | X | Long-term follow-up.  Analysis of primary outcome at 42 weeks after a 2 week wash-out period.  Imaging biomarker. [n=142 for SPECT] |
| PROUD [32] | Total UPDRS | [^123^I]FP-CIT SPECT: striatal uptake | X | X | X | ✓ | X | X | Randomised delayed-start design: the group initially treated with placebo commenced active treatment after 6-9 months.  Imaging biomarker. |
| GM1 ganglioside [33] | UPDRS (III) | [^11^C]MP PET: striatal uptake  (sub-study published separately)[34] | ✓ | X | X | X | ✓ | Assessed impact of missing data using multiple imputation method | Long-term follow-up.  Randomised delayed-start design: the group initially treated with placebo commenced active treatment after 6 months.  Analysis of primary outcome after wash-out (1 and 2 years) periods.  Imaging biomarker.  [n=29 for [^11^C]MP PET] |
| MitoQ trial [35] | Total UPDRS | Time to dopaminergic treatment | X | X | X | ✓ | X | X | Long-term follow-up. Time-to-event outcome.  Analysis of primary outcome after wash-in (4 weeks) period. |

| **Trial** | **Primary outcome measures** | **Biomarkers or time-to-event outcomes used as secondary outcome measures** | **Methods used to deal with deaths and drop-outs (primary outcome)** | | | | | | **Methods used to differentiate symptomatic from disease-modifying effects of the agent** |
| --- | --- | --- | --- | --- | --- | --- | --- | --- | --- |
|  |  |  | **ITT** | **Complete-case analysis** | **Survival analysis** | **LOCF** | **Mixed model** | **Data**  **imputation** |  |
| Riluzole international [36] | Time to dopaminergic treatment | FDOPA PET: striatal uptake | ✓ | X | ✓ | X | X | X | Time-to-event outcome.  Analysis of total UPDRS (secondary outcome measure) after wash-out (8 weeks) period. |
| Riluzole USA [37] | UPDRS (II)  UPDRS (III) | Time to levodopa treatment | ✓ | ✓ | X | X | X | X | Long-term follow-up.  Time-to-event outcome.  Analysis of primary outcomes after wash-out (6 weeks) period at 6 months, before open-label extension until one-year.  Open-label extension essentially gave the study a randomised delayed-start design in which the group initially treated with placebo commenced active treatment after 6 months. |
| GPI-1485  (6 month trial) [38] | [^123^I]β-CIT SPECT: striatal uptake  UPDRS (III) | - | NR | NR | NR | NR | NR | NR | Imaging biomarker. |

**Key**

Under the heading ‘methods used to deal with deaths and drop-outs (primary outcome)’ a tick (✓) indicates that a given method was used and a cross (X) that it was not used or that the authors did not state it was used. Where ‘NR’ is present in all the boxes under this heading then a given study did not report any relevant information.

**Clinical rating scales**

Global outcome measure Composite measure comprising the Schwab and England activities of daily living scale [39], Unified Parkinson’s Disease Rating Scale, questions related to ambulatory capacity, the 39-item Parkinson’s disease questionnaire (PDQ-39) [40], the Symbol Digit Modalities Test [41] and the Modified Rankin Test [42].

RAVLT Rey Auditory Verbal Learning Test [43]

MoCA Montreal Cognitive Assessment [44]

Total UPDRS Total score derived from the Unified Parkinson’s Disease Rating Scale [45]

UPDRS (I) Mentation component of the Unified Parkinson’s Disease Rating Scale

UPDRS (III) Motor component of Unified Parkinson’s Disease Rating Scale

**Biomarker modalities Other**

MRI Magnetic Resonance Imaging ITT Intention-To-Treat analysis

PET Positron Emission Tomography LOCF Last Observation Carried Forward

SPECT Single-Photon Emission Computed Tomography

**MRI measurements**

R2* Proton transverse relaxation rate

**PET ligands**

FDOPA [^18^F]6-fluoro-L-3,4-dihydroxyphenylalanine

**SPECT ligands**

[^123^I]FP-CIT [^123^I]-2β-carbomethoxy-3β-(4-iodophenyl)-N-(3-fluoropropyl)-N-tropane

[^123^I]β-CIT [^123^I]-2β-carbomethoxy-3β-(4-iodophenyl tropane)

**References**

1. Parkinson Study Group. Mixed lineage kinase inhibitor CEP-1347 fails to delay disability in early Parkinson disease. Neurology. 2007;69:1480-90.
2. Efficacy and safety of green tea polyphenol in de novo Parkinson's disease patients. ClinicalTrials.gov. 2011. http://www.clinicaltrials.gov/ct2/show/NCT00461942. Accessed 22 Sep 2015.
3. Ability to Slow Disease Progression and Safety and Tolerability of Green Tea Polyphenols in Early Parkinson's Disease. The Michael J.Fox Foundation for Parkinson's Research. 2013. https://www.michaeljfox.org/foundation/grant-detail.php?grant_id=187. Accessed 22 Sep 2015.
4. Olanow CW, Schapira AH, Lewitt PA, Kieburtz K, Sauer D, Olivieri G, et al. TCH346 as a neuroprotective drug in Parkinson's disease: a double-blind, randomised, controlled trial. Lancet Neurol. 2006;5:1013-20.
5. Aviles-Olmos I, Dickson J, Kefalopoulou Z, Djamshidian A, Ell P, Soderlund T, et al. Exenatide and the treatment of patients with Parkinson's disease. J Clin Invest. 2013;123:2730-6.
6. Yoritaka A, Takanashi M, Hirayama M, Nakahara T, Ohta S, Hattori N. Pilot study of H_2_ therapy in Parkinson's disease: A randomized double-blind placebo-controlled trial. Mov Disord. 2013;28:836-9.
7. The Parkinson Study Group. Effect of lazabemide on the progression of disability in early Parkinson's disease. Ann Neurol. 1996;40:99-107.
8. Grosset K, Grosset D, Lees A, Parkinson's Disease Research Group of the United Kingdom. Trial of subtherapeutic pergolide in de novo Parkinson's disease. Mov Disord. 2005;20:363-6.
9. Parkinson Study Group. Dopamine transporter brain imaging to assess the effects of pramipexole vs levodopa on Parkinson disease progression. JAMA. 2002;287:1653-61.
10. The Effect of Rasagiline on Cognition in Parkinson's Disease. ClinicalTrials.gov. 2015. http://www.clinIcaltrials.gov/ct2/show/NCT01382342. Accessed 9 Oct 2015.
11. Illarioshkin S, Karabanov A, Mirkasimov A, Verejutina I. Rasagiline in drug-nave Russian patients with early Parkinson's disease. Mov Disord. 2012;27:380.
12. Olanow CW, Rascol O, Hauser R, Feigin PD, Jankovic J, Lang A, et al. A double-blind, delayed-start trial of rasagiline in Parkinson's disease. N Eng J Med. 2009;361:1268-78.
13. Parkinson Study Group. A controlled, randomized, delayed-start study of rasagiline in early Parkinson disease. Arch Neurol. 2004;61:561-6.
14. Whone AL, Watts RL, Stoessl AJ, Davis M, Reske S, Nahmias C, et al. Slower progression of Parkinson's disease with ropinirole versus levodopa: The REAL-PET study. Ann Neurol. 2003;54:93-101.
15. Rakshi JS, Pavese N, Uema T, Ito K, Morrish PK, Bailey DL, et al. A comparison of the progression of early Parkinson's disease in patients started on ropinirole or L-dopa: an 18F-dopa PET study. J Neural Transm. 2002;109:1433-43.
16. Larsen JP, Boas J, Erdal JE. Does selegiline modify the progression of early Parkinson's disease? Results from a five-year study. The Norwegian-Danish Study Group. Eur J Neurol. 1999;6:539-547.
17. Przuntek H, Conrad B, Dichgans J, Kraus PH, Krauseneck P, Pergande G, et al. SELEDO: a 5-year long-term trial on the effect of selegiline in early Parkinsonian patients treated with levodopa. Eur J Neurol. 1999;6:141-150.
18. Palhagen S, Heinonen EH, Hagglund J, Kaugesaar T, Kontants H, Maki-Ikola O, et al. Selegiline delays the onset of disability in de novo parkinsonian patients. Swedish Parkinson Study Group. Neurology 1998;51:520-5.
19. Olanow CW, Hauser RA, Gauger L, Malapira T, Koller W, Hubble J, et al. The effect of deprenyl and levodopa on the progression of Parkinson's disease. Ann Neurol. 1995;38:771-7.
20. Myllyla VV, Sotaniemi KA, Vuorinen JA, Heinonen EH. Selegiline as initial treatment in de novo parkinsonian patients. Neurology. 1992;42:339-43.
21. Tetrud JW, Langston JW. The effect of deprenyl (selegiline) on the natural history of Parkinson's disease. Science. 1989;245:519-22.
22. The Parkinson Study Group. Effects of tocopherol and deprenyl on the progression of disability in early Parkinson's disease. N Eng J Med. 1993;328:176-83.
23. Beal MF, Oakes D, Shoulson I, Henchcliffe C, Galpern WR, Haas R, et al. A randomized clinical trial of high-dosage coenzyme Q10 in early Parkinson disease: no evidence of benefit. JAMA Neurol. 2014;71:543-52.
24. Shults CW, Oakes D, Kieburtz K, Beal MF, Haas R, Plumb S, et al. Effects of coenzyme Q10 in early Parkinson disease: evidence of slowing of the functional decline. Arch Neurol. 2002;59:1541-50.
25. Li Z, Wang P, Yu Z, Cong Y, Sun H, Zhang J, et al. The effect of creatine and coenzyme q10 combination therapy on mild cognitive impairment in Parkinson's disease. Eur Neurol. 2015;73:205-211.
26. Kieburtz K, Tilley BC, Elm JJ, Babcock D, Hauser R, Ross GW, et al. Effect of creatine monohydrate on clinical progression in patients with Parkinson disease: a randomized clinical trial. JAMA. 2015;313:584-93.
27. Bender A, Koch W, Elstner M, Schombacher Y, Bender J, Moeschl M, et al. Creatine supplementation in Parkinson disease: a placebo-controlled randomized pilot trial. Neurology. 2006;67:1262-4.
28. Nathan J, Panjwani S, Mohan V, Joshi V, Thakurdesai PA. Efficacy and safety of standardized extract of Trigonella foenum-graecum L seeds as an adjuvant to L-Dopa in the management of patients with Parkinson's disease. Phytother Res. 2014;28:172-8.
29. Yoritaka A, Kawajiri S, Yamamoto Y, Nakahara T, Ando M, Hashimoto K, et al. Randomized, double-blind, placebo-controlled pilot trial of reduced coenzyme Q10 for Parkinson's disease. Parkinsonism Relat Disord. 2015;21:911-6.
30. Devos D, Moreau C, Devedjian JC, Kluza J, Petrault M, Laloux C, et al. Targeting chelatable iron as a therapeutic modality in Parkinson's disease. Antioxid Redox Signal. 2014;21:195-210.
31. Fahn S, Oakes D, Shoulson I, Kieburtz K, Rudolph A, Lang A, et al. Levodopa and the progression of Parkinson's disease. N Eng J Med. 2004;351:2498-508.
32. Schapira AH, McDermott MP, Barone P, Comella CL, Albrecht S, Hsu HH, et al. Pramipexole in patients with early Parkinson's disease (PROUD): a randomised delayed-start trial. Lancet Neurol. 2013;12:747-55.
33. Schneider JS, Gollomp SM, Sendek S, Colcher A, Cambi F, Du W. A randomized, controlled, delayed start trial of GM1 ganglioside in treated Parkinson's disease patients. J Neurol Sci. 2013;324:140-8.
34. Schneider JS, Cambi F, Gollomp SM, Kuwabara H, Brasic JR, Leiby B, et al. GM1 ganglioside in Parkinson's disease: Pilot study of effects on dopamine transporter binding. J Neurol Sci. 2015;356:118-23.
35. Snow BJ, Rolfe FL, Lockhart MM, Frampton CM, O'Sullivan JD, Fung V, et al. A double-blind, placebo-controlled study to assess the mitochondria-targeted antioxidant MitoQ as a disease-modifying therapy in Parkinson's disease. Mov Disord. 2010;25:1670-4.
36. Rascol O, Olanow W, Brooks D, Koch P, Truffinet R, Bejuit R. A 2-year, multicenter, placebo-controlled, double-blind, parallel-group study of the effect of riluzole on Parkinson's disease progression. Mov Disord. 2002;17:S39.
37. Jankovic J, Hunter C. A double-blind, placebo-controlled and longitudinal study of riluzole in early Parkinson's disease. Parkinsonism Relat Disord. 2002;8:271-6.
38. Guilford Pharmaceuticals Inc: Final phase II GPI 1485 (NIL-A) imaging data presented at the annual meeting of the American Academy of Neurology. PR Newswire. 2002. http://www.prnewswire.co.uk/news-releases/final-phase-ii-gpi-1485-nil-a-imaging-data-presented-at-the-annual-meeting-of-the-american-academy-of-neurology-155593265.html. Accessed 22 Sep 2015.
39. Schwab R, England A. Projection technique for evaluating surgery in Parkinson's disease. In Third Symposium on Parkinson's Disease. Edited by Gillingham F, Donaldson I. Edinburgh: E&S Livingstone; 1969.
40. Jenkinson C, Fitzpatrick R, Peto V, Greenhall R, Hyman N. The Parkinson's Disease Questionnaire (PDQ-39): development and validation of a Parkinson's disease summary index score. Age Ageing. 1997;26:353-7.
41. Smith A. Symbol digit modalities test: Manual. Los Angeles: Western Psychological Services; 1982.
42. van Swieten JC, Koudstaal PJ, Visser MC, Schouten HJ, van GJ. Interobserver agreement for the assessment of handicap in stroke patients. Stroke. 1988;19:604-7.
43. Strauss E, Sherman E, Spreen O. Rey Auditory Verbal Learning Test. In Compendium of Neuropsychological Tests. 3rd edition: Oxford University Press; 2006. p776-807.
44. Nasreddine ZS, Phillips NA, Bedirian V, Charbonneau S, Whitehead V, Collin I, et al. The Montreal Cognitive Assessment, MoCA: a brief screening tool for mild cognitive impairment. J Am Geriatr Soc. 2005;53:695-9.
45. Fahn S, Eton RL, UPDRS Development Committee. The Unified Parkinson's Disease Rating Scale. In Recent Developments in Parkinson's Disease. Edited by Fahn S, Marsden CD, Calne D, et al. Florham Park, New Jersey: Macmillan Healthcare Information; 1987. p153-63.
