# Supplementary material for: A review of clinical trial designs used to detect a disease-modifying effect of drug therapy in Alzheimer’s disease and Parkinson’s disease
Source: BMC Neurol. 2016 Jun 16;16:92. doi: 10.1186/s12883-016-0606-3 (PMC4910262; doi:10.1186/s12883-016-0606-3)
Supplement: Additional file 11: — Outcomes of published AD RCTs. (DOCX 62 kb) [file 12883_2016_606_MOESM11_ESM.docx]

**Additional file 11: Outcomes of published randomised controlled trials of putative disease-modifying agents in Alzheimer’s disease**

| **Trial** | **Pre-defined trial length (months)** | **Percentage of participants** | | | **Did investigators feel disease-modification was demonstrated?** | **Did trial conclusively prove disease-modification?** | **Comments** |
| --- | --- | --- | --- | --- | --- | --- | --- |
|  |  | **Completed follow-up** | **Reached trial endpoint** | **Included in primary analyses** |  |  |  |
| Aβ immunisation [1] | 15 | 74%  (n=276) | n/a | Unclear | Possibly | No | In active-treatment arm only analysed outcome in those subjects that developed a serum antibody response to vaccination.  Trial terminated early due to cases of meningoencephalitis. |
| LEADe [2] | 18 | 71%  (n=452) | n/a | 96%  (n=614) | No | No | No significant difference in the change in primary or secondary outcomes between the treatment groups over 18 months.  As primary endpoints were not met the authors did not report the withdrawal phase results.  Whilst atorvastatin was associated with a significantly smaller annualised decline in hippocampal volume, whole brain atrophy rates were not different between the groups. |
| Bapineuzumab (phase 3) APOE ε4 carriers [3] | 18 | 72%  (n=806) | n/a | 97%  (n=1090) | No | No | Despite a significantly smaller increase in amyloid burden and a significant reduction in CSF phospho-tau in the active-agent compared to placebo treated group, no treatment benefit with regards to primary clinical outcomes was observed.  No significant change in brain volume as assessed by MRI. |
| Bapineuzumab (phase 3) APOE ε4 non-carriers [3] | 18 | 73%  (n=978) | n/a | 84%  (n=1114) | No | No | No significant benefit on primary clinical outcome measures in the bapineuzumab-treated groups compared to placebo.  No treatment difference in PiB PET or CSF phospho-tau for pooled bapineuzumab doses.  No treatment difference in brain volume as assessed by MRI |
| Bapineuzumab (phase 2) [4] | 18 | 68%  (n=158) | n/a | 98%  (n=229) | No | No | No significant differences demonstrated between groups in the primary efficacy analysis.  No significant difference in the change of any CSF biomarker or MRI volume measurement between the groups.  Study underpowered. Initially designed to assess safety but protocol later modified to assess efficacy. |
| ABBY [5, 6] | 17 | Unclear | n/a | Unclear | Possibly | No | Non-significant reduction in the decline in primary outcome measures throughout the trial in those treated with the higher dosage of active-agent compared to placebo.  No effect seen in those treated with the lower dosage. |
| **Trial** | **Pre-defined trial length (months)** | **Percentage of participants** | | | **Did investigators feel disease-modification was demonstrated?** | **Did trial conclusively prove disease-modification?** | **Comments** |
|  |  | **Completed follow-up** | **Reached trial endpoint** | **Included in primary analyses** |  |  |  |
| BLAZE [6-8] | 17 | Unclear | n/a | Unclear | Possibly | No | Non-significant reduction in cognitive and global functional decline in those treated with the higher dosage of active-agent compared to placebo.  PET data suggested reduction of amyloid accumulation in those treated with the active-agent. |
| Scyllo-inositol [9] | 18 | 39%  (n=139) | n/a | 47%  (n=166) | No | No | Higher incidence of significant adverse events in those treated with the two highest doses of scyllo-inositol. Therefore, those arms were terminated early and subsequent comparisons made between the placebo group and those treated with the lowest dose.  No significant difference in primary clinical outcomes or MRI ventricular volume measurements between these groups.  CSF Aβ42 decreased significantly more in those treated with active-agent than placebo.  Study underpowered following early termination of two arms. |
| IDENTITY [10, 11] | 18¥ | 30%  (n=463) | n/a | 99%  (n=1534) | No | No | Preliminary results from IDENTITY and IDENTITY-2 showed that semagacestat was associated with worsening of clinical measures of cognition and ability to perform activities of daily living.  Given this, the study drug was stopped and the trials simply continued to collect safety data. |
| IDENTITY2 [12] | 18 | 5%  (n=56) | n/a | Unclear | No | No |  |
| Simvastatin [13] | 6 | 84%  (n=37) | n/a | 84%  (n=37) | No | No | Simvastatin did not significantly alter CSF levels of Aβ.  A beneficial effect of treatment on the change in the MMSE was observed, but not on the ADAS-cog. |
| EXPEDITION 1 [14] | 18 | 73%  (n=740) | n/a | Unclear | No | No | Primary endpoints were not met in either EXPEDITION 1 or EXPEDITION 2. Furthermore, in a pre-specified secondary analysis pooling data from subjects with mild Alzheimer’s disease (AD) in EXPEDITION 1 and EXPEDITION 2, no statistically significant effect of the active agent on the primary endpoints was observed. |
| EXPEDITION 2 [14] | 18 | 78%  (n=806) | n/a | Unclear | No | No |  |
| Tarenflurbil (phase 3) [15] | 18 | 62%  (n=1046) | n/a | 98%  (n=1649) | No | No | No significant difference between the groups in the change in primary outcomes over the trial period, or in the slopes for either of the primary outcomes.  Similarly, no analysis of secondary outcomes or post hoc analyses favoured tarenflurbil. |

| **Trial** | | **Pre-defined trial length (months)** | | **Percentage of participants** | | | | | **Did investigators feel disease-modification was demonstrated?** | **Did trial conclusively prove disease-modification?** | **Comments** |  |
| --- | --- | --- | --- | --- | --- | --- | --- | --- | --- | --- | --- | --- |
|  |  |  |  | **Completed follow-up** | | **Reached trial endpoint** | | **Included in primary analyses** |  |  |  |  |
| Tarenflurbil (phase 2) [16] | | 24 | | 33%  (n=70) | | n/a | | 90%  (n=189) | Yes – in mild AD | No | Efficacy analyses were conducted separately on mild and moderate subgroups, defined by the MMSE, as the primary analytical model found an interaction between baseline scores and treatment group for all primary outcomes at 24 months.  Those with mild disease in the high-dose tarenflurbil early-start group displayed lower rates of decline in all primary outcomes over 24 months than those in the high-dose tarenflurbil delayed-start group, and those in the combined high and low-dose tarenflurbil delayed-start group.  No consistent effects were seen in patients with moderate AD.  Only a small numbers of participants completed the extended phase, but despite this analyses were then conducted on subgroups of patients. |  |
| Alphase [17] | | 18 | | 75%  (n=790) | | n/a | | 96%  (n=1005) | No | No | Planned analyses did not show significant differences in primary or secondary outcomes between treatment groups.  Planned models revealed a highly significant site effect.  Post-hoc analyses showed a non-significant trend towards a treatment effect with ADAS-cog, and significantly less loss of hippocampal volume in those treated with either dose of tramiprosate compared to placebo.  Authors conceded that they failed to adjust for confounding factors in their planned analyses. |  |
| DARAD [18] | | 12 | | 90%  (n=365) | | n/a | | 100%  (n=406) | No | No | Trial terminated early: interim analysis showed neither rifampicin, doxycycline or combination therapy were better than placebo.  Indeed, both rifampicin and doxycycline resulted in a greater deterioration in terms of the SADAS-cog than the placebo group. |  |
| T-817MA [19] | | 12 | | 67%  (n=249) | | n/a | | Unclear | Showed ‘potential’ | No | No significant difference in the primary outcome or volumetric MRI measurements between groups at 12 months.  However, the authors felt that a statistically non-significant reduction in the change in ADAS-cog between groups over 12 months suggested potential of T-817MA to be disease-modifying. |  |
| Celecoxib [20] | | 12 | | 77%  (n=328) | | n/a | | 97%  (n=414) | No | No | No significant difference in primary outcomes at 12 months between the treatment groups. |  |
| **Trial** | | **Pre-defined trial length (months)** | | **Percentage of participants** | | | | | | **Did investigators feel disease-modification was demonstrated?** | **Did trial conclusively prove disease-modification?** | **Comments** |
|  |  |  |  | **Completed follow-up** | | **Reached trial endpoint** | | **Included in primary analyses** | |  |  |  |
| DAD2000 [21] | | 12 | | Unclear | | n/a | | Unclear | | No | No | No significant difference over 12 months in terms of the change in the ADAS-cog between treatment arms. |
| Diclofenac + misoprostol [22] | | 6 | | 63%  (n=26) | | n/a | | 76%  (n=31) | | No | No | No significant differences between those treated with the active-agent or placebo for any outcome measure. |
| Docosahexaenoic acid [23] | | 18 | | 73%  (n=295) | | n/a | | 100%  (n=402) | | No | No | No beneficial treatment effect seen on either primary outcome.  The rate of brain atrophy was not affected by treatment. |
| OmegAD [24] | | 12 | | 85%  (n=174) | | n/a | | 85%  (n=174) | | Possibly in patients with very mild AD  (MMSE > 27) | No | No significant difference in the decline of cognitive function between the groups at 6 or 12 months.  Post-hoc analyses found that in a subgroup with very mild cognitive impairment (MMSE > 27) there was a significant reduction in the rate of decline in the MMSE in the early-start group compared to the delayed-start group.  A similar arrest in decline was observed in the equivalent delayed-start subgroup between 6 and 12 months. |
| Escitalopram [25] | | 12 | | 77%  (n=57) | | n/a | | Unclear | | No | No | No significant difference in the change in brain volumes or secondary clinical outcomes over 12 months between groups. |
| Hydroxychloroquine [26] | | 18 | | 82%  (n=138) | | n/a | | 100%  (n=168) | | No | No | No treatment benefit observed on the change in either the primary or secondary outcome measures. |
| Ibuprofen [27] | | 12 | | 73%  (n=97) | | n/a | | 100%  (n=132) | | No | No | No significant difference over 12 months in any primary or secondary outcome measure between treatment arms. |
| Dutch  Indomethacin [28] | | 12 | | 75%  (n=38) | | n/a | | 84%  (n=43) | | No | No | No significant difference in primary or secondary outcomes between the treatment groups after one year.  Trial recruitment was discontinued after four years due to difficulties recruiting and, therefore, only around a third of the anticipated numbers of participants were recruited. |
| American indomethacin [29] | | 6 | | 64% (n=28) | | n/a | | 64%  (n=28) | | Probably | No | After six months there was a significant treatment benefit seen with regards to the change in the MMSE and ADAS, but not on the BNT or Token Test. |
| Masitinib (phase 2) [30] | | 6 | | 44%  (n=15) | | n/a | | 65%  (n=22) | | Possibly | No | Significant slowing of decline in the primary outcome in those treated with the active-agent versus placebo.  Low numbers of subjects completed follow-up due to the death of an investigator. |

| **Trial** | **Pre-defined trial length (months)** | **Percentage of participants** | | | **Did investigators feel disease-modification was demonstrated?** | **Did trial conclusively prove disease-modification?** | **Comments** |
| --- | --- | --- | --- | --- | --- | --- | --- |
|  |  | **Completed follow-up** | **Reached trial endpoint** | **Included in primary analyses** |  |  |  |
| Prednisone [31] | 12 | 67% (n=92) | n/a | 92%  (n=127) | No | No | No significant treatment benefit observed on the change in the ADAS-cog over one year.  Significantly greater behavioural decline, as measured by the BPRS, in those treated with prednisone than placebo. |
| Resveratrol [32] | 12 | 87%  (n=104) | n/a | Unclear | No | No | Significant decline in CSF and plasma Aβ40 levels in placebo group compared to active-agent arm.  Brain volume declined significantly more in the active-agent group than in the placebo group.  No effect on other CSF, plasma or imaging biomarkers, or on clinical outcome measures. |
| Rofecoxib [33] | 12 | 70%  (n=481) | n/a | 94%  (n=648) | No | No | No significant difference between groups in any clinical measure after 12 months.  No evaluation of data during the wash-out period was made as given the lack of a treatment effect at 12 months the randomised withdrawal period was felt to be irrelevant. |
| Rofecoxib or naproxen [34] | 12 | 76%  (n=267) | 69% (n=242) reached at least 1 endpoint | 100%  (n=351) | No | No | The change in the ADAS-cog over 12 months was not significantly different in the placebo group from that in those treated with naproxen or those treated with rofecoxib.  Results of secondary analyses showed no consistent benefit of either treatment. |
| DAV.I.D.E. [35] | 24 | 51%  (n=109) | n/a | 51%  (n=109) | Possibly | No | Significant improvement over the first year of follow-up in the early-start group compared to the delayed-start group, and they remained relatively unchanged during the second year.  After starting active treatment the delayed-start group showed improvement on the Gottfries-Bråne-Steen Scale, but they still lagged behind the early-start group at the end of the study.  No statistical analyses comparing outcomes in the early-start and delayed-start groups over the whole study period are presented. |

| **Trial** | **Pre-defined trial length (months)** | **Percentage of participants** | | | **Did investigators feel disease-modification was demonstrated?** | **Did trial conclusively prove disease-modification?** | **Comments** |
| --- | --- | --- | --- | --- | --- | --- | --- |
|  |  | **Completed follow-up** | **Reached trial endpoint** | **Included in primary analyses** |  |  |  |
| Nutritional formulation [36] | 12 | 23%  (n=33) | n/a | 74%  (n=106) | Yes | No | Subjects receiving the active-agent early improved significantly compared to the placebo group in terms of the change in primary outcome measures over 3 months.  Those initially randomised to the active agent showed continued improvement over 12 months, or maintained previous improvement.  Trial closed early after a company expressed interest in marketing the active agent to ‘maximise availability to the public’. |
| Czech/Slovak selegiline [37] | 6 | 82%  (n=142) | n/a | 83%  (n=143) | Yes | No | Subjects were divided into two groups depending on whether they had a normal or pathological CLOX.  There was a significant improvement in Sternberg’s Memory Scanning Test in those with a pathological CLOX treated with selegiline, as compared to those on placebo.  In those with a pathological CLOX there was a significantly smaller decrease in the dominant EEG frequency in those treated with selegiline than those treated with placebo. No such difference was found in the subgroup with a normal CLOX.  The authors noted a 6-12 week delay before improvement was seen in Sternberg’s memory scanning test in the selegiline group compared to the placebo group. They interpreted this as indicating neuroprotection or neurorescue. |
| Canadian selegiline [38] | 6 | 72%  (n=43) | n/a | 72%  (n=43) | No | No | No significant difference in the change in primary or secondary outcomes between the treatment groups.  No results reported for the wash-out analysis. |
| KUOSTAD [39] | 36 | 68%  (n=54) | n/a | Unclear | Possibly | No | The MMSE score deteriorated significantly more rapidly in the placebo than selegiline group over 18 months.  Despite the abstract stating that the pre-specified three-year follow-up period had been completed only results for a maximum of 18 months follow-up are detailed. |
| Nebraska selegiline [40] | 15 | 85%  (n=33) | n/a | 74%  (n=29) | No | No | No significant difference between the groups in terms of the change in any primary outcome measure.  The authors state in the discussion that the wish they had conducted additional analyses after a washout period. |

| **Trial** | **Pre-defined trial length (months)** | **Percentage of participants** | | | **Did investigators feel disease-modification was demonstrated?** | **Did trial conclusively prove disease-modification?** | **Comments** |
| --- | --- | --- | --- | --- | --- | --- | --- |
|  |  | **Completed follow-up** | **Reached trial endpoint** | **Included in primary analyses** |  |  |  |
| Selegiline & tocopherol [41] | 24 | 35%  (n=121) | 58%  (n=197) | 100%  (n=341) | Possibly | No | Unadjusted primary outcome analyses showed no significant differences between those treated with placebo and those treated with selegiline, α-tocopherol or combination treatment.  Analyses adjusted for baseline MMSE showed that selegiline, α-tocopherol and combination treatment all significantly delayed the time until the primary outcome was reached. |
| VALID [42] | 24 | 39%  (n=122) | 17%  (n=54) | 100%  (n=313) | No | No | Time to the primary end point did not differ between the treatment arms, and neither did the change in any of the secondary clinical outcome measures.  The group treated with divalproex sodium showed greater loss in hippocampal and whole-brain volume and greater ventricular expansion than the placebo group over 12 months.  Results of wash-out analysis were not fully reported. |
| TauRx (phase 2) [43] | 6 | 74%  (n=238) | n/a | Unclear | Yes | No | No statistically significant difference in primary outcome measures over 24 weeks between active-agent and placebo groups with regards to either the lower or higher strength dosages.  Significant benefit of moderate dosage in terms of reducing clinical decline on primary outcome measures. |
| Donepezil MRI/MRS [44] | 6 | 76%  (n=51) | n/a | 93%  (n=62) | Possibly | No | Whilst there were significant differences between groups at earlier time points, the between-group differences in N-acetylaspartate at the end of the study, and after the 6 week wash-out period, failed to reach statistical significance.  During the study the ADAS-cog improved in the donepezil group concurrently with an increase in brain N-acetylaspartate concentration, but deteriorated in the placebo group.  Significantly smaller decreases in hippocampal volume were observed in those treated with donepezil than placebo. |
| Donepezil international [45] | 6 | 77%  (n=631) | n/a | Unclear | No | No | A significant beneficial treatment effect of either dose of donepezil on primary and secondary outcomes compared to those treated with placebo was demonstrated.  After the wash-out period efficacy scores in the donepezil groups reverted to levels similar to those in the placebo group. |

| **Trial** | **Pre-defined trial length (months)** | **Percentage of participants** | | | **Did investigators feel disease-modification was demonstrated?** | **Did trial conclusively prove disease-modification?** | **Comments** |
| --- | --- | --- | --- | --- | --- | --- | --- |
|  |  | **Completed follow-up** | **Reached trial endpoint** | **Included in primary analyses** |  |  |  |
| Donepezil USA clinical [46] | 6 | 78%  (n=368) | n/a | 96%  (n=455) | No | No | Significant treatment benefit seen with donepezil over placebo on the change in the ADAS-cog and CIBIC+ over 6 months.  However, after the wash-out period no significant differences in these measures remained between the treatment groups. |
| Galantamine [47] | 12 | 42%  (n=268) | n/a | Unclear | Possibly | No | Those treated early with galantamine had a better outcome on the ADAS-cog at 12 months than those who received placebo for the first 6 months.  Improvements in the ADAS-cog appeared within one week of reaching a galantamine dose of 24mg and increased after three months in both galantamine groups, likely indicated a symptomatic treatment response. |
| GAP Study [48, 49] | 18 | 77%  (n=302) | n/a | 77%  (n=301) | No | No | No significant difference between either active-agent treatment groups and the placebo group in terms of the change in ADAS-cog or ADCS-ADL over 18 months. |
| CONCERT [50, 51] | 12 | Unclear | n/a | Unclear | No | No | No significant improvement in either primary outcome measure in those treated with active-agent compared to placebo over one year. |
| CONNECTION [52, 53] | 6 | Unclear | n/a | Unclear | No | No | No significant improvement in primary outcome measures in those treated with active-agent compared to placebo over six months. |
| Russian Dimebon [54] | 6 | 85%  (n=155) | n/a | 100%  (n=183) | Possibly | No | The change over 6 months in all five outcome measures significantly favoured the active-agent. In an extension to 12 months (n=134) the change in all five outcome measures still significantly favoured the active-agent, and there was evidence of increasing benefit (divergence from placebo) of the active-agent. |
| Cerebrolysin [55] | 6 | 85%  (n=237) | n/a | 90%  (n=251) | Possibly | No | Reverse u-shaped dose-response, with significant treatment benefit in those treated with 10ml of cerebrolysin.  All dosage groups of cerebrolysin showed a marked symptomatic benefit at 4 weeks, whereas no benefit was seen in the placebo group.  The beneficial effect observed in the 10ml cerebrolysin group was largely maintained three months after cessation of active treatment, interpreted by the authors as indicating possible neuroprotection. |

| **Trial** | **Pre-defined trial length (months)** | **Percentage of participants** | | | **Did investigators feel disease-modification was demonstrated?** | **Did trial conclusively prove disease-modification?** | **Comments** |
| --- | --- | --- | --- | --- | --- | --- | --- |
|  |  | **Completed follow-up** | **Reached trial endpoint** | **Included in primary analyses** |  |  |  |
| Memantine PET [56] | 6 | 85%  (n=22) | n/a | 85%  (n=22) | Possibly | No | The group treated with memantine declined significantly less on the SIB and, as measured by FDG PET, in brain regions known to be affected by AD than the placebo-treated group.  No significant difference between groups in CSF biomarkers. |
| Memantine MRI [57] | 12 | 78%  (n=217) | n/a | 82%  (n=228) | No | No | No significant difference in the whole brain or hippocampal atrophy rate between the treatment groups. |
| Memantine MRS [58] | 12 | 76%  (n=13) | n/a | 59%  (n=10) | No | No | No significant treatment benefit seen with memantine relative to placebo on the change in the NAA/Cr ratio or ADAS-cog. |
| Memantine vs. donepezil MRS [59] | 6 | 94%  (n=63) | n/a | 94%  (n=63) | No | No | No significant treatment benefit seen with memantine, relative to those treated with donepezil, on either metabolite ratios or clinical rating scales. |
| Memantine multimodal [60] | 12 | 65%  (n=24) | n/a | Unclear | Yes | No | Slower decline, but statistically non-significant, in glucose metabolism in all brain areas and hippocampal volume in memantine than placebo treated subjects.  Pilot study. |
| REFLECT-1 [61] | 6 | 77%  (n=448) | n/a | 95%  (n=553) | No | No | No significant benefit of either dosage of active-agent on primary outcome measures observed during the trial period. |
| Rosiglitazone genetics [62] | 6 | 87%  (n=449) | n/a | 99%  (n=511) | Yes | No | No statistically significant differences on primary endpoints were detected between the placebo arm and any dose of rosiglitazone.  However, a significant improvement in ADAS-cog was observed in those subjects on the highest dose of rosiglitazone who were also negative for the APOE ε4 allele. |
| Azeliragon [63] | 18 | 40%  (n=160) | n/a | 95%  (n=380) | No | No | Interim safety analysis at 6 months led to the higher dose arm being discontinued, and a futility analysis at 12 months led to all subjects discontinuing the study drug. Follow-up was, however, continued.  Significantly smaller decline in ADAS-cog over 18 months in the low-dose active-agent group. |

**Key**

‘Pre-defined trial length’ refers to the length of the trial not including any washout period unless the pre-defined primary analyses related to the change in an outcome measure from baseline until the end of the washout period (these studies are marked with an asterisk (*). Similarly, the column relating to the percentage of patients that reached the trial endpoint relates to the number doing so within the pre-defined trial duration, excluding the washout period, unless the pre-defined primary analyses related to the change in characteristics from baseline until the end of the washout period. In some delayed-start trials (marked with ¥) the primary analyses related to the change in an outcome measure from baseline until the start of the delayed-start phase of the study (i.e. before those taking placebo commenced taking the active-agent).

The percentage of participants who ‘completed follow-up’ corresponds to the number of patients who continued the study intervention until the end of the double-blind treatment period. Patients who reached a pre-defined endpoint (e.g. commencement of symptomatic treatment or death) which led to their withdrawal from the trial before completing the pre-defined treatment period are not included in this figure.

**Clinical rating scales**

ADAS Alzheimer’s Disease Assessment Scale [64]

ADAS-cog Alzheimer’s Disease Assessment Scale – cognitive subscale [64] [No distinction made between different versions]

ADCS-ADL Alzheimer’s Disease Cooperative Study – Activities of Daily Living inventory [65]

BNT Boston Naming Test [66]

BPRS Brief Psychiatric Rating Scale [67]

CIBIC+ Clinician Interview-Based Impression of Change with Caregiver Input (ADCS version) [68]

CLOX Clock Drawing Test [69]

Gottfries-Bråne-Steen Scale Gottfries-Bråne-Steen Scale [70]

MMSE Mini-Mental State Examination [71]

SADAS-cog Standardised Alzheimer’s Disease Assessment Scale [72]

SIB Severe Impairment Battery [73]

Token Test Token Test [74] **Proteins and metabolites**

Aβ Amyloid beta

**Biomarker modalities** Aβ42 Amyloid beta isomer, length 42 amino acids

CSF Cerebrospinal Fluid APOE Apolipoprotein E

EEG Electroencephalography NAA/Cr ratio N-acetylaspartate/Creatine ratio

MRI Magnetic Resonance Imaging

MRS Magnetic Resonance Spectroscopy **PET ligands**

PET Positron Emission Tomography PiB [^11^C]Pittsburgh compound B

FDG [^18^F]-2-fluoro-2-deoxyglucose

**References**

1. Gilman S, Koller M, Black RS, Jenkins L, Griffith SG, et al. Clinical effects of Abeta immunization (AN1792) in patients with AD in an interrupted trial. Neurology. 2005;64:1553-62.
2. Feldman HH, Doody RS, Kivipelto M, Sparks DL, Waters DD, Jones RW, et al. Randomized controlled trial of atorvastatin in mild to moderate Alzheimer disease: LEADe. Neurology. 2010;74:956-64.
3. Salloway S, Sperling R, Fox NC, Blennow K, Klunk W, Raskind M, et al. Two phase 3 trials of bapineuzumab in mild-to-moderate Alzheimer's disease. N Engl J Med. 2014;370:322-33.
4. Salloway S, Sperling R, Gilman S, Fox NC, Blennow K, Raskind M, et al. A phase 2 multiple ascending dose trial of bapineuzumab in mild to moderate Alzheimer disease. Neurology. 2009;73:2061-70.
5. A Study to Evaluate the Efficacy and Safety of MABT5102A in Patient With Mild to Moderate Alzheimer's Disease (ABBY). ClinicalTrials.gov. 2015. http://www.clinicaltrials.gov/ct2/show/NCT01343966. Accessed 9 Oct 2015.
6. Roche announces phase II clinical results of crenezumab in Alzheimers disease. Roche. 2014. http://www.roche.com/investors/updates/inv-update-2014-07-16.htm. Accessed 12 Oct 2015.
7. A Study to Evaluate the Impact of MABT5102A on Brain Amyloid Load and Related Biomarkers in Patients with Mild to Moderate Alzheimer's Disease. ClinicalTrials.gov. 2015. http://www.clinicaltrials.gov/ct2/show/NCT01397578. Accessed 9 Oct 2015.
8. AC Immune receives milestone payment for crenezumab moving into phase III clinical development in Alzheimer's disease. Swiss Biotech. 2015. http://www.swissbiotech.org/b/index.php?1=1&id=665679. Accessed 12 Oct 2015.
9. Salloway S, Sperling R, Keren R, Porsteinsson AP, van Dyck CH, Tariot PN, et al. A phase 2 randomized trial of ELND005, scyllo-inositol, in mild to moderate Alzheimer disease. Neurology. 2011;77:1253-62.
10. Effect of LY450139 on the Long Term Progression of Alzheimer's Disease. ClinicalTrials.gov. 2015. http://www.clinicaltrials.gov/ct2/show/NCT00594568. Accessed 9 Oct 2015.
11. Doody RS, Raman R, Farlow M, Iwatsubo T, Vellas B, Joffe S, et al. A phase 3 trial of semagacestat for treatment of Alzheimer's disease. N Engl J Med. 2013;369:341-50.
12. Effect of LY450139, on the progression of Alzheimer's disease as compared with placebo (IDENTITY-2). ClinicalTrials.gov. 2015. http://www.clinicaltrials.gov/ct2/show/NCT00762411. Accessed 22 Sep 2015.
13. Simons M, Schwarzler F, Lutjohann D, von BK, Beyreuther K, Dichgans J, et al. Treatment with simvastatin in normocholesterolemic patients with Alzheimer's disease: A 26-week randomized, placebo-controlled, double-blind trial. Ann Neurol. 2002;52:346-50.
14. Doody RS, Thomas RG, Farlow M, Iwatsubo T, Vellas B, Joffe S, et al. Phase 3 trials of solanezumab for mild-to-moderate Alzheimer's disease. N Engl J Med. 2014;370:311-21.
15. Green RC, Schneider LS, Amato DA, Beelen AP, Wilcock G, Swabb EA, et al. Effect of tarenflurbil on cognitive decline and activities of daily living in patients with mild Alzheimer disease: a randomized controlled trial. JAMA. 2009;302:2557-64.
16. Wilcock GK, Black SE, Hendrix SB, Zavitz KH, Swabb EA, Laughlin MA. Efficacy and safety of tarenflurbil in mild to moderate Alzheimer's disease: a randomised phase II trial. Lancet Neurol. 2008;7:483-93.
17. Aisen PS, Gauthier S, Ferris SH, Saumier D, Haine D, Garceau D, et al.Tramiprosate in mild-to-moderate Alzheimer's disease - a randomized, double-blind, placebo-controlled, multi-centre study (the Alphase Study). Arch Med Sci. 2011;7:102-11.
18. Molloy DW, Standish TI, Zhou Q, Guyatt G. A multicenter, blinded, randomized, factorial controlled trial of doxycycline and rifampin for treatment of Alzheimer's disease: the DARAD trial. Int J Geriatr Psychiatry. 2013;28:463-70.
19. Schneider L, Porsteinsson A, Farlow M, Shimakura A, Nakagawa M, Iwakami N. The neuroprotective and neurotrophic agent T-817MA for Alzheimer's disease: Randomized, double-blind, placebo-controlled proof-of-concept trial outcomes. Alzheimers Dement. 2013;9:530-1.
20. Soininen H, West C, Robbins J, Niculescu L: Long-term efficacy and safety of celecoxib in Alzheimer's disease. Dement Geriatr Cogn Disord. 2007;23:8-21.
21. Alzheimer disease: phase 2 trial results reported by Immune Network Ltd. The Free Library. 2002. http://www.thefreelibrary.com/Alzheimer%20Disease:%20Phase%202%20Trial%20Results%20Reported%20by%20Immune%20Network...-a092852880. Accessed 22 Sep 2015.
22. Scharf S, Mander A, Ugoni A, Vajda F, Christophidis N. A double-blind, placebo-controlled trial of diclofenac/misoprostol in Alzheimer's disease. Neurology. 1999;53:197-1.
23. Quinn JF, Raman R, Thomas RG, Yurko-Mauro K, Nelson EB, van DC, et al. Docosahexaenoic acid supplementation and cognitive decline in Alzheimer disease: a randomized trial. JAMA. 2010;304:1903-11.
24. Freund-Levi Y, Eriksdotter-Jonhagen M, Cederholm T, Basun H, Faxen-Irving G, Garlind A, et al. Omega-3 fatty acid treatment in 174 patients with mild to moderate Alzheimer disease: OmegAD study: a randomized double-blind trial. Arch Neurol. 2006;63:1402-8.
25. Lee DY, Kim KW, Jhoo JH, Ryu S, Choo IH, Seo EH, at al. A multicenter, randomized, placebo-controlled, double-blind clincial trial of escitalopram on its atrophy-delaying effect in Alzheimer's disease. Alzheimers Dement. 2012;8:603.
26. Van Gool WA, Weinstein HC, Scheltens P, Walstra GJ. Effect of hydroxychloroquine on progression of dementia in early Alzheimer's disease: an 18-month randomised, double-blind, placebo-controlled study. Lancet. 2001;358:455-460.
27. Pasqualetti P, Bonomini C, Dal FG, Paulon L, Sinforiani E, Marra C, et al. A randomized controlled study on effects of ibuprofen on cognitive progression of Alzheimer's disease. Aging Clin Exp Res. 2009;21:102-10.
28. de JD, Jansen R, Hoefnagels W, Jellesma-Eggenkamp M, Verbeek M, Borm G, et al. No effect of one-year treatment with indomethacin on Alzheimer's disease progression: a randomized controlled trial. PLoS One. 2008;3:e1475.
29. Rogers J, Kirby LC, Hempelman SR, Berry DL, McGeer PL, Kaszniak AW, et al. Clinical trial of indomethacin in Alzheimer's disease. Neurology. 1993;43:1609-1611.
30. Piette F, Belmin J, Vincent H, Schmidt N, Pariel S, Verny M, et al. Masitinib as an adjunct therapy for mild-to-moderate Alzheimer's disease: a randomised, placebo-controlled phase 2 trial. Alzheimers Res Ther. 2011;3:16.
31. Aisen PS, Davis KL, Berg JD, Schafer K, Campbell K, Thomas RG, et al. A randomized controlled trial of prednisone in Alzheimer's disease. Alzheimer's Disease Cooperative Study. Neurology. 2000;54:588-593.
32. Turner RS, Thomas RG, Craft S, van Dyck CH, Mintzer J, Reynolds BA, et al. A randomized, double-blind, placebo-controlled trial of resveratrol for Alzheimer disease. Neurology. 2015. doi:10.1212/WNL.0000000000002035.
33. Reines SA, Block GA, Morris JC, Liu G, Nessly ML, Lines CR, et al. Rofecoxib: no effect on Alzheimer's disease in a 1-year, randomized, blinded, controlled study. Neurology. 2004;62:66-71.
34. Aisen PS, Schafer KA, Grundman M, Pfeiffer E, Sano M, Davis KL, et al. Effects of rofecoxib or naproxen vs placebo on Alzheimer disease progression: a randomized controlled trial. JAMA. 2003;289:2819-2826.
35. Cucinotta D, De Leo D, Frattola L, Trabucchi M, Albizatti M, Beltramelli A, et al. Dihydroergokryptine as long-term treatment of Alzheimer type dementia: a multicenter two-year follow-up. Arch Gerontol Geriatr. 1998;Suppl 6:103-10.
36. Remington R, Bechtel C, Larsen D, Samar A, Doshanjh L, Fishman P, et al. A Phase II Randomized Clinical Trial of a Nutritional Formulation for Cognition and Mood in Alzheimer's Disease. J Alzheimers Dis. 2015;45:395-405.
37. Filip V, Kolibas E. Selegiline in the treatment of Alzheimer's disease: a long-term randomized placebo-controlled trial. Czech and Slovak Senile Dementia of Alzheimer Type Study Group. J Psychiatry Neurosci. 1999;24:234-43.
38. Freedman M, Rewilak D, Xerri T, Cohen S, Gordon AS, Shandling M, et al. L-deprenyl in Alzheimer's disease: cognitive and behavioral effects. Neurology. 1998;50:660-668.
39. Koivisto K, Helkala E-L, Hanninen T, Vanhanen M, Aaltonen H, Reinikainen K, et al. Three-year follow-up of long-term selegiline treatment of Alzheimer's disease. J Neurol. 1995;242:S34-S35.
40. Burke WJ, Roccaforte WH, Wengel SP, Bayer BL, Ranno AE, Willcockson NK. L-deprenyl in the treatment of mild dementia of the Alzheimer type: results of a 15-month trial. J Am Geriatr Soc. 1993;41:1219-25.
41. Sano M, Ernesto C, Thomas RG, Klauber MR, Schafer K, Grundman M, et al. A controlled trial of selegiline, alpha-tocopherol, or both as treatment for Alzheimer's disease. The Alzheimer's Disease Cooperative Study. New Engl J Med. 1997;336:1216-1222.
42. Tariot PN, Schneider LS, Cummings J, Thomas RG, Raman R, Jakimovich LJ, et al. Alzheimer's Disease Cooperative Study Group. Chronic divalproex sodium to attenuate agitation and clinical progression of Alzheimer disease. Arch Gen Psychiatry 2011;68:853-61.
43. Wischik CM, Staff RT, Wischik DJ, Bentham P, Murray AD, Storey JM, et al. Tau aggregation inhibitor therapy: an exploratory phase 2 study in mild or moderate Alzheimer's disease. J Alzheimers Dis. 2015;44:705-20.
44. Krishnan KR, Charles HC, Doraiswamy PM, Mintzer J, Weisler R, Yu X, et al. Randomized, placebo-controlled trial of the effects of donepezil on neuronal markers and hippocampal volumes in Alzheimer's disease. Am J Psychiatry. 2003;160:2003-11.
45. Burns A, Rossor M, Hecker J, Gauthier S, Petit H, Moller HJ, et al. The effects of donepezil in Alzheimer's disease - results from a multinational trial. Dement Geriatr Cogn Disord. 1999;10:237-44.
46. Rogers SL, Farlow MR, Doody RS, Mohs R, Friedhoff LT. A 24-week, double-blind, placebo-controlled trial of donepezil in patients with Alzheimer's disease. Donepezil Study Group. Neurology. 1998;50:136-45.
47. Raskind MA, Peskind ER, Wessel T, Yuan W. Galantamine in AD: A 6-month randomized, placebo-controlled trial with a 6-month extension. The Galantamine USA-1 Study Group. Neurology. 2000;54:2261-68.
48. A Phase 3 Study Evaluating Safety and Effectiveness of Immune Globulin Intravenous (IGIV 10%) for the Treatment of Mild-to-Moderate Alzheimer's Disease. ClinicalTrials.gov. 2015. http://www.clinicaltrials.gov/ct2/show/NCT00818662. Accessed 9 Oct 2015.
49. Relkin N. Results of the GAP 160701 study: A phase 3 clinical trial of intravenous immunoglobulin for mild-to-moderate Alzheimer's disease. Alzheimers Dement. 2013;9:530.
50. Safety and Efficacy Study Evaluating Dimebon in Patients With Mild to Moderate Alzheimer's Disease on Donepezil (CONCERT). ClinicalTrials.gov. 2012. http://www.clinicaltrials.gov/ct2/show/NCT00829374. Accessed 9 Oct 2015.
51. Sweetlove M. Phase III CONCERT Trial of Latrepirdine. Pharm Med. 2012;26:113-5.
52. A Safety and Efficacy Study of Oral Dimebon in Patients With Mild-To-Moderate Alzheimer's Disease (CONNECTION). ClinicalTrials.gov. 2012. http://www.clinicaltrials.gov/ct2/show/NCT00675623. Accessed 9 Oct 2015.
53. Pfizer And Medivation Announce Results From Two Phase 3 Studies In Dimebon (latrepirdine*) Alzheimer's Disease Clinical Development Program. Pfizer. 2010. http://press.pfizer.com/press-release/pfizer-and-medivation-announce-results-two-phase-3-studies-dimebon-latrepirdine-alzhei. Accessed 12 Oct 2015.
54. Doody RS, Gavrilova SI, Sano M, Thomas RG, Aisen PS, Bachurin SO, et al. Effect of dimebon on cognition, activities of daily living, behaviour, and global function in patients with mild-to-moderate Alzheimer's disease: a randomised, double-blind, placebo-controlled study. Lancet. 2008;372:207-15.
55. Alvarez XA, Cacabelos R, Laredo M, Couceiro V, Sampedro C, Varela M, et al. A 24-week, double-blind, placebo-controlled study of three dosages of Cerebrolysin in patients with mild to moderate Alzheimer's disease. Eur J Neurol. 2006;13:43-54.
56. Wang T, Huang Q, Reiman EM, Chen K, Li X, Li G, et al. Effects of memantine on clinical ratings, fluorodeoxyglucose positron emission tomography measurements, and cerebrospinal fluid assays in patients with moderate to severe Alzheimer dementia: a 24-week, randomized, clinical trial. J Clin Psychopharmacol. 2013;33:636-42.
57. Wilkinson D, Fox NC, Barkhof F, Phul R, Lemming O, Scheltens P. Memantine and brain atrophy in Alzheimer's disease: a 1-year randomized controlled trial. J Alzheimers Dis. 2012;29:459-69.
58. Ashford JW, Adamson M, Beale T, La D, Hernandez B, Noda A, et al. MR spectroscopy for assessment of memantine treatment in mild to moderate Alzheimer dementia. J Alzheimers Dis. 2011;26 Suppl 3:331-6.
59. Modrego PJ, Fayed N, Errea JM, Rios C, Pina MA, Sarasa M. Memantine versus donepezil in mild to moderate Alzheimer's disease: A randomized trial with magnetic resonance spectroscopy. Eur J Neurol. 2010;17:405-12.
60. Schmidt R, Ropele S, Pendl B, Ofner P, Enzinger C, Schmidt H, et al. Longitudinal multimodal imaging in mild to moderate Alzheimer disease: a pilot study with memantine. J Neurol Neurosurg Psychiatry. 2008;79:1312-7.
61. Gold M, Alderton C, Zvartau-Hind M, Egginton S, Saunders AM, Irizarry M, et al. Rosiglitazone monotherapy in mild-to-moderate Alzheimer's disease: results from a randomized, double-blind, placebo-controlled phase III study. Dement Geriatr Cogn Disord. 2010;30:131-46.
62. Risner ME, Saunders AM, Altman JF, Ormandy GC, Craft S, Foley IM, et al. Efficacy of rosiglitazone in a genetically defined population with mild-to-moderate Alzheimer's disease. Pharmacogenomics J. 2006;6:246-54.
63. Galasko D, Bell J, Mancuso JY, Kupiec JW, Sabbagh MN, van DC, et al. Clinical trial of an inhibitor of RAGE-Abeta interactions in Alzheimer disease. Neurology. 2014;82:1536-42.
64. Mohs RC, Knopman D, Petersen RC, Ferris SH, Ernesto C, Grundman M, et al. Development of cognitive instruments for use in clinical trials of antidementia drugs: additions to the Alzheimer's Disease Assessment Scale that broaden its scope. The Alzheimer's Disease Cooperative Study. Alzheimer Dis Assoc Disord. 1997;11:S13-S21.
65. Galasko D, Bennett D, Sano M, Ernesto C, Thomas R, Grundman M, et al. An inventory to assess activities of daily living for clinical trials in Alzheimer's disease. The Alzheimer's Disease Cooperative Study. Alzheimer Dis Assoc Disord. 1997;11:S33-S39.
66. Kaplan E, Goodglass H, Weintraub S. The Boston Naming Test. Philadelphia: Lea & Febiger; 1983.
67. Overall JE, Beller SA. The Brief Psychiatric Rating Scale (BPRS) in geropsychiatric research: I. Factor structure on an inpatient unit. J Gerontol. 1984;39:187-193.
68. Knopman DS, Knapp MJ, Gracon SI, Davis CS. The Clinician Interview-Based Impression (CIBI): a clinician's global change rating scale in Alzheimer's disease. Neurology. 1994;44:2315-21.
69. Shulman K, Shedletsky R, Silver I. The challenge of time: clock drawing and cognitive function in the elderly. Int J Geriatr Psychiatry. 1986l;1:135-40.
70. Gottfries CG, Brane G, Gullberg B, Steen G. A new rating scale for dementia syndromes. Arch Gerontol Geriatr. 1982;1:311-30.
71. Folstein MF, Folstein SE, McHugh PR. "Mini-mental state". A practical method for grading the cognitive state of patients for the clinician. J Psychiatr Res. 1975;12:189-98.
72. Standish TI, Molloy DW, Bedard M, Layne EC, Murray EA, Strang D. Improved reliability of the Standardized Alzheimer's Disease Assessment Scale (SADAS) compared with the Alzheimer's Disease Assessment Scale (ADAS). J Am Geriatr Soc. 1996;44:712-6.
73. Saxton J, Swihart AA. Neuropsychological assessment of the severely impaired elderly patient. Clin Geriatr Med. 1989;5:531-543.
74. Brookshire RH. A token test battery for testing auditory comprehension in brain-injured adults. Brain Lang. 1978;6:149-157.
