# Supplementary material for: A review of clinical trial designs used to detect a disease-modifying effect of drug therapy in Alzheimer’s disease and Parkinson’s disease
Source: BMC Neurol. 2016 Jun 16;16:92. doi: 10.1186/s12883-016-0606-3 (PMC4910262; doi:10.1186/s12883-016-0606-3)
Supplement: Additional file 12: — Outcomes of published PD RCTs. (DOCX 52 kb) [file 12883_2016_606_MOESM12_ESM.docx]

**Additional file 12: Outcomes of published randomised controlled trials of putative disease-modifying agents in Parkinson’s disease**

| **Trial** | **Pre-defined trial length (months)** | **Percentage of participants** | | | **Did investigators feel disease-modification was demonstrated?** | **Did trial conclusively prove disease-modification?** | **Comments** |
| --- | --- | --- | --- | --- | --- | --- | --- |
|  |  | **Completed follow-up** | **Reached trial endpoint** | **Included in primary analyses** |  |  |  |
| PRECEPT [1] | ≥ 24 | 25% †  (n=200) | 61%  (n=494) | 100%  (n=806) | No | No | No evidence that CEP-1347 delayed the time until dopaminergic treatment – in some dosage groups it actually shortened it.  Greater deterioration in striatal [^123^I]β-CIT uptake and in the total UPDRS in those receiving CEP-1347 than placebo.  Trial stopped early following a planned interim analysis which revealed that the futility threshold had been reached. |
| Green tea [2, 3] | 12 | Unclear | n/a | Unclear | No | No | No significant difference in the change in the total UPDRS over 12 months between the early-start and delayed-start treatment groups. |
| TCH346 [4] | 12-18 | 59%  (n=177) | 34%  (n=103) | 99%  (n=299) | No | No | TCH346 did not delay the need for dopaminergic treatment.  No symptomatic effect of TCH346 seen during wash-in or wash-out. |
| Exenatide [5] | 14* | 91%  (n=41) | n/a | 98%  (n=44) | No | No | Even after wash-out, exenatide treated subjects experienced a significantly greater improvement in UPDRS (III) than the control group (who deteriorated). However, the authors state that long-duration symptomatic effects may persist beyond 8 weeks and can’t be excluded by this study design. Additionally, not a placebo controlled trial.  No significant change in SPECT measures over 12 months in a subset of 10 subjects on exenatide, but no examination against a control group. |
| H_2_-water [6] | 11 | 94%  (n=17) | n/a | 94%  (n=17) | Larger studies required | No | Significant improvement, relative to those treated with placebo, in the total UPDRS over 11 months in those treated with H_2_-dissolved water.  However, no significant difference between groups in the change in the total UPDRS from baseline until after the wash-out period. |
| ROADS [7] | 12 | 51%  (n=165) | 34%  (n=109) | 100%  (n=321) | No | No | Any dose of lazabemide delayed the need for levodopa.  No significant difference over 12 months in the change in secondary outcome measures between active-agent and placebo groups.  During the wash-out period UPDRS scores declined in the active but not the placebo group (no significant difference between groups).  Fifteen patients in the active arm needed levodopa during washout.  Small, mainly non-significant, symptomatic effects seen on wash-in. |
| Subtherapeutic pergolide [8] | No set trial length | n/a | 78%  (n=83) | 99%  (n=105) | No | No | Although a trend towards delayed levodopa treatment with pergolide was noted the difference from the placebo group was not significant.  Significant wash-in effect and trend for symptomatic benefit on wash-out. |
| **Trial** | **Pre-defined trial length (months)** | **Percentage of participants** | | | **Did investigators feel disease-modification was demonstrated?** | **Did trial conclusively prove disease-modification?** | **Comments** |
|  |  | **Completed follow-up** | **Reached trial endpoint** | **Included in primary analyses** |  |  |  |
| CALM-PD-CIT [9] | 46 | 67%  (n=55) | n/a | 67%  (n=55) | Unable to determine this | No | Striatal [^123^I]β-CIT uptake declined significantly less in those treated with pramipexole than in those treated with levodopa.  Decline in striatal [^123^I]β-CIT uptake correlated with change in total UPDRS in the ‘off’ state over 46 months.  The authors commented that given the comparative design of the study they could not determine if pramipexole was neuroprotective or if levodopa increased the rate of dopaminergic terminal loss. |
| Rasagiline cognition [10] | 6 | Unclear | n/a | Unclear | No | No | No significant change noted in any neuropsychological measures after six months treatment with rasagiline. |
| Russian rasagiline [11] | 6 | 85%  (n=34) | n/a | Unclear | Yes | No | Placebo (vitamin E) treated patients showed a clear increase in motor severity over the course of the trial, while the course of illness was more stable in those treated with rasagiline.  The differences between the groups remained after the wash-out period. |
| ADAGIO [12] | 16 | 81%  (n=954) | n/a | 99% ‡  (n=1164) | Possibly | No | Early treatment with rasagiline 1mg/day showed a beneficial effect on all three primary endpoints.  The change in the total UPDRS over 72 weeks was not significantly different between those treated early with rasagiline 2mg/day and those in whom treatment was delayed.  Lack of consistent results between two dosages – findings with rasagiline 1mg/day could represent a false positive result. |
| TEMPO [13] | 12 | 64%  (n=259) | 30%  (n=121) § | 92%  (n=371) | Possibly | No | The total UPDRS declined less over 12 months in those treated early with rasagiline than in the delayed treatment group.  Possible confounding by using LOCF for those who reached the trial endpoint before 12 months.  A reanalysis of data from those who completed the entire trial (n=259) found that the change in the total UPDRS over 12 months was not significantly different between the treatment groups.  Early treatment with rasagiline did not delay the time until additional therapy was required.  12% (n=49) of participants entered the active treatment phase of the study early as they required additional therapy.  Symptomatic effects may simply be greater if treatment is started earlier. |

| **Trial** | **Pre-defined trial length (months)** | **Percentage of participants** | | | **Did investigators feel disease-modification was demonstrated?** | **Did trial conclusively prove disease-modification?** | **Comments** |
| --- | --- | --- | --- | --- | --- | --- | --- |
|  |  | **Completed follow-up** | **Reached trial endpoint** | **Included in primary analyses** |  |  |  |
| REAL-PET [14] | 24 | 64%  (n=119) | 12%  (n=23) | 68%  (n=127) | Unable to determine this | No | Putamen FDOPA uptake reduced significantly less over two years in those treated with ropinirole than levodopa.  The authors commented that given the comparative design of the study they could not determine if ropinirole was neuroprotective or if levodopa increased the rate of dopaminergic terminal loss.  No correlation between clinical progression [UPDRS (III)] and PET findings.  FDOPA uptake may be affected by ropinirole independent to any effects it has on disease progression. |
| UK/France FDOPA PET [15] | 24 | 87%  (n=39) | n/a | 82%  (n=37) | No | No | No significant difference in the change in putamen FDOPA uptake between those treated with ropinirole and those treated with levodopa monotherapy.  Greater decline in UPDRS (III) in the ropinirole group. |
| Norwegian-Danish [16] | 60 | 50%  (n=81) | 28%  (n=45) | 94%  (n=154) | Yes | No | Significant beneficial effect of selegiline on the primary outcome measures over 60 months.  Only around half of participants completed the full trial period.  No trend towards worsening in the selegiline group during wash-out.  Over a third fewer patients developed motor fluctuations in the selegiline group (just short of statistical significance). However, this may just relate to delayed commencement of levodopa in the selegiline group rather than slowing of disease progression. |
| SELEDO [17] | 60 | 40%  (n=46) | 42%  (n=49) | 92%  (n=107) | Possibly | No | Median time until subjects’ levodopa dose had to be increased by ≥ 50% was significantly longer in the selegiline than the placebo group.  Significantly less deterioration in secondary clinical outcome measures over five years in those treated with selegiline.  However, less than half of those randomised reached the trial endpoint. |
| Swedish selegiline [18] | No set trial length | n/a | 90%  (n=141) | 100%  (n=157) | Yes | No | Selegiline delayed the need for levodopa treatment but there was evidence of a symptomatic effect during the wash-in period.  The selegiline and the placebo groups both deteriorated significantly during the wash-out period, but the magnitude of deterioration was not significantly different between these groups. |

| **Trial** | **Pre-defined trial length (months)** | **Percentage of participants** | | | **Did investigators feel disease-modification was demonstrated?** | **Did trial conclusively prove disease-modification?** | **Comments** |
| --- | --- | --- | --- | --- | --- | --- | --- |
|  |  | **Completed follow-up** | **Reached trial endpoint** | **Included in primary analyses** |  |  |  |
| SINDEPAR [19] | 14* | 81%  (n=82) | n/a | 81%  (n=82) | Yes | No | Significantly less deterioration over 14 months in the total UPDRS with selegiline than placebo, but significant symptomatic effect of selegiline observed during the wash-in period.  The total UPDRS in the selegiline and the placebo groups deteriorated significantly during the wash-out period, but the difference between the groups was not significant. |
| Finnish selegiline [20] | No set trial length | n/a | 87%  (n=47) | 96%  (n=52) | Possibly | No | Selegiline delayed the need for levodopa treatment but there was evidence of a symptomatic effect during the wash-in period.  The authors state that the difference in the initial improvement time between the two groups was not long enough to explain the difference in the delay of the need to start levodopa therapy. |
| Tetrud and Langston [21] | 36 | 0%  (n=0) | 81%  (n=44) | 94%  (n=51) | Yes, probably | No | Selegiline delayed the need for levodopa treatment and no symptomatic benefit was seen during wash-in or wash-out.  Selegiline was felt to have slowed the rate of progression in the five secondary outcomes by 40-83% per year.  It is rather strange that unlike other studies of selegiline no symptomatic effect was seen during the wash-in period.  Fairly short wash-out period which only 70% (n=38) of subjects completed. |
| DATATOP [22] | 24 | 46%  (n=367) | 47%  (n=376) | 100%  (n=800) | α-tocopherol - No  Selegiline - Unclear | α-tocopherol - No  Selegiline - No | Selegiline delayed time until levodopa treatment but evidence of a symptomatic effect during both the wash-in and wash-out periods. |
| QE3 [23] | 16 | 27%  (n=160) | 45%  (n=267) | 50%  (n=300) | No | No | Trial terminated early after a planned interim analysis of 300 patients demonstrated no beneficial effect of treatment with the active-agent when either primary or secondary outcome measures were examined. |
| QE2 [24] | 16 | 36%  (n=29) | 60%  (n=48) | 100%  (n=80) | Yes | No | Dose-ranging study, demonstrating a linear trend between the dose of coenzyme Q_10_ and the change in total UPDRS over 16 months.  A significant difference in the primary outcome was only observed between those receiving the highest dose of coenzyme Q_10_ and those on placebo.  Coenzyme Q_10_ did not significantly delay the need for levodopa.  Examination for sustained divergence in the primary outcome was confounded by using LOCF for those who reached the trial endpoint before 16 months, particularly as only 36% completed the whole follow-up period. |

| **Trial** | **Pre-defined trial length (months)** | | **Percentage of participants** | | | **Did investigators feel disease-modification was demonstrated?** | **Did trial conclusively prove disease-modification?** | **Comments** |
| --- | --- | --- | --- | --- | --- | --- | --- | --- |
|  |  |  | **Completed follow-up** | **Reached trial endpoint** | **Included in primary analyses** |  |  |  |
| Creatine-CoQ10 [25] | 18 | | Unclear | n/a | Unclear | Yes, probably | No | At 18 months the MoCA was significantly higher and plasma phospholipid levels significantly lower in the active-agent than control group.  The authors felt these simultaneous findings suggested combination therapy may have a neuroprotective effect.  No statistical examination of the change in outcome measures over 18 months, only the difference in these measures at that time point. |
| NET-PD LS-1 Creatine [26] | 60-96 | | 39%  (n=685) | n/a | 55%  (n=955) | No | No | No significant difference in terms of the change in the global outcome measure between treatment groups at the time of a planned interim analysis. Therefore, trial terminated early. |
| German Creatine [27] | 24 | | 80%  (n=48) | n/a | 80%  (n=48) | Possibly | No | No overall treatment effect on SPECT variables or on the UPDRS, except for better results in UPDRS (I).  Smaller increase in levodopa equivalent dose in the creatine group than placebo group over the study period.  The authors felt that the greater increase in agonist dose over two years in the placebo group may point to a neuroprotective effect and, due to interactions with the SPECT scans, explain why they failed to show this. |
| Fenugreek [28] | 6 | | 84%  (n=42) | n/a | 84%  (n=42) | Yes | No | No statistically significant difference between treatment groups In terms of the change in the total UPDRS over six months.  However, the authors felt there was a clinically important difference in the total UPDRS and UPDRS (III) between groups and this, taken together with preclinical data, was felt to show a neuroprotective effect. |
| Ubiquinol-10 [29] | | 11 (n=31)  22 (n=33) | 75%  (n=48) | n/a | 75%  (n=48) | No | No | In subjects on levodopa at baseline (n=31), the deterioration in the total UPDRS over 11 months was significantly less in those treated with active-agent than placebo. However, this difference was not maintained following wash-out.  In subjects not on levodopa at baseline (n=33), there was no significant difference between treatment arms in terms of the change in the total UPDRS over 22 months. |

| **Trial** | **Pre-defined trial length (months)** | **Percentage of participants** | | | **Did investigators feel disease-modification was demonstrated?** | **Did trial conclusively prove disease-modification?** | **Comments** |
| --- | --- | --- | --- | --- | --- | --- | --- |
|  |  | **Completed follow-up** | **Reached trial endpoint** | **Included in primary analyses** |  |  |  |
| FAIRPARK [30] | 12 | 85%  (n=34) | n/a | 85%  (n=34) | Yes, probably | No | Using the UPDRS (III), early treatment with deferiprone showed a significant beneficial effect on all three primary endpoints.  After the first 6 months there was a significantly greater decrease in substantia nigra R2* values in the early-start than delayed-start group.  R2* values decreased further in both groups from 6 to 12 months.  The study was extended (out with initial protocol) by 12 months. Patients were randomized to a 6 month washout period at 18 months. Beneficial effects on motor function persisted in those who remained on treatment, but waned in relation to effects seen in the first year of the study. |
| ELLDOPA [31] | 10* | 86%  (n=311) | n/a | 86%  (n=311) | Yes | No | In a dose-response pattern levodopa significantly reduced the decline in the total UPDRS as compared to the placebo group.  The total UPDRS in the three levodopa groups deteriorated during the wash-out period but did not reach the level observed in the placebo group.  The wash-out period was perhaps too short or levodopa induced compensatory mechanisms rather than being neuroprotective.  Significantly greater decline in [^123^I]β-CIT uptake in those treated with levodopa than placebo, perhaps due to confounding effect of levodopa interacting with the dopamine transporter. |
| PROUD [32] | 15 | 73%  (n=390) | n/a | 77%  (n=411) | No | No | No significant difference in change in total UPDRS (and most secondary outcome measures) over 15 months between the early-start and delayed-start treatment groups. |
| GM1 ganglioside [33] | 28 | 78%  (n=60) | n/a | Unclear | Yes, probably | No | Whilst a slight deterioration was observed in the delayed-start group, there was a significant improvement in the UPDRS (III) in the early-start group over the first 6 months. The authors felt this was a symptomatic effect.  The early-start group showed a sustained benefit versus the delayed-start group after 30 months.  There was a significant deterioration in the UPDRS (III) in both the delayed-start and early-start groups during the washout period, and evidence of convergence in the scores of the two groups.  Only 25 subjects (32%) completed the two year wash-out period.  A PET sub-study[34] (n=29) was felt to show a significant slowing of neuronal loss in GM1-treated subjects and, therefore, disease-modification. |

| **Trial** | **Pre-defined trial length (months)** | **Percentage of participants** | | | **Did investigators feel disease-modification was demonstrated?** | **Did trial conclusively prove disease-modification?** | **Comments** |
| --- | --- | --- | --- | --- | --- | --- | --- |
|  |  | **Completed follow-up** | **Reached trial endpoint** | **Included in primary analyses** |  |  |  |
| MitoQ trial [35] | 12 | 65%  (n=85) | 18%  (n=23) | 80%  (n=104) | No | No | No evidence that MitoQ slowed deterioration in the total UPDRS, and no beneficial effects on any secondary outcome measure.  No symptomatic benefit seen at 4 weeks post baseline. |
| Riluzole international [36] | 24 | Unclear | 66%  (n=711) | Unclear | No | No | At interim analysis predefined criteria for futility were met and, therefore, the trial was terminated early. |
| Riluzole USA [37] | 12 | 75%  (n=15) | 65%  (n=13) | 75%  (n=15) | No | No | No significant change observed over 6 months, or after washout, in the primary outcome measures.  No significant difference between early-start or delayed-start groups in outcome measures over 12 months, or in the time until levodopa treatment was required.  Pilot study. |
| GPI-1485  (6 month trial) [38] | 6 | Unclear | n/a | 35%  (n=105)  [imaging analysis] | Yes | No | No significant difference in the change in striatal [^123^I]β-CIT uptake between treatment groups. There was a trend for increased uptake in those treated with high-dose GPI-1485.  No significant difference between groups in terms of the change in the UPDRS (III). |

**Key**

† The percentage shown represents the number of participants completing 24 months of follow-up.

‡ The percentage shown is for the first hierarchical primary outcome. For the second and third hierarchical primary outcomes 85% (n=996) of participants were included in relevant analyses.

§ The paper states that 112 participants received additional therapy by the end of the active phase, while another nine participants received additional therapy before the first active phase assessment or withdrew from the study. These nine participants have been included in the figure for the number of participants reaching the trial endpoint (levodopa/agonist treatment), even though a few may have withdrawn from the study.

‘Pre-defined trial length’ refers to the length of the trial not including any washout period unless the pre-defined primary analyses related to the change in an outcome measure from baseline until the end of the washout period (these studies are marked with an asterisk (*). Similarly, the column relating to the percentage of patients that reached the trial endpoint relates to the number doing so within the pre-defined trial duration, excluding the washout period, unless the pre-defined primary analyses related to the change in characteristics from baseline until the end of the washout period.

The percentage of participants who ‘completed follow-up’ corresponds to the number of patients who continued the study intervention until the end of the double-blind treatment period. Patients who reached a pre-defined endpoint (e.g. commencement of symptomatic treatment or death) which led to their withdrawal from the trial before completing the pre-defined treatment period are not included in this figure.

**Clinical rating scales**

MoCA Montreal Cognitive Assessment [39]

Total UPDRS Total score derived from the Unified Parkinson’s Disease Rating Scale [40]

UPDRS (I) Mentation component of the Unified Parkinson’s Disease Rating Scale

UPDRS (III) Motor component of Unified Parkinson’s Disease Rating Scale

**Biomarker modalities PET ligands**

MRI Magnetic Resonance Imaging FDOPA [^18^F]6-fluoro-L-3,4-dihydroxyphenylalanine

PET Positron Emission Tomography

SPECT Single-Photon Emission Computed Tomography **SPECT ligands**

[^123^I]β-CIT [^123^I]-2β-carbomethoxy-3β-(4-iodophenyl tropane)

**MRI measurements**

R2* Proton transverse relaxation rate

**References**

1. Parkinson Study Group. Mixed lineage kinase inhibitor CEP-1347 fails to delay disability in early Parkinson disease. Neurology. 2007;69:1480-90.
2. Efficacy and safety of green tea polyphenol in de novo Parkinson's disease patients. ClinicalTrials.gov. 2011. http://www.clinicaltrials.gov/ct2/show/NCT00461942. Accessed 22 Sep 2015.
3. Ability to Slow Disease Progression and Safety and Tolerability of Green Tea Polyphenols in Early Parkinson's Disease. The Michael J.Fox Foundation for Parkinson's Research. 2013. https://www.michaeljfox.org/foundation/grant-detail.php?grant_id=187. Accessed 22 Sep 2015.
4. Olanow CW, Schapira AH, Lewitt PA, Kieburtz K, Sauer D, Olivieri G, et al. TCH346 as a neuroprotective drug in Parkinson's disease: a double-blind, randomised, controlled trial. Lancet Neurol. 2006;5:1013-20.
5. Aviles-Olmos I, Dickson J, Kefalopoulou Z, Djamshidian A, Ell P, Soderlund T, et al. Exenatide and the treatment of patients with Parkinson's disease. J Clin Invest. 2013;123:2730-6.
6. Yoritaka A, Takanashi M, Hirayama M, Nakahara T, Ohta S, Hattori N. Pilot study of H_2_ therapy in Parkinson's disease: A randomized double-blind placebo-controlled trial. Mov Disord. 2013;28:836-9.
7. The Parkinson Study Group. Effect of lazabemide on the progression of disability in early Parkinson's disease. Ann Neurol. 1996;40:99-107.
8. Grosset K, Grosset D, Lees A, Parkinson's Disease Research Group of the United Kingdom. Trial of subtherapeutic pergolide in de novo Parkinson's disease. Mov Disord. 2005;20:363-6.
9. Parkinson Study Group. Dopamine transporter brain imaging to assess the effects of pramipexole vs levodopa on Parkinson disease progression. JAMA. 2002;287:1653-61.
10. The Effect of Rasagiline on Cognition in Parkinson's Disease. ClinicalTrials.gov. 2015. http://www.clinIcaltrials.gov/ct2/show/NCT01382342. Accessed 9 Oct 2015.
11. Illarioshkin S, Karabanov A, Mirkasimov A, Verejutina I. Rasagiline in drug-nave Russian patients with early Parkinson's disease. Mov Disord. 2012;27:380.
12. Olanow CW, Rascol O, Hauser R, Feigin PD, Jankovic J, Lang A, et al. A double-blind, delayed-start trial of rasagiline in Parkinson's disease. N Eng J Med. 2009;361:1268-78.
13. Parkinson Study Group. A controlled, randomized, delayed-start study of rasagiline in early Parkinson disease. Arch Neurol. 2004;61:561-6.
14. Whone AL, Watts RL, Stoessl AJ, Davis M, Reske S, Nahmias C, et al. Slower progression of Parkinson's disease with ropinirole versus levodopa: The REAL-PET study. Ann Neurol. 2003;54:93-101.
15. Rakshi JS, Pavese N, Uema T, Ito K, Morrish PK, Bailey DL, et al. A comparison of the progression of early Parkinson's disease in patients started on ropinirole or L-dopa: an 18F-dopa PET study. J Neural Transm. 2002;109:1433-43.
16. Larsen JP, Boas J, Erdal JE. Does selegiline modify the progression of early Parkinson's disease? Results from a five-year study. The Norwegian-Danish Study Group. Eur J Neurol. 1999;6:539-547.
17. Przuntek H, Conrad B, Dichgans J, Kraus PH, Krauseneck P, Pergande G, et al. SELEDO: a 5-year long-term trial on the effect of selegiline in early Parkinsonian patients treated with levodopa. Eur J Neurol. 1999;6:141-150.
18. Palhagen S, Heinonen EH, Hagglund J, Kaugesaar T, Kontants H, Maki-Ikola O, et al. Selegiline delays the onset of disability in de novo parkinsonian patients. Swedish Parkinson Study Group. Neurology 1998;51:520-5.
19. Olanow CW, Hauser RA, Gauger L, Malapira T, Koller W, Hubble J, et al. The effect of deprenyl and levodopa on the progression of Parkinson's disease. Ann Neurol. 1995;38:771-7.
20. Myllyla VV, Sotaniemi KA, Vuorinen JA, Heinonen EH. Selegiline as initial treatment in de novo parkinsonian patients. Neurology. 1992;42:339-43.
21. Tetrud JW, Langston JW. The effect of deprenyl (selegiline) on the natural history of Parkinson's disease. Science. 1989;245:519-22.
22. The Parkinson Study Group. Effects of tocopherol and deprenyl on the progression of disability in early Parkinson's disease. N Eng J Med. 1993;328:176-83.
23. Beal MF, Oakes D, Shoulson I, Henchcliffe C, Galpern WR, Haas R, et al. A randomized clinical trial of high-dosage coenzyme Q10 in early Parkinson disease: no evidence of benefit. JAMA Neurol. 2014;71:543-52.
24. Shults CW, Oakes D, Kieburtz K, Beal MF, Haas R, Plumb S, et al. Effects of coenzyme Q10 in early Parkinson disease: evidence of slowing of the functional decline. Arch Neurol. 2002;59:1541-50.
25. Li Z, Wang P, Yu Z, Cong Y, Sun H, Zhang J, et al. The effect of creatine and coenzyme q10 combination therapy on mild cognitive impairment in Parkinson's disease. Eur Neurol. 2015;73:205-211.
26. Kieburtz K, Tilley BC, Elm JJ, Babcock D, Hauser R, Ross GW, et al. Effect of creatine monohydrate on clinical progression in patients with Parkinson disease: a randomized clinical trial. JAMA. 2015;313:584-93.
27. Bender A, Koch W, Elstner M, Schombacher Y, Bender J, Moeschl M, et al. Creatine supplementation in Parkinson disease: a placebo-controlled randomized pilot trial. Neurology. 2006;67:1262-4.
28. Nathan J, Panjwani S, Mohan V, Joshi V, Thakurdesai PA. Efficacy and safety of standardized extract of Trigonella foenum-graecum L seeds as an adjuvant to L-Dopa in the management of patients with Parkinson's disease. Phytother Res. 2014;28:172-8.
29. Yoritaka A, Kawajiri S, Yamamoto Y, Nakahara T, Ando M, Hashimoto K, et al. Randomized, double-blind, placebo-controlled pilot trial of reduced coenzyme Q10 for Parkinson's disease. Parkinsonism Relat Disord. 2015;21:911-6.
30. Devos D, Moreau C, Devedjian JC, Kluza J, Petrault M, Laloux C, et al. Targeting chelatable iron as a therapeutic modality in Parkinson's disease. Antioxid Redox Signal. 2014;21:195-210.
31. Fahn S, Oakes D, Shoulson I, Kieburtz K, Rudolph A, Lang A, et al. Levodopa and the progression of Parkinson's disease. N Eng J Med. 2004;351:2498-508.
32. Schapira AH, McDermott MP, Barone P, Comella CL, Albrecht S, Hsu HH, et al. Pramipexole in patients with early Parkinson's disease (PROUD): a randomised delayed-start trial. Lancet Neurol. 2013;12:747-55.
33. Schneider JS, Gollomp SM, Sendek S, Colcher A, Cambi F, Du W. A randomized, controlled, delayed start trial of GM1 ganglioside in treated Parkinson's disease patients. J Neurol Sci. 2013;324:140-8.
34. Schneider JS, Cambi F, Gollomp SM, Kuwabara H, Brasic JR, Leiby B, et al. GM1 ganglioside in Parkinson's disease: Pilot study of effects on dopamine transporter binding. J Neurol Sci. 2015;356:118-23.
35. Snow BJ, Rolfe FL, Lockhart MM, Frampton CM, O'Sullivan JD, Fung V, et al. A double-blind, placebo-controlled study to assess the mitochondria-targeted antioxidant MitoQ as a disease-modifying therapy in Parkinson's disease. Mov Disord. 2010;25:1670-4.
36. Rascol O, Olanow W, Brooks D, Koch P, Truffinet R, Bejuit R. A 2-year, multicenter, placebo-controlled, double-blind, parallel-group study of the effect of riluzole on Parkinson's disease progression. Mov Disord. 2002;17:S39.
37. Jankovic J, Hunter C. A double-blind, placebo-controlled and longitudinal study of riluzole in early Parkinson's disease. Parkinsonism Relat Disord. 2002;8:271-6.
38. Guilford Pharmaceuticals Inc: Final phase II GPI 1485 (NIL-A) imaging data presented at the annual meeting of the American Academy of Neurology. PR Newswire. 2002. http://www.prnewswire.co.uk/news-releases/final-phase-ii-gpi-1485-nil-a-imaging-data-presented-at-the-annual-meeting-of-the-american-academy-of-neurology-155593265.html. Accessed 22 Sep 2015.
39. Nasreddine ZS, Phillips NA, Bedirian V, Charbonneau S, Whitehead V, Collin I, et al. The Montreal Cognitive Assessment, MoCA: a brief screening tool for mild cognitive impairment. J Am Geriatr Soc. 2005;53:695-9.
40. Fahn S, Eton RL, UPDRS Development Committee: The Unified Parkinson's Disease Rating Scale. In Recent Developments in Parkinson's Disease. Edited by Fahn S, Marsden CD, Calne D, et al. Florham Park, New Jersey: Macmillan Healthcare Information; 1987. p153-63.
